# Arrestin-biased AT1R agonism induces acute catecholamine secretion through TRPC3 coupling

**DOI:** 10.1038/ncomms14335

**Published:** 2017-02-09

**Authors:** Chun-Hua Liu, Zheng Gong, Zong-Lai Liang, Zhi-Xin Liu, Fan Yang, Yu-Jing Sun, Ming-Liang Ma, Yi-Jing Wang, Chao-Ran Ji, Yu-Hong Wang, Mei-Jie Wang, Fu-Ai Cui, Amy Lin, Wen-Shuai Zheng, Dong-Fang He, Chang-xiu Qu, Peng Xiao, Chuan-Yong Liu, Alex R. B. Thomsen, Thomas Joseph Cahill, Alem W. Kahsai, Fan Yi, Kun-Hong Xiao, Tian Xue, Zhuan Zhou, Xiao Yu, Jin-Peng Sun

**Affiliations:** 1 Key Laboratory Experimental Teratology of the Ministry of Education, Department of Biochemistry and Molecular Biology, Shandong University School of Medicine, 44 Wenhua Xi Road, Jinan, Shandong 250012, China; 2 Department of Physiology, Shandong University School of Medicine, Jinan, Shandong 250012, China; 3 Department of Physiology, Taishan Medical University, Taian, Shandong 271000, China; 4 Duke University, School of Medicine, Durham, North Carolina 27705, USA; 5 Department of Pharmacology, Shandong University School of Medicine, Jinan, Shandong 250012, China; 6 Department of Pharmacology and Chemical Biology, School of Medicine, University of Pittsburgh, Pittsburgh, Pennsylvania 15261, USA; 7 Hefei National Laboratory for Physical Science at Microscale, School of Life Science, University of Science and Technology of China, Hefei, Anhui 230027, China; 8 Laboratory of Cellular Biophysics and Neurodegeneration, Ying-Jie Conference Center, Peking University, Beijing 100871, China

## Abstract

Acute hormone secretion triggered by G protein-coupled receptor (GPCR) activation underlies many fundamental physiological processes. GPCR signalling is negatively regulated by β-arrestins, adaptor molecules that also activate different intracellular signalling pathways. Here we reveal that TRV120027, a β-arrestin-1-biased agonist of the angiotensin II receptor type 1 (AT1R), stimulates acute catecholamine secretion through coupling with the transient receptor potential cation channel subfamily C 3 (TRPC3). We show that TRV120027 promotes the recruitment of TRPC3 or phosphoinositide-specific phospholipase C (PLCγ) to the AT1R-β-arrestin-1 signalling complex. Replacing the C-terminal region of β-arrestin-1 with its counterpart on β-arrestin-2 or using a specific TAT-P1 peptide to block the interaction between β-arrestin-1 and PLCγ abolishes TRV120027-induced TRPC3 activation. Taken together, our results show that the GPCR-arrestin complex initiates non-desensitized signalling at the plasma membrane by coupling with ion channels. This fast communication pathway might be a common mechanism of several cellular processes.

## Abstract (web summary)

Angiotensin II type 1 receptor (AT1R)-mediated acute catecholamine release is modulated by β-arrestin. Here the authors show that β-arrestin-1 recruits the Ca^2+^ channel TRPC3 and the PLCγ to the AT1R-β-arrestin complex, triggering G protein-independent calcium influx and catecholamine secretion.

Activation of G protein-coupled receptors (GPCRs) stimulates both acute and adaptive cellular responses that underlie the reactions of cells to extracellular stimuli and play fundamental roles in almost every aspect of physiological processes, such as neuronal activities, energy homeostasis and immune responses^1^,^2^,^3^,^4^,^5^,^6^. Two general signalling pathways, mediated by either specific G protein subtypes or β-arrestins, are shared by most of the GPCR superfamily members^7^,^8^,^9^,^10^,^11^,^12^. In a traditional view, the receptor–G protein complex residing at the plasma membrane initiates acute signalling events, starting in seconds and activating quick responses, such as vesicle release or constriction of blood vessels, which is often called first-wave signalling^13^,^14^,^15^. Parallel to G protein signalling, the ligand-induced active receptor conformation or the phosphorylated receptor is recognized by arrestin, which not only mediates receptor endocytosis but also triggers a second wave of GPCR signalling localized at the endosomes^15^. Compared with the receptor–G protein signalling at the plasma membrane, the second wave of GPCR signalling mediated by arrestin, including extracellular signal-regulated kinase and c-Jun N-terminal kinase 3 activation^16^,^17^, commonly occurs 2–3 min later and plays critical roles in long-term GPCR functions. This traditional thinking has recently been challenged by the detection of receptor-G protein signalling occurring at endosomes, which is termed third-wave signalling^13^. However, evidence for acute GPCR functions other than the desensitization mediated by arrestins remains lacking, although arrestin emerged earlier than G proteins during evolution^18^.

In the present study, we use specific G protein or β-arrestin-biased GPCR agonists and knockout mouse models to investigate the role of β-arrestin or G protein subtype-mediated GPCR signalling in catecholamine secretion after angiotensin II receptor type 1 (AT1R) activation, which may mimic clinical conditions, such as hypertension or response to severe haemorrhage^19^. We examine the effects of the preclinical drug TRV120027 (also known as TRV027) on catecholamine secretion to evaluate its effect on adrenal medulla function^20^,^21^,^22^. Notably, not only the Gq-PLCβ-mediated pathway but also β-arrestin-biased agonism are capable of stimulating acute hormone secretion. Pharmacological interventions, together with electrophysiological experiments in wild-type or *TRPC3*^*−/−*^*/TRPC6*^*−/−*^*/TRPC7*^*−/−*^ knockout mice, unambiguously demonstrate arrestin-mediated catecholamine secretion through direct coupling with transient receptor potential cation channel subfamily C 3 (TRPC3) and extracellular calcium influx. In recombinant systems, application of the β-arrestin-biased agonist TRV120027 promotes the formation of a macromolecular complex composed of AT1R–β-arrestin-1–TRPC3–PLCγ at the plasma membrane. TRV120027 also elicits specific conformational changes in TRPC3 that require recognition of the C-terminal IP3-R-binding motif of TRPC3 by the β-arrestin-1 C-terminal region and recruitment of phosphoinositide-specific phospholipase C (PLCγ) through poly-proline region1 in the N-terminal region of β-arrestin-1. Swapping the C-terminal sequence between the β-arrestin-1 and the β-arrestin-2 changes their TRPC3-coupling activity, and a specific TAT-P1 peptide abolishes the TRV120027-induced TRPC3 activation. The receptor-β-arrestin-1-TRPC3 signalling is comparable to that of classic receptor-Gq-IP3R pathways in the regulation of calcium mobilization and vesicle secretion in the adrenal medulla by endogenous hormones, including angiotensin II (AngII) and muscarinic acetylcholine (Mch). We demonstrate that receptor–β-arrestin-1–TRPC3 signalling constitutes an important component after the activation of endogenous AT1R or Mch receptors. These results indicate that arrestin-mediated ion channel coupling could be a general mechanism for at least a subset of GPCRs. Taken together, our results suggest an additional mode of arrestin-mediated GPCR signalling to regulate several physiological processes, involving a fast crosstalk between receptors and ion channels through arrestins.

## Results

### β-arrestin-1 agonism induced acute catecholamine secretion

**Figure 1:**
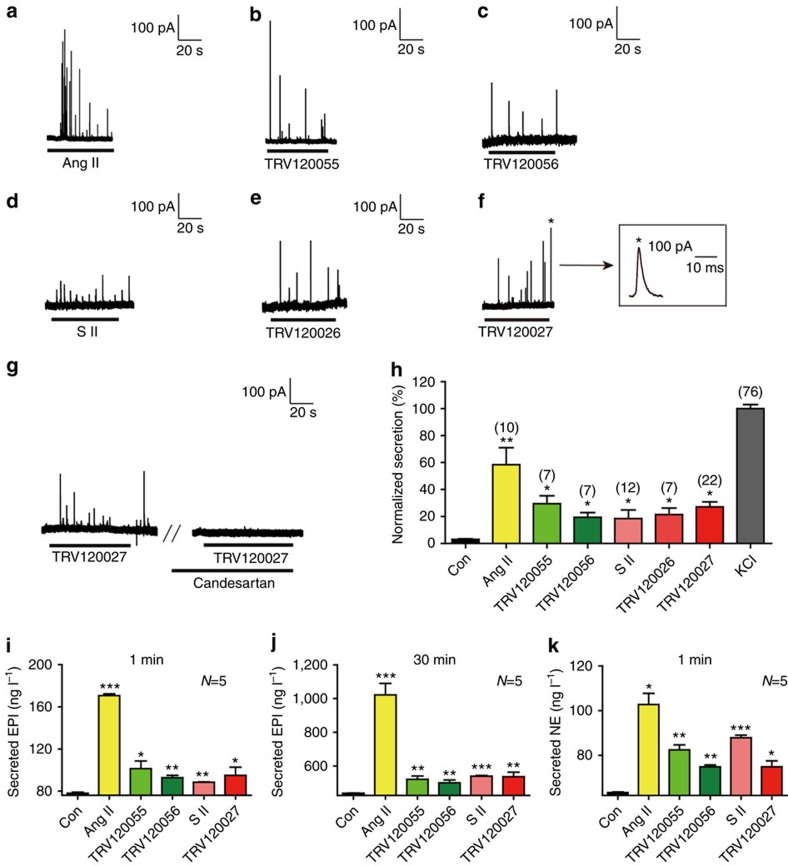
β-arrestin-biased AT1R agonists stimulate acute catecholamine secretion. (**a**) AngII (100 nM) stimulates amperometric spikes of primary chromaffin cells measured with a microCFE. (**b**,**c**) G protein-biased A1TR agonists, including TRV120055 (30 nM) and TRV120056(50 nM), stimulate amperometric spikes of primary chromaffin cells. (**d**–**f**) β-arrestin-biased A1TR agonists, including SII (1 μM), TRV120026 (500 nM) and TRV120027 (100 nM), stimulate amperometric spikes of primary chromaffin cells. (**g**) TRV120027 (100 nM)-stimulated amperometric spikes of primary chromaffin cells are blocked by the AT1R antagonist Candesartan (100 nM). (**h**) Summary bar graph of acute catecholamine secretion induced by different AT1R agonists of chromaffin cells. The amounts of catecholamine secretion were evaluated by the integral of the amperometry. (**i**) Different AT1R agonists induced epinephrine secretion in the adrenal medulla, measured by an ELISA Kit at 1 min. (**j**) Different AT1R agonists induced epinephrine secretion in the adrenal medulla at 30 min. Potassium chloride-stimulated epinephrine secretion was used as a control. (**k**) Different AT1R agonists induced norepinephrine secretion in the adrenal medulla, measured with an ELISA Kit at 1 min. (**h**–**k**) *P<0.05; **P<0.01; ***P<0.005; different AT1R agonist treatments were compared with control vehicles. The bars represent mean±s.d. The data statistics were analysed using one-way ANOVA.

To investigate the function of G protein- or β-arrestin-biased agonism in acute catecholamine secretion, we treated primary chromaffin cells with a panel of AT1R agonists that had a broad spectrum of biased molecular efficacies towards either Gq-PLCβ or β-arrestin signalling^8^,^21^,^22^,^23^ and monitored the secretion by a micro-carbon fibre electrode (CFE)^3^ (Table 1 and Supplementary Fig. 1a–c and Supplementary Table 1). The TRV120055 and TRV120056 are often regarded as super Gq AT1R agonists and can generate only G protein signalling stimulated with low concentration(Table 1 and Supplementary Fig. 1). Notably, similar to the full AT1R agonist AngII, both the Gq-biased agonists (TRV120055, TRV120056) and the β-arrestin-biased agonists (SII, TRV120026 and TRV120027) stimulated typical amperometric current traces, which indicated catecholamine secretion from chromaffin cells (Fig. 1a–f,h). Notably, the β-arrestin-biased agonists, such as SII, TRV120026 and TRV120027, induced little or no IP1 production, and TRV120027 was regarded as a Gq-PLCβ pathway antagonist^23^. Therefore, the arrestin-biased agonism, such as that activated by TRV120027 induced acute catecholamine secretion in a Gq-PLC pathway independent manner. By contrast, TRV120027-induced secretion was totally abolished by the AT1R-specific antagonist candesartan, indicating that TRV120027 stimulates the chromaffin cell secretion through the activation of AT1R (Fig. 1g). Similar to the phenomena observed in single cells, 100 nM TRV120027 significantly stimulated both epinephrine and norepinephrine secretion, in addition to their autonomous spontaneous catecholamine releases of the isolated adrenal medulla, confirming the importance of the β-arrestin-biased agonism in a more physiological context (Fig. 1i–k and Supplementary Fig. 2). It is also worthwhile to note that the AT1R agonists induced different patterns regarding the secretion of epinephrine or norepinephrine, which indicated the existence of different mechanisms to control the secretion of these hormones.

**Figure 2:**
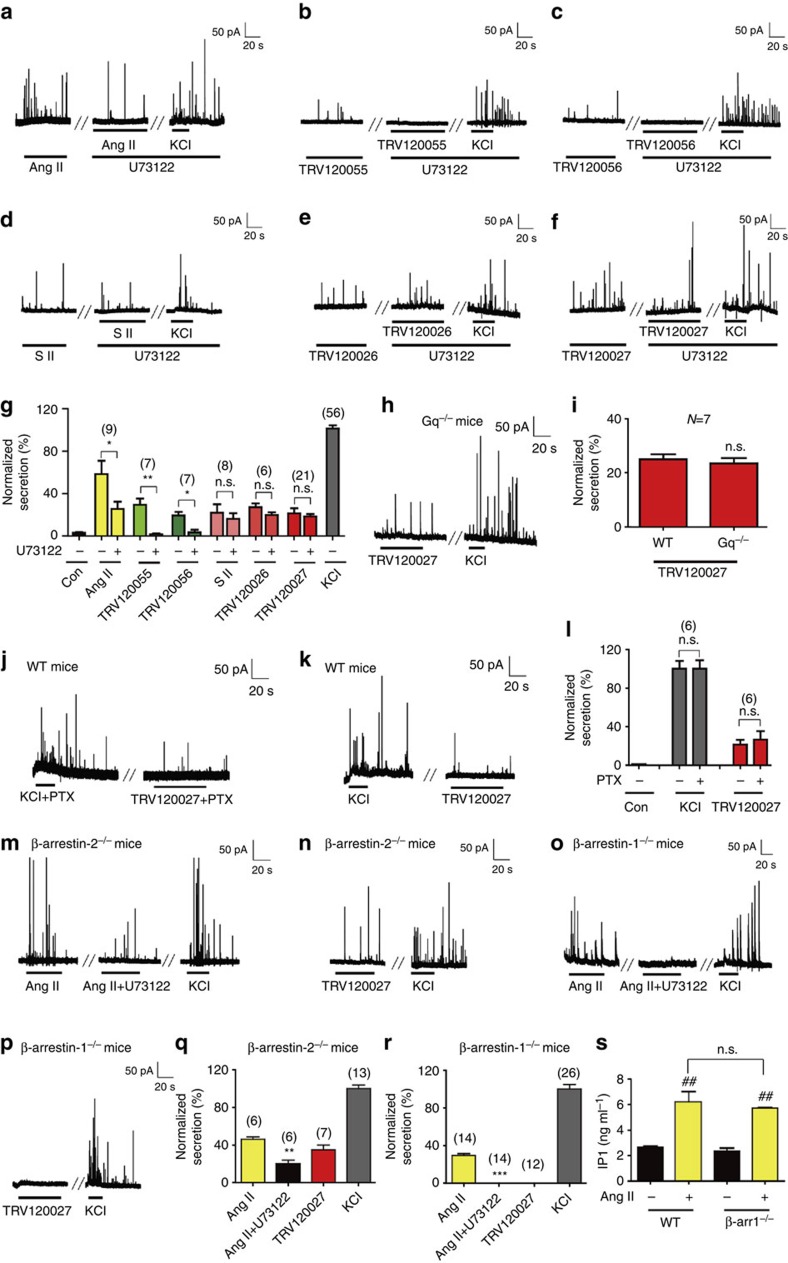
Contribution of G protein or β-arrestin pathways in catecholamine secretion. (**a**) Effect of the PLC inhibitor U73122 (10 μM) on AngII-induced amperometric spikes of chromaffin cells. (**b**,**c**) Amperometric spikes of chromaffin cells stimulated by G protein-biased A1TR agonists, including TRV120055 (30 nM) and TRV120056 (50 nM), were blocked by the PLC inhibitor U73122. (**d**–**f**) PLC inhibitor U73122 had no effect on the secretion of chromaffin cells stimulated by β-arrestin-biased A1TR agonists, including SII (1 μM), TRV120026 (500 nM) and TRV120027 (100 nM). (**g**) Summary bar graph of the effect of U73122 on chromaffin cell secretion induced by AngII, TRV120055, TRV120056, SII, TRV120026 and TRV120027. (**h**) TRV120027-induced amperometric spikes of chromaffin cells derived from *Gq*^*−/−*^ mice. (**i**) Summary bar graph of acute catecholamine secretion induced by TRV120027 treatment of the chromaffin cells of *Gq*^*−/−*^ mice and their wild-type littermates. (**j**,**k**) Effect of the Gi inhibitor pertussis toxin (PTX) on high potassium chloride or TRV120027-induced amperometric spikes. Cells were pre-incubated with PTX (**j**) or vehicle (**k**). (**l**) Statistical analysis and bar graph representation of the effect of PTX on high potassium chloride or TRV120027-induced catecholamine secretion (**j**,**k**). (**m**) Effect of U73122 on amperometric spikes induced by AngII in primary chromaffin cells of *β-arrestin-2*^*−/−*^ mice. (**n**) TRV120027-induced amperometric spikes in chromaffin cells of *β-arrestin-2*^*−/−*^ mice. (**o**) U73122 blocked amperometric spikes induced by AngII in primary chromaffin cells of *β-arrestin-1*^*−/−*^ mice. (**p**) β-arrestin-biased agonist TRV120027 did not induce amperometric spikes in chromaffin cells of *β-arrestin-1*^*−/−*^ mice. (**q**,**r**) Summary bar graph of the AngII and TRV120027-induced amperometric spikes in chromaffin cells of *β-arrestin-2*^*−/−*^ mice (**m**,**n**) or *β-arrestin-1*^*−/−*^ mice (**o**,**p**). (**s**) IP1 elevation induced by AngII (100 nM) in the primary chromaffin cells derived from the WT or *β-arrestin-1*^*−/−*^ mice. The measurements were carried out with an ELISA Kit at 1 min. ^##^*P*<0.01; AngII treatments were compared with control vehicles. (**g**,**i**,**n**,**o**) *P<0.05; **P<0.01; ***P<0.005; U73122 treatments were compared with control vehicles. ns, no significant difference. The bars represent mean±s.d. All statistics were analysed using one-way ANOVA.

To dissect how the G protein pathway or specific arrestin subtype-mediated signalling contribute to acute catecholamine secretion after AT1R activation, we examined the effects of different AT1R-biased ligands on primary chromaffin cells using *Gq*^*−/−*^*, β-arrestin-1*^*−/−*^ *or β-arrestin-2*^*−/−*^ mice and pharmacological blockers (Supplementary Figs 3,4 and 6) Whereas application of the PLC inhibitor U73122 almost abolished the amperometric spikes induced by the Gq-biased agonists TRV120055 or TRV120056 and decreased AngII-mediated secretion by approximately half, it had no significant effects on catecholamine secretion stimulated by β-arrestin-biased agonists, such as SII, TRV120026 or TRV120027 (Fig. 2a–g). Moreover, TRV120027 induced amperometric current traces to a similar extent in chromaffin cells that were derived from *Gq*^*−/−*^ mice as in those derived from their wild-type littermates, confirming that TRV120027-induced catecholamine secretion is independent of the Gq pathway (Fig. 2h,i). Activation of AT1R is reportedly coupled to Gi. However, the effect of TRV120027 was not significantly decreased by pertussis toxin treatment (Fig. 2j–l), consistent with previous observations that Gi-mediated AT1R signalling did not promote catecholamine secretion^24^,^25^. Importantly, whereas 100 nM AngII or TRV120027 induced secretion of chromaffin cells in *β-arrestin-2*^*−/−*^ mice, the application of TRV120027 was not able to stimulate amperometric currents, and AngII-induced catecholamine secretion was totally blocked by U73122 in chromaffin cells derived from *β-arrestin-1*^*−/−*^ mice (Fig. 2m–r and Supplementary Fig. 5). The effect of the β-arrestin-1 deficiency on TRV120027 induced catecholamine secretion is not due to the impairment of the Gq signalling, as chromaffin cells derived from the *β-arrestin-1*^*−/−*^ mice produced similar IP1 as wild-type mice in response to AngII stimulation (Fig. 2s). Taken together, these results demonstrated that Gq-PLCβ- and β-arrestin-1-dependent signalling-coordinated regulation of AngII-stimulated chromaffin cell secretion. In particular, TRV120027 induced acute catecholamine secretion through the specific β-arrestin-1 subtype-biased agonism that is independent of G protein signalling.

### β-arrestin-1-mediated secretion required calcium influx

**Figure 3:**
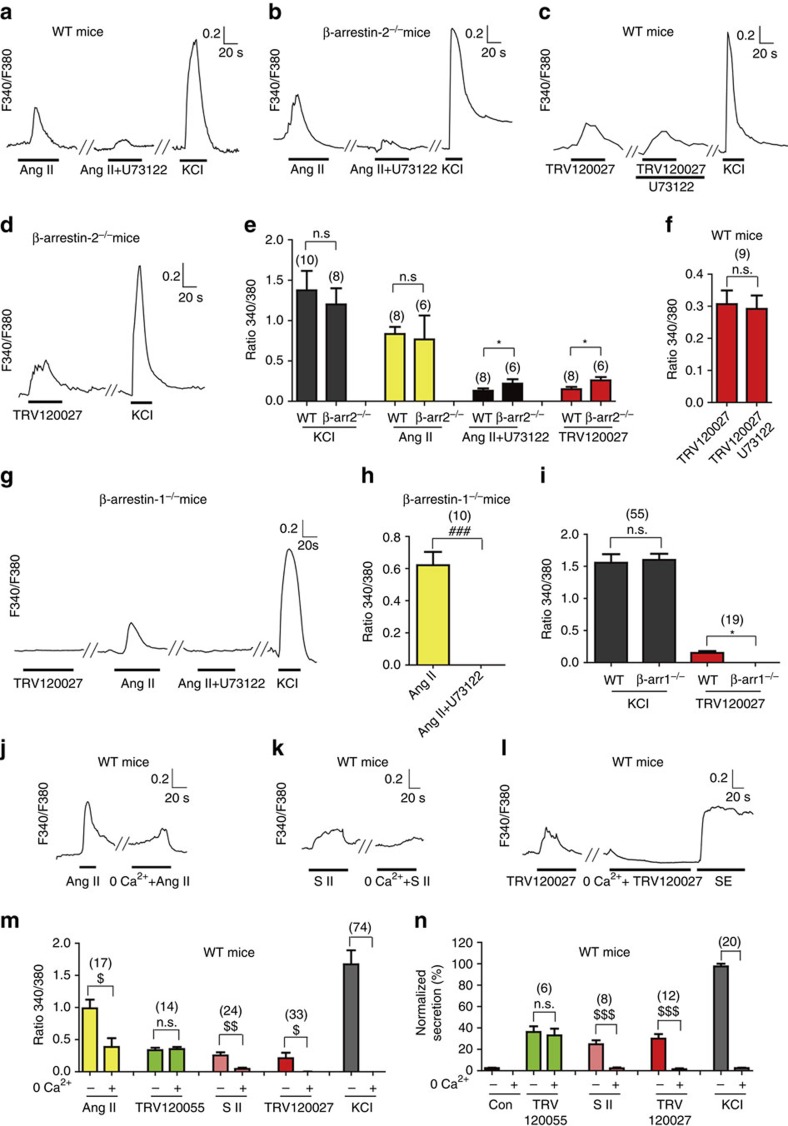
β-arrestin- biased signalling stimulates secretion through calcium influx. (**a**,**b**) AngII (100 nM) stimulates [Ca^2+^]_i_ elevation in the primary chromaffin cells of WT (**a**) or *β-arrestin-2*^*−/−*^ mice (**b**), which was not completely blocked by U73122(10 μM). (**c**) TRV120027 stimulates [Ca^2+^]_i_ elevation in the primary chromaffin cells of WT was not affected by U73122 (10 μM). (**d**)TRV120027 (100 nM) induced [Ca^2+^]_i_ elevation in primary chromaffin cells of *β-arrestin-2*^*−/−*^mice. (**e**) Summary bar graph of [Ca^2+^]_i_ responses to high potassium chloride (70 mM), AngII (100 nM) or TRV120027 (100 nM) in primary chromaffin cells of wild-type or *β-arrestin-2*^*−/−*^ mice. (**f**) Bar graph representation and statistical analysis of the TRV120027-induced [Ca^2+^]_i_ elevation in the primary chromaffin cells with or without U73122 incubation (**c**). (**g**) Effects of TRV120027 (100 nM), AngII (100 nM) on [Ca^2+^]_i_ in primary chromaffin cells of *β-arrestin-1*^*−/−*^ mice. (**h**) Bar graph representation and statistical analysis of the AngII-induced [Ca^2+^]_i_ elevation in the primary chromaffin cells derived from *β-arrestin-1*^*−/−*^ mice with or without U73122 incubation (**f**). (**i**) Summary bar graph of [Ca^2+^]_i_ responses to high potassium chloride (70 mM) or TRV120027 (100 nM) in primary chromaffin cells of wild-type or *β-arrestin-1*^*−/−*^ mice. (**j**) AngII (100 nM) induced [Ca^2+^]_i_ responses of primary chromaffin cells in the Ca^2+^-deficient bath. (**k**,**l**) SII- (1 μM; **k**) or TRV120027- (100 nM; **l**) induced [Ca^2+^]_i_ elevation in chromaffin cells was abolished in a Ca^2+^-deficient bath. (**m**) Summary bar graph of AngII- (100 nM), TRV120055- (30 nM), SII- (1 μM) or TRV120027- (100 nM) stimulated [Ca^2+^]_i_ responses in the Ca^2+^-deficient bath. (**n**) Summary bar graph of TRV120055- (30 nM), SII- (1 μM) or TRV120027- (100 nM) induced acute catecholamine secretion in a Ca^2+^-deficient bath compared with normal medium. The catecholamine secretions were monitored by the integral of the amperometry, and 70 mM potassium chloride was used as a control. (**e** or **i**) *P<0.05; *β-arrestin-1*^*−/−*^or *β-arrestin-2*^*−/−*^ mice were compared with wild-type littermates. (**f** or **h**) ^###^*P*<0.005; U73122-treated cells were compared with the vehicle-treated control cells. ns, no significant difference between U73122-treated cells and control vehicles. (**m** or **n**) ^$^*P*<0.05; ^$$^*P*<0.01; ^$$$^*P*<0.005; chromaffin cells in the Ca^2+^-deficient bath were compared with Hank's buffered solutions. The bars represent mean±s.d. All data statistics were analysed using one-way ANOVA.

Calcium is a critical second messenger that has been reported to couple many extracellular stimuli to processes of hormone release^25^. We therefore used the F340/F380 ratio of Fura-2 intensity to monitor the intracellular calcium change challenged by a spectrum of AT1R-biased agonists^1^,^3^. In primary chromaffin cells, a fraction of the AngII-induced [Ca^2+^]_i_ signals was still observed by the application of the PLC inhibitor U73122 (Fig. 3a,b,e). Moreover, treating cells with the Gq pathway antagonist TRV120027 significantly stimulated a [Ca^2+^]_i_ increase in primary chromaffin cells that were derived from either wild-type or *β-arrestin-2*^*−/−*^ mice. No effect was observed upon the administration of U73122 to the wild-type mice (Fig. 3c–f). Notably, in *β-arrestin-1*^*−/−*^ mice, TRV120027 was unable to induce a [Ca^2+^]_i_ increase, and the AngII-induced [Ca^2+^]_i_ increase was completely blocked by U73122 (Fig. 3g–i). Taken together, these data suggest that a β-arrestin-1-dependent mechanism other than the activation of the Gq-PLC-IP3-sensitive Ca^2+^ stores was involved in mediating the [Ca^2+^]_i_ increase after TRV120027-induced AT1R activation.

We next examined the contribution of extracellular Ca^2+^ influx in the [Ca^2+^]_i_ increase downstream of AT1R activation in primary chromaffin cells. In the Ca^2+^-deficient bath, the intracellular [Ca^2+^] increases induced by AngII, SII and TRV120027 were reduced by ∼50%, 90% and 100%, respectively, whereas TRV120055-induced intracellular [Ca^2+^] elevation was not affected (Fig. 3j–m and Supplementary Fig. 7). The fraction of the residual [Ca^2+^]_i_ increase in the Ca^2+^-deficient bath corresponded to the efficacy of the agonist-induced Gq-PLC pathway activation (Table 1)^8^. These data indicated that the β-arrestin-biased, agonist-induced intracellular [Ca^2+^] increase occurred mainly through the extracellular Ca^2+^ influx. Moreover, in contrast to TRV120055-stimulated secretion, the SII- or TRV120027-induced catecholamine secretions were completely abolished by the extracellular Ca^2+^ deficiency, confirming that the β-arrestin-biased agonism-stimulated chromaffin cell secretions were mediated by extracellular Ca^2+^ influx (Fig. 3n and Supplementary Fig. 8).

### β-arrestin-1-biased agonism activated TRPC3

**Figure 4:**
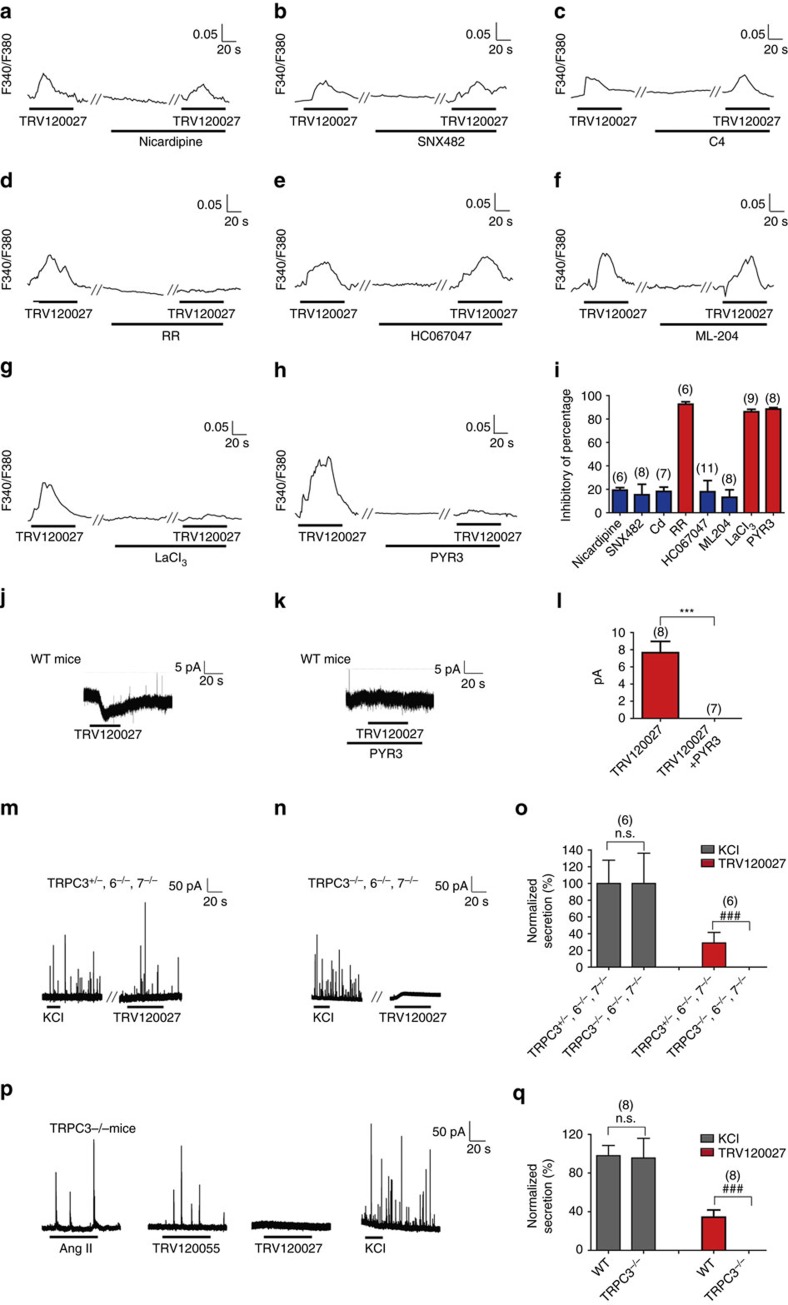
β-arrestin-1-biased signalling stimulates secretion through TRPC3 activation. (**a**–**h**) Effects of different calcium channel blockers on TRV120027- (100 nM) induced [Ca^2+^]_i_ elevation in primary chromaffin cells. Representative [Ca^2+^]_i_ responses are shown for nicardipine (10 μM), a L-type calcium channel blocker (**a**); SNX482 (0.1 μM), a R-type calcium channel blocker (**b**); 100 μM [Cd^2+^], a nonspecific voltage-dependent calcium blocker (**c**); ruthenium red (10 μM), a nonspecific TRP channel blocker (**d**); HC067047 (0.3 μM), a selective TRPV4 blocker (**e**); ML-204 (10 μM), a selective TRPC4 blocker (**f**); lanthanum chloride (100 μM), a non-selective TRPC3/6/7 blocker (**g**); and Pyr3(10 μM), a selective TRPC3 blocker (**h**). (**i**) Summary bar graph of the percentage of the blockade in TRV120027- (100 nM) induced [Ca^2+^]_i_ elevation by the presence of different calcium channel blockers. (**j**) An example of inward current in a single primary mouse chromaffin cell that was induced by TRV120027 when bathed in an extracellular solution with 1 μM tetrodotoxin (TTX) and 1 μM 4-AP and voltage-clamped at −70 mV. (**k**) The TRV120027-induced inward current in chromaffin cells was blocked by Pyr3. (**l**) Statistical analysis and bar graph representation of the TRV120027-induced current in primary chromaffin cells with or without Pyr3 incubation. ***P<0.005; chromaffin cells pre-incubated with Pyr3 were compared with control vehicles. (**m**,**n**) Representative high potassium chloride or TRV120027 (100 nM)-induced amperometric spikes of primary chromaffin cells derived from *TRPC3*^*+/−*^*/TRPC6*^*−/−/*^*TRPC7*^*−/−*^ mice (**m**) and *TRPC3*^*−/−*^*TRPC6*^*−/−/*^*TRPC7*^*−/−*^ mice (**n**). (**o**) Summary bar graph of TRV120027- (100 nM) or high potassium chloride-induced catecholamine secretion of chromaffin cells from *TRPC3*^*+/−*^*TRPC6*^*−/−*^*TRPC7*^*−/−*^ and *TRPC3*^*−/−*^*TRPC6*^*−/−*^*TRPC7*^*−/−*^ mice (**m**,**n**). (**p**) Representative AngII (100 nM), TRV120055 (30 nM), TRV120027 (100 nM) or high potassium chloride-induced amperometric spikes in primary chromaffin cells derived from *TRPC3*^*−/−*^ mice. (**q**) Summary bar graph of TRV120027 (100 nM)—or high potassium chloride-induced catecholamine secretion of chromaffin cells from *TRPC3*^*−/−*^ mice. (**o**,**q**) ^###^*P*<0.005; chromaffin cells derived from *TRPC3*^*−/−*^*TRPC6*^*−/−/*^*TRPC7*^*−/−*^ mice were compared with those of the *TRPC3*^*+/−*^*TRPC6*^*−/−/*^*TRPC7*^*−/−*^ littermates and chromaffin cells derived from *TPRC3*^*−/−*^ mice were compared with those of the wild-type littermates. The bars represent mean±s.d. All statistics were analysed using one-way ANOVA.

The activation of Ca^2+^ channels or transporters may mediate the extracellular Ca^2+^ influx stimulated by TRV120027. We therefore screened a series of channel blockers and monitored their effects on TRV120027-induced [Ca^2+^]_i_ increases. Application of the L-type Ca^2+^ channel blocker nicardipine, the R-type Ca^2+^ channel blocker SNX482 or the nonspecific voltage-dependent calcium blocker [Cd^2+^] (100 μM) had no significant effects on TRV120027-induced [Ca^2+^]_i_ increases (Fig. 4a–c,i). However, the nonspecific TRP channel blocker ruthenium red completely blocked [Ca^2+^]_i_ increases stimulated by TRV120027 (Fig. 4d,i). We then applied different TRP channel blockers to clarify the specific TRP channel identity activated by TRV120027. Whereas the TRPA1 blocker HC-030031, TRPV1 antagonist SB366791, TRPV4 blocker HC067047 and the TRPC4 blocker ML-204 had no significant effects, the TRPC3/6/7 blocker lanthanum chloride (100 μM) and the TRPC3-specific blocker Pyr3 completely eliminated the TRV120027-induced [Ca^2+^]_i_ increase (Fig. 4e–i and Supplementary Fig. 9a–c). In contrast, the TRP channel blockers, including ruthenium red, lanthanum chloride and Pyr3, which show great inhibitory activity towards TRV120027-induced [Ca^2+^]_i_ increases, had no significant effect on the TRV120055-stimulated calcium signal. This suggested that these two AT1R agonists induced an intracellular calcium increase via distinct mechanisms (Supplementary Figs 10 and 11). Moreover, the application of TRV120027 to primary chromaffin cells clamped at −70 mV induced an inward current that was specifically blocked by Pyr3, demonstrating that TRPC3 was activated after TRV120027 administration (Fig. 4j–l). Finally, whereas the TRV120027-stimulated catecholamine secretion in primary chromaffin cells derived from *TRPC3*^*+/−*^*TRPC6*^*−/−*^*TRPC7*^*−/−*^ mice or wild-type mice, it lost its effect in *TRPC3*^*−/−*^*TRPC6*^*−/−*^*TRPC7*^*−/−*^ or *TRPC3*^*−/−*^ mice (Fig. 4m–q and Supplementary Figs 12 and 13). In contrast, the high potassium-induced catecholamine secretion was not significantly different between the *TRPC3*^*+/−*^*TRPC6*^*−/−*^*TRPC7*^*−/−*^ and the *TRPC3*^*−/−*^*TRPC6*^*−/−*^*TRPC7*^*−/−*^ mice or between the *TRPC3*^*−/−*^ mice and their wild-type littermates (Fig. 4o,q). Although the AngII-induced amperometric current was significantly decreased in the *TRPC3*^*−/−*^ mice compared with their wild-type littermates, the TRV12005-induced catecholamine secretion was not affected (Supplementary Fig. 14). Taken together, these pharmaceutical interventions and animal studies revealed that the β-arrestin-1-biased agonism induced acute catecholamine secretion through the activation of TRPC3 in chromaffin cells.

### AT1R–β-arrestin-1–TRPC3 complex formation

**Figure 5:**
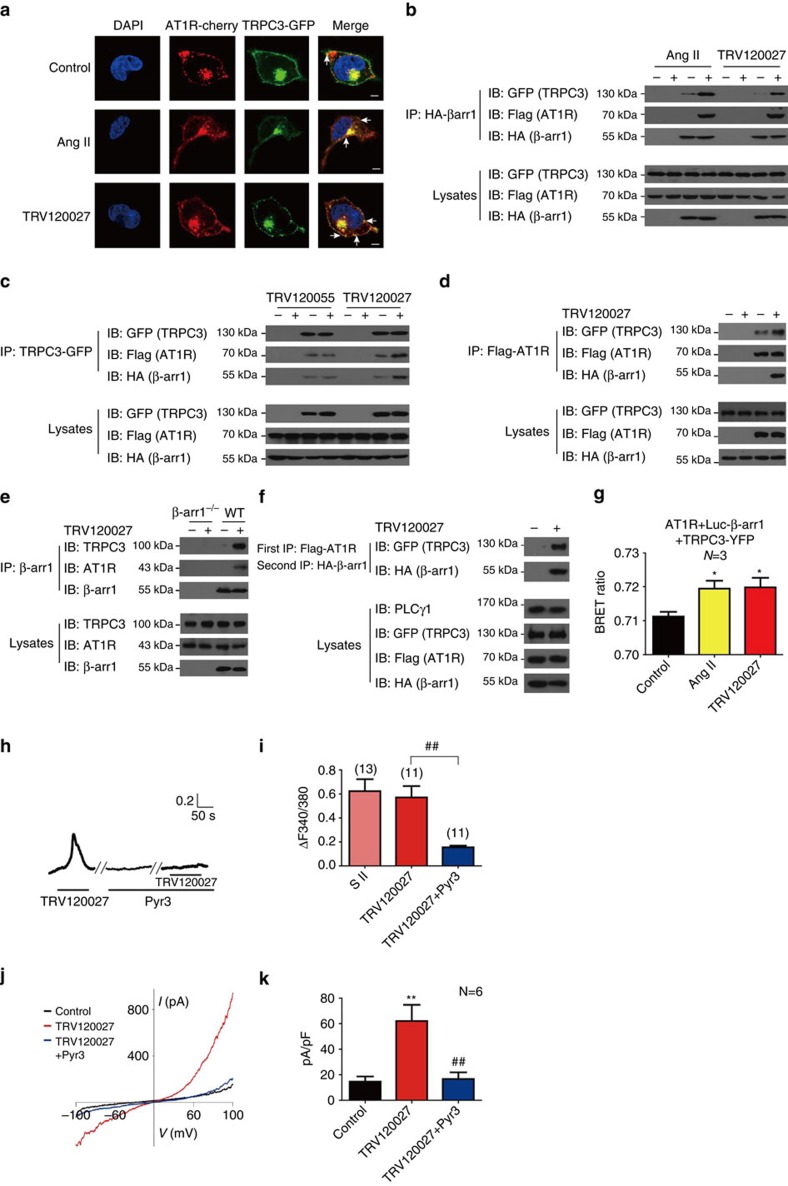
TRV120027 induced AT1R-β-arrestin-1-TRPC3 complex formation. (**a**) HEK293 cells co-transfected with Flag-AT1R-Cherry, HA-β-arrestin-1 and TRPC3-GFP were stimulated with AT1R ligands for 1 min. Co-localization of AT1R and TRPC3 was determined. (**b**–**d**) HEK293 cells co-transfected with Flag-AT1R-cherry, HA-β-arrestin-1 and TRPC3-GFP were stimulated with different agonists for 1 min. (**b**) HA-tagged β-arrestin-1 was immunoprecipitated by anti-HA agarose, and co-precipitated AT1R and TRPC3 were examined by specific anti-Flag or anti-GFP antibodies. (**c**) TRPC3-GFP was immunoprecipitated by anti-GFP agarose, and the receptor or β-arrestin-1 interactions was examined by western blotting. (**d**) Flag-AT1R-cherry was immunoprecipitated by anti-Flag agarose, and the presence of β-arrestin-1 and TRPC3 was examined by western blotting. (**e**) After 100 nM TRV120027 administration in the adrenal medulla, the anti-β-arrestin-1 antibody A1CT was used to immunoprecipitate the endogenous β-arrestin-1. The association of AT1R or TRPC3 with β-arrestin-1 was detected by specific antibody. (**f**) HEK293 cells co-transfected with Flag-AT1R-cherry, HA-β-arrestin-1 and TRPC3-GFP were stimulated for 1 min. The sequential immunoprecipitation was performed and the TRPC3-GFP associated with the Flag-AT1R-HA-β-arrestin-1 complex was examined. (**g**) HEK293 cells co-transfected with Flag-AT1aR, Luc-β-arrestin-1 and TRPC3-YFP were stimulated with AngII (100 nM) or TRV120027 (100 nM) for 1 min. The BRET signals were measured to detect the interaction between the β-arrestin-1 and TRPC3. (**h**) Representative [Ca2+]i responses of HEK293 cells co-transfected with Flag-AT1R-Cherry, HA-β-arrestin-1 and TRPC3-GFP to TRV120027 (100 nM), with or without Pyr3 incubation. (**i**) Summary bar graph of TRV120027 (100 nM) induced [Ca^2+^]_i_ elevation and blockade by Pyr3, as described in **h**. (**j**) The resultant *I–V* relationships of current responses to voltage steps of HEK293 cells co-transfected with Flag-AT1R-Cherry, HA-β-arrestin-1 and TRPC3-GFP and stimulated with TRV120027. (**k**) Statistical analysis and bar graph representation of the current responses to TRV120027 administration with or without Pyr3 incubation. (**g** or **k**) *P<0.05; **P<0.01; different AT1aR agonist treatments were compared with control vehicles. (**i,j** or **k**) ^##^*P*<0.01; chromaffin cells pre-incubated with Pyr3 were compared with control vehicles. The bars represent mean±s.d. All data statistics were analysed using one-way ANOVA. Scale bars, 10 μm (**a**).

After activation, specific ligand-induced GPCR conformations or particular phosphorylation barcodes dictate selective β-arrestin conformations to form distinct functional complexes that underlie many GPCR functions^26^,^27^. We therefore investigated whether β-arrestin-1 forms a functional complex with AT1R and TRPC3 by co-immunoprecipitation, sequential co-immunoprecipitation, BRET (bioluminescence resonance energy transfer) and electrophysiology. The distributions of AT1R and TRPC3 were first examined by confocal immunofluorescence microscopy before and after agonist stimulation. Before the exposure to agonists, AT1R and TRPC3 were mainly localized in the plasma membrane. Administration of TRV120027 for 1 min significantly increased the co-localization of AT1R and TRPC3 in the plasma membrane region (Fig. 5a). Concurrently, a portion of β-arrestin-1 was recruited to plasma membrane clusters using a recently developed BRET assay (Supplementary Fig. 15)^28^. We then used co-immunoprecipitation and sequential co-immunoprecipitation to determine whether TRV120027 promoted the constitution of the AT1R, β-arrestin-1 and TRPC3 in the same macromolecular complex. Stimulation with either AngII or TRV120027 significantly increased the AT1R and TRPC3 association with the immunoprecipitated β-arrestin-1 (Fig. 5b and Supplementary Figs 16 and 17). Conversely, immunoprecipitation of either AT1R or TRPC3 increased the association with the other protein and β-arrestin-1 after TRV120027 administration for 1 min (Fig. 5c,d and Supplementary Figs 16 and 17). In the isolated adrenal medulla, TRV120027 increased the association between endogenous β-arrestin-1 and TRPC3 and between β-arrestin-1 and AT1R (Fig. 5e and Supplementary Fig. 18). Moreover, TRV120027 promoted the recruitment of the TRPC3 to AT1R-β–arrestin-1 in the sequential co-immunoprecipitation assay (Fig. 5f and Supplementary Fig. 19). Finally, we measured the interaction of β-arrestin-1 and TRPC3 through BRET assay. At 1 min time point, both TRV120027 and AngII promoted significant β-arrestin-1–TRPC3 association (Fig. 5g). In contrast, the application of AngII or TRV120027 had no significant effect on the BRET signal between the TRPC3-β–arrestin-2, TRPV1-β–arrestin-1 or TRPC6–β-arrestin-1 pairs, suggesting a specific interaction of TRPC3-β-arrestin-1 downstream of AT1R (Supplementary Fig. 20). Taken together, these results indicated that TRV120027 stimulation promoted AT1R–β-arrestin-1–TRPC3 complex formation in a quick manner, as rapidly as 1 min.

To further confirm that β-arrestin-1-biased signalling was capable of activating TRPC3 function *in vitro*, we measured the effects of TRV120027 on [Ca^2+^]_i_ and membrane conductance in HEK293 cells heterologously expressing AT1R, β-arrestin-1 and TRPC3. As a result, TRV120027 induced a [Ca^2+^]_i_ increase in HEK293 cells co-transfected with AT1R, β-arrestin-1 and TRPC3, which were significantly blocked by Pyr3 pre-incubation (Fig. 5h,i). In contrast, TRV120027 could not induce either an increase in [Ca^2+^]_i_ or a significant inward current in the HEK293 cells co-transfected with only AT1R and the β-arrestin-1 plasmids or with TRPC3 and the β-arrestin-1 plasmids (Supplementary Figs 21–24). Although Pyr3 blocked a significant fraction of the AngII-induced [Ca^2+^]_i_ increase, it had no significant effect on TRV120055-induced calcium elevation in HEK293 cells co-transfected with AT1R, β-arrestin-1 and TRPC3 (Supplementary Fig. 23a–c). Moreover, whereas I–V analysis of TRV120027 indicated that the agonist significantly increased the amplitude and slope of current responses of TRPC3, this effect was abolished by Pyr3 treatment, indicating the opening of TRPC3 channels by β-arrestin-1-biased AT1R signalling in this recombinant system (Fig. 5j,k).

### β-arrestin-1 scaffolding functional TRPC3 complexes

**Figure 6:**
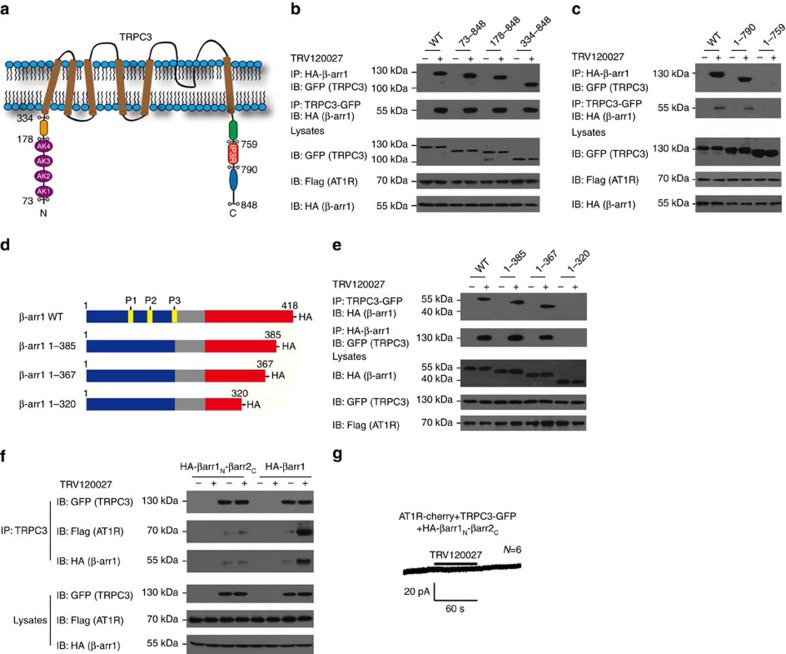
Molecular mechanism of agonist-induced TRPC3–β-arrestin-1 interaction. (**a**) Schematic representation of wild-type and different truncations of TRPC3. (**b**,**c**) Mapping of the β-arrestin-1-binding region in TRPC3. HEK293 cells co-transfected with AT1R-cherry, β-arrestin-1 and different truncations of TRPC3. The interaction was probed by co-immunoprecipitation. (**d**) Schematic representation of different β-arrestin-1 truncations and mutations. (**e**) Mapping of the TRPC3 binding region in β-arrestin-1. HEK293 cells co-transfected with AT1R-cherry, different β-arrestin-1 truncations and TRPC3-GFP. The interaction was probed by co-immunoprecipitation. (**f**) HEK293 cells co-transfected with AT1R-cherry, TRPC3-GFP and β-arrestin-1-WT or βarr1_N_-βarr2_C_. The interaction was detected by co-immunoprecipitation. (**g**) TRV120027 could not induce an apparent inward current in HEK293 cells co-transfected with AT1R-cherry, TRPC3-GFP and βarr1_N_-βarr2_C_. (**b**–**f**) A representative western blot from at least three independent experiments was shown.

The large cytoplasmic N- and C-terminal ends of TRPC3 constitute an expanded structure that is regulated by multiple stimuli. To map the specific region of TRPC3 binding to β-arrestin-1, we used a panel of N- and C-terminal TRPC3 deletions to co-immunoprecipitate haemagglutinin (HA)-tagged β-arrestin-1 after TRV120027 stimulation (Fig. 6a and Supplementary Fig. 25). Neither of the series of deletions of TRPC3 localized at the N terminal nor did the last 58 amino-acid C-terminal deletion affect the formation of the β-arrestin–1-TRPC3 complex after TRV120027 administration (Fig. 6b,c and Supplementary Figs 25 and 26). However, a further 31 amino-acid truncation at the C terminal of TRPC3 (1–759) abolished the interaction between β-arrestin-1 and TRPC3 (Fig. 6c and Supplementary Figs 25 and 26). These results indicated that the residues between 759 and 790 of TRPC3, which correspond to the previously identified calmodulin (CaM) or IP3 receptor (IP3R)-binding motif, are a key signature for β-arrestin-1 interaction (Fig. 6a)^29^.

We next used a similar approach to map the critical regions of β-arrestin-1 that are required for TRPC3 association (Fig. 6d). Whereas truncation of the C-terminal 45 residues did not significantly decrease the TRV120027-induced β-arrestin-1–TRPC3 interaction, a further deletion of the C-terminal 47 residues in β-arrestin-1 impaired the association (Fig. 6e and Supplementary Figs 25 and 26). Therefore, the critical element involved in the TRPC3 interaction was the C-terminal 320–367 region of β-arrestin-1, which included the previous known ‘splice loop' involved in arrestin and clathrin interactions. We noticed that the electrophysiological results indicated that only β-arrestin-1, but not β-arrestin-2, is required for TRV120027-induced acute catecholamine secretion, suggesting a potential subtype specificity of β-arrestin functions. Interestingly, the sequence of the C-terminal regions of the β-arrestin-1 and β-arrestin-2 is not conserved, hallmarked by a lack of the ‘splice loop' in the C terminal of β-arrestin-2 that may be involved in the TRPC3 interaction (Supplementary Fig. 27a). We therefore constructed a chimera protein (HA-βarr1_N_-βarr2_C_) that linked the N terminal of β-arrestin-1 (1–320) to the C-terminal of β-arrestin-2 (321–409) and tested its interaction with TRPC3 and its function in TRV120027 potentiated TRPC3 activity (Supplementary Fig. 27b). As shown in Fig. 6f and in Supplementary Figs 28 and 29, although the application of TRV120027 significantly increased the association between TRPC3-GFP (green fluorescent protein) and HA-β-arrestin-1 or Flag-AT1aR in cells co-transfected with TRPC3-GFP, HA-β-arrestin-1 wild-type and Flag-AT1aR, this potentiation was not observed in cells co-transfected with TRPC3-GFP, HA-βarr1_N_-βarr2_C_ and Flag-AT1aR. Consistently, TRV120027 could not induce TRPC3 current in HEK293 cells co-transfected with TRPC3-GFP, HA-βarr1_N_-βarr2_C_ and Flag-AT1aR (Fig. 6g). We next constructed a chimera protein (HA-βarr2_N_-βarr1_C_) that linked the N terminal of β-arrestin-2 (1–321) to the C-terminal of β-arrestin-1 (320–418) (Supplementary Fig. 27c). TRV120027 induced HA-βarr2_N_-βarr1_C_ and TRPC3 association (Supplementary Fig. 30). Taken together, these results demonstrated that the C-terminal region of β-arrestin-1 mediates its interaction with TRPC3 and is required for TRV120027-induced TRPC3 current, which is a determinant for the functional diversity of the β-arrestin subtype in adrenal medulla.

**Figure 7:**
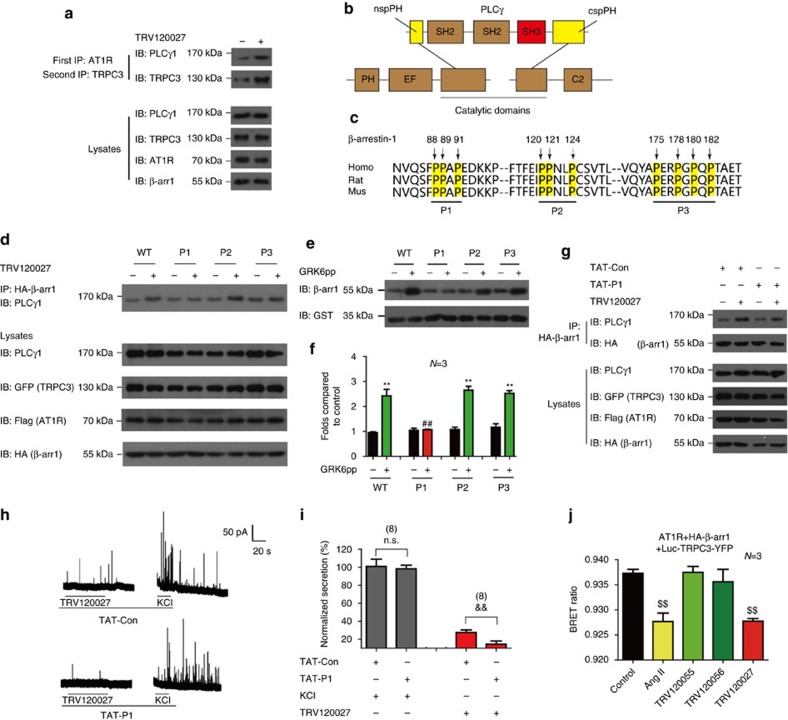
Interaction of the PLCγ1 with the AT1R–β-arrestin-1 complex. (**a**) Sequential co-immunoprecipitation (Co-IP) experiments were performed as described in Fig. 5g. PLCγ1 recruit to the AT1R–TRPC3 complex after stimulation. (**b**) Domain organization of the PLCγ1. The SH3 domain was shown in red. (**c**) Sequence alignment of the residues constituting different poly-proline sites of β-arrestin-1 across different species. (**d**) Effects of TRV120027 on interactions between PLCγ and β-arrestin-1 WT or different mutants. (**e**) The GST-PLCγ-SH3 (5 μM) domain was incubated with 5 μM β-arrestin-1 WT or different mutants in the presence (or not) of GRK6-phosphorylated β2-adrenergic receptor C-tail (GRK6pp) *in vitro*; the association was examined by GST pull down assay. (**f**) Bar graph and quantification statistics of **e**. (**g**) HEK293 cells co-transfected with AT1R-cherry, TRPC3-GFP and β-arrestin-1 were pre-incubated with 5 μM TAT-con or TAT-P1 peptide for 2 h and were then stimulated with TRV120027. The association between PLCγ and β-arrestin-1 was detected. (**h**) High potassium chloride or TRV120027 (100 nM) induced amperometric currents in primary chromaffin cells pre-incubated with 5 μM TAT-con or TAT-P1 peptides. (**i**) Summary bar graph of acute catecholamine secretion in Fig. 6h. (**j**) HEK293 cells co-transfected with AT1R, β-arrestin-1 and Luc-TRPC3-YFP and conformational changes were measured by intramolecular BRET. (**f**). **P<0.01; GRK6pp incubation was compared with no stimulation. ^##^*P*<0.01, β-arrestin-1 mutant was compared with wild type. (**i**) ^&&^*P*<0.01; TAT-P1 peptide incubation was compared with TAT-con peptide. (**j**) ^$$^*P*<0.01; different agonist treatments were compared with control vehicles. (**a**,**d**–**g**) A representative western blot from at least three independent experiments was shown. The bars represent mean±s.d. All data statistics were analysed using one-way ANOVA.

Previous studies have determined that PLCγ is required for proper plasma membrane localization of TRPC3 and is proposed to be involved in TRPC3 activation through conformational change^30^,^31^. Our sequential co-immunoprecipitation experiments demonstrated that TRV120027 promoted the recruitment of the PLC-γ to the complex of AT1R–TRPC3 or AT1R–β-arrestin-1 (Fig. 7a and Supplementary Figs 31–34). More importantly, the TRV120027-induced β-arrestin-1–PLCγ complex formation required the poly-proline region 1 but was not affected by mutations in the poly-proline region 2 or 3 of the β-arrestin-1 (Fig. 7b–d and Supplementary Figs 35 and 36). We then used the V2 receptor phospho-C-tail to activate β–arrestin-1 and examined the interaction between the active β-arrestin-1 and the SH3 domain of PLCγ *in vitro*^26^,^27^. A direct interaction between the poly-proline region 1 of β-arrestin-1 and the SH3 domain of PLCγ was also demonstrated *in vitro* by glutathione S-transferase (GST)-pull down assay (Fig. 7c,e–f). Moreover, we made the peptide TAT-P1 by conjugating the N-terminal-HIV-TAT tag to the poly-proline region 1 (amino acids from 82 to 94) of β-arrestin-1 (Supplementary Fig. 37a). Fusing the peptide to the N-terminal-HIV-TAT enabled the peptide to traverse the cell membrane. As shown in Fig. 7g, the application of the TAT-P1 specifically disrupted the association between β-arrestin-1 with PLC-γ induced by TRV120027, whereas a control peptide TAT-con had no significant effect (Fig. 7g and Supplementary Fig. 37b). Moreover, the incubation of the peptide TAT-P1 with the primary chromaffin cells derived from wild-type mice significantly blocked TRV120027-induced catecholamine secretion, whereas this had no effect on high KCl-induced secretion (Fig. 7h,i). These results suggested that the specific interaction between the poly-proline region 1 of β-arrestin-1 and the SH3 domain of PLCγ underlies the β-arrestin-biased agonism-induced functional TRPC3 complex formation and catecholamine secretion from primary chromaffin cells.

Finally, we generated a BRET sensor (Rluc-TRPC3-YFP) to monitor the conformational change of TRPC3 after activation of AT1R induced by different agonists. Although all AT1R-biased agonists stimulated catecholamine secretion as detected by amperometric current traces (Fig. 1a–f), only AngII and TRV120027 induced significant decrease in the intramolecular BRET signal of TRPC3, whereas Gq-biased agonist TRV120055 and TRV120056 did not(Fig. 7j). The agonist-induced BRET signals were not significantly affected by the application of the PLC-β inhibitor U73122, and no BRET signals were detected without the co-transfection of β-arrestin-1 (Supplementary Fig. 38a,b). These result suggested that β-arrestin-1-mediated AT1R signalling caused specific TRPC3 conformational change. Further, the decrease of the BRET signal between N terminal and C terminal of TRPC3 suggested that the two terminals of the TRPC3 were more separated because of the β-arrestin-1 association, which may be required for β-arrestin-1-mediated TRPC3 activation. Taken together, these results indicate that the β-arrestin-1-biased agonism promoted a functional macromolecular TRPC3 complex formation, in which β-arrestin-1 recruited both TRPC3 and PLCγ through specific interactions that enabled specific conformational change and activation of TRPC3.

### A general role of the GPCR–β-arrestin-1–TRPC3 pathway

**Figure 8:**
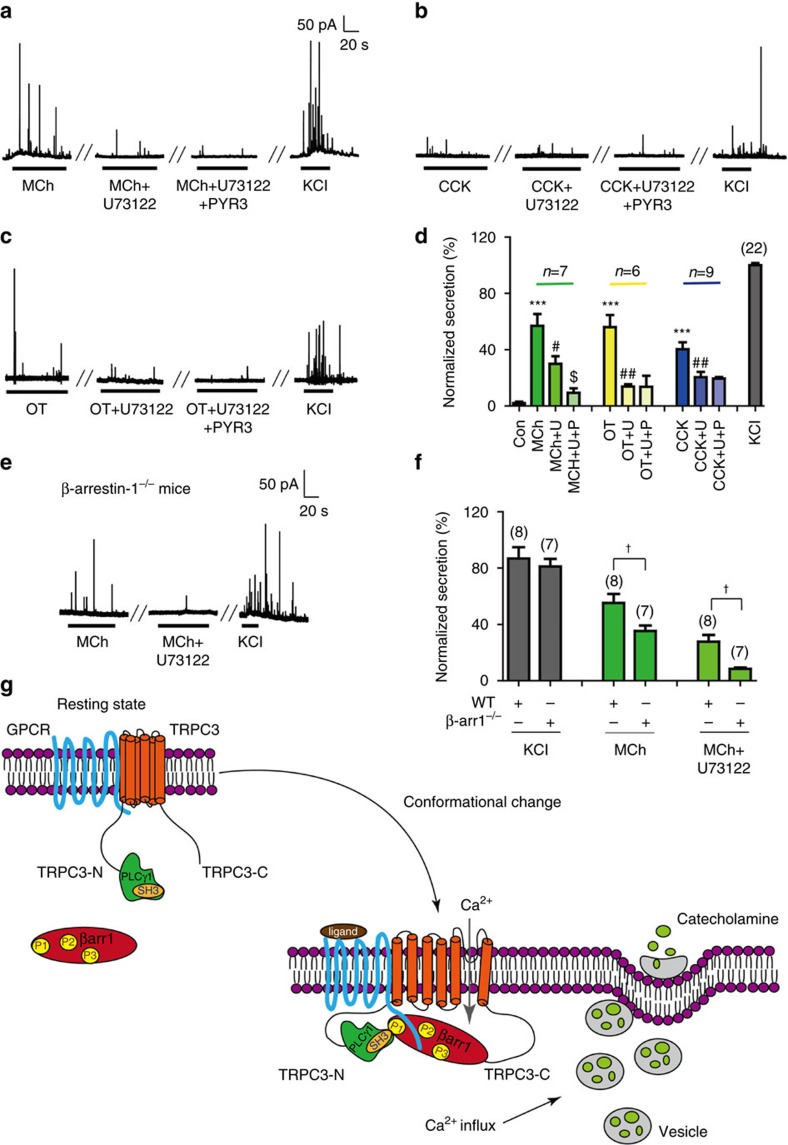
A general role of β-arrestin–1-TRPC3 pathway in agonist-induced secretion. (**a**–**c**) Effects of the PLC inhibitor U73122 (2 μM) and the TRPC3 inhibitor Pyr3 (10 μM) on different agonist-induced catecholamine secretions of primary chromaffin cells. (**a**) Mch (100 μM); (**b**) CCK-8 s(1 nM); (**c**); OT (10 nM). (**d**) Statistical analysis and bar graph representation of the agonist-induced catecholamine secretions in **a**–**c**. (**e**) Mch (100 μM) induced catecholamine secretion of the primary chromaffin cells from the *β-arrestin-1*^*−/−*^ mice with or without U73122 application. (**f**) Statistical analysis and bar graph representation of the agonist-induced catecholamine secretion in *β-arrestin-1*^*−/−*^mice and their wild-type littermates. (**d** or **f**) ***P<0.005; agonists stimulated cells were compared with non-stimulated cells. ^#^*P*<0.05; ^##^*P*<0.01; U73122-treated cells were compared with control vehicles. ^$^*P*<0.05; chromaffin cells pre-incubated with Pyr3 were compared with control vehicles. ^†^*P*<0.05, *β-arrestin-1*^*−/−*^ mice were compared with WT mice. The bars represent mean±s.d. All data statistics were analysed using one-way ANOVA. (**g**) A schematic model of arrestin-mediated (seven transmembrane receptor) 7TMR-TRPC3 coupling and hormone secretion. For at least a subset of GPCRs, such as AT1R and mAchR, there are constitutive interactions between receptor and TRPC3 or TRPC3 and PLCγ in cells in a rest state. TRPC3 is inactive and β-arrestin-1 is in the cytoplasm at this stage. Upon agonist stimulation, β-arrestin-1 is recruited to the receptor, which interacts with the TRPC3 C terminal with its C-terminal region and interacts with the SH3 domain of PLCγ through its poly-proline P1 region. The interaction of β-arrestin-1 with PLCγ and TRPC3 enables TRPC3 conformational change and activates TRPC3, which induces extracellular calcium influx and catecholamine secretion.

Finally, to examine whether the β-arrestin-1–TRPC3 pathway underlies catecholamine secretion after activation of other GPCRs, we treated primary chromaffin cells with acetyl-β-Mch, cholecystokinin-8s (CCK-8s) and oxytocin (OT), which are known to activate the Mch receptor (mAchR), the cholecystokinin receptor and the OT receptor, respectively (Fig. 8a–c). Application of the PLC inhibitor U73122 blocked the amperometric spikes by 80% and 50% for primary chromaffin cells stimulated with OT and CCK-8s, respectively (Fig. 8b–d). Pre-incubation of chromaffin cells with Pyr3 had no significant effect on either OT- or CCK-8s-induced spikes after U73122 application, indicating that TRPC3 was not involved in Gq-PLCβ-independent catecholamine secretion after activation of either the cholecystokinin receptor or OT receptor (Fig. 8b–d). However, whereas U73122 reduced Mch-induced amperometric spikes by 50%, the combination usage of Pyr3 and U73122 further reduced Mch-induced catecholamine secretion to ∼15% (Fig. 8a,d), suggesting that TRPC3 activation also underlies mAchR-mediated secretion in chromaffin cells. Consistently, Mch-induced catecholamine secretion was significantly reduced in *β-arrestin-1*^*−/−*^ mice compared with wild-type mice. Application of U73122 significantly reduced Mch-induced catecholamine secretion by ∼40% in wild-type mice, whereas it decreased Mch-induced catecholamine secretion by almost 80% in *β-arrestin-1*^*−/−*^ mice (Fig. 8e,f). Therefore, the β-arrestin-1-TRPC3 pathway is an active component in mediating Mch-induced catecholamine secretion in primary chromaffin cells. Taken together, these results suggest that the β-arrestin-1-TRPC3 pathway underlies the acute catecholamine secretion downstream of a distinct subset of seven transmembrane receptors.

## Discussion

The adrenal gland is an important endocrine organ that generates a wide variety of hormones to regulate many physiological responses (for example, inducing the fight–flight response by producing catecholamines)^32^. Inappropriate plasma levels of the key hormones produced by the adrenal glands, such as catecholamines, corticosteroids or mineralocorticoids, are correlated with human diseases, such as heart failure or Cushing's syndrome^33^,^34^. Hormone secretion by the adrenal gland is tightly regulated by various GPCRs. Although acute activation of the α_2_ adrenoceptors or the purinergic P2Y receptors decreased catecholamine secretion in adrenal medulla, the activation of β adrenoceptors, NPY receptors, mAchR and AT1R promoted epinephrine or norepinephrine secretions^35^,^36^. All of these GPCRs share common signalling pathways, including G proteins or β-arrestins. In contrast to the well-characterized G protein signalling pathway, arrestin functions in the adrenal gland have just begun to be appreciated. Recent studies have demonstrated that the β-arrestin-1-mediated pathway promotes aldosterone turnover, which is highly correlated to the processes of heart failure^33^. However, how β-arrestins participate in catecholamine secretion in the adrenal medulla is not understood. Using a series of AT1R-biased agonists and specific β-arrestin knockout mice, we demonstrated that β-arrestin-1-biased agonism downstream of AT1R activation-promoted acute catecholamine secretion. In addition to AT1R, further experiments revealed that the β-arrestin-1-TRPC3 signalling also contributed to the Mch-induced acute catecholamine secretion, which may mimic the physiological conditions, such as para-sympathetic nerve activation. Therefore, the catecholamine secretion regulated by β-arrestin-1 agonism may underlie functions of a subset of GPCRs in the adrenal gland.

Studies have also shown that β-arrestin-1-mediated AT1R signalling resulted in the promotion of aldosterone production and secretion in the adrenal cortex^33^,^37^, not just in the adrenal medulla. Although the exact distribution or localization of TRP channel subtypes in the adrenal cortex remains unclear, β-arrestin-1-TRPC3 signalling may also be involved in receptor regulated aldosterone secretion, and this is worthy of further investigation.

The newly developed TRV120027 is a peptide ligand that has therapeutic potential to treat acute heart failure. Application of TRV120027 reduced blood pressure and preserved cardiomyocyte contractility in both animal experiments and clinical studies^21^,^22^,^23^. Here the acute effects of TRV120027 in eliciting the catecholamine secretion were consistent with the effect of the ligand in cardiomyocyte contractility and may contribute to its beneficial effects clinically. However, in the long term, higher circulating plasma catecholamine or aldosterone levels are positively correlated to heart failure^33^,^38^. Recent studies have demonstrated that the overexpression of β-arrestin-1 in the adrenal gland resulted in the deterioration of ventricular function. β-arrestin-1 knockout mice showed decreased cardiac infract size and apoptosis, as well as circulating aldosterone and catecholamines after post-myocardial infraction^33^,^37^; these results are consistent with our observations. Therefore, both our study and previous studies regarding β-arrestin-1 functions in adrenocortical zona glomerulosa cells indicate that the long-term inhibition of β-arrestin-1 functions in the adrenal gland will be helpful in the treatment of heart failure under certain pathological conditions.

In particular, we showed that β-arrestin-1 and β-arrestin-2 function oppositely in TRV120027-stimulated calcium increases in primary chromaffin cells (Fig. 3e,g). Whereas the β-arrestin-1 knockout abolished TRV120027-induced calcium increases that are required for catecholamine secretion, the β-arrestin-2 knockout increased intracellular calcium after TRV120027 administration. The observed phenomenon of the distinct roles of β-arrestin subtypes in TRV120027-induced calcium increases adds to the increasingly long list of physiological effects in which these two isoforms oppose each other in specific cellular contexts^39^,^40^,^41^,^42^. To the best of our knowledge, most of the beneficial effects of TRV120027 in the heart and renin systems are mainly mediated by β-arrestin-2-mediated AT1R signalling^23^,^43^. It's also worth to note that biased agonists of opioid receptor has been characterized to have β-arrestin subtype signalling selectivity. For agonists of AT1R, whereas the TRV120027 displayed more β-arrestin-1-biased molecular efficacy within the equi-active model analysis, the TRV120026 exhibited more β-arrestin-2-biased molecular efficacy (Supplementary Fig. 39). Therefore, future studies to identify ligands that preserve the ability to activate β-arrestin-2-biased AT1R signalling but are devoid of Gq and β arrestin-1 signalling may result in more beneficial drug candidates for treating cardiovascular diseases.

More generally, our work identified a new mode of ion channel regulation by seven transmembrane receptors via β-arrestin (Fig. 8g). Fast communication between GPCRs and ion channels has advantages for cells to quickly respond to extracellular stimuli, which is of great interest in cell signalling studies. Activation of ion channels by GPCR through G protein pathways is well known^1^,^3^,^44^ (for example, Gq-PLC pathway or activation of certain types of potassium channels by β-gamma dimers). Recent studies have also identified direct activation of Kir7.1 by MC4R, and a paralleling paper just discovered Gi and PLC-δ-1-mediated TRPC4 activation^45^,^46^. Here we demonstrated that β-arrestin-1 promoted acute catecholamine secretion through coupling and activation of TRPC3. The β-arrestin-biased ligand TRV120027 induced the recruitment of both PLCγ and TRPC3 to the AT1R–β-arrestin-1 complex that enabled specific TRPC3 conformational changes. Whereas the β-arrestin-1 C terminal directly interacted with the CaM and IP3R-binding region of the TRPC3 C terminal, it also indirectly interacted with the N terminal of TRPC3 through recruiting PLCγ. Notably, previous studies have determined that the SH3 domain of PLCγ is required for agonist-induced extracellular calcium influx after intracellular calcium store depletion^30^,^47^. However, the role of the SH3 domain of PLCγ in mediating TRPC3 activation remained mysterious because the SH3 domain of PLCγ does not directly interact with TRPC3 (ref. 47). Here the recruitment of PLCγ to β-arrestin-1 through the direct interaction between the poly-proline region 1 of β-arrestin-1 and the SH3 domain of PLCγ may provide a key missing link in mediating TRPC3 activation (Fig. 7b–d and Fig. 8g). Moreover, recent studies have demonstrated that binding of the adaptor protein to the ‘CaM-IP3R'-binding region may regulate TRPC3 or TRPV1 activity^48^. Our latest research suggested that receptor activation induced various distinct arrestin conformations, which enabled the transduction of multiple signals through protein interactions^26^. Therefore, specific conformational changes may underlie TRPC3 activation by arrestin, which requires future structural or biophysical studies. In our BRET assays, we observed the β-arrestin-1-mediated signalling pathway specifically caused separation of the N- and C termini of TRPC3, potentially in a concatenated way through its N-terminal interaction with the PLCγ and its C-terminal interaction with the C terminal of TRPC3. A hypothetical interaction model was given in Fig. 8g.

Further, the arrestin-dependent TRP activation displayed strong TRP subtype specificity despite the presence of multiple TRP channels in primary chromaffin cells (Fig. 4). This result is in stark contrast to the canonical Gq-PLC pathway, which non-selectively activates many TRP channels. Depending on different physiological conditions, the activity and channel functions change drastically. Therefore, the specific linkage of AT1R to TRPC3 activity though β-arrestin-1 exemplified precise control of ion channel activity by seven transmembrane receptors via membrane-localized signalling domains.

Finally, our results provided direct evidence that β-arrestin mediated quick responses of GPCR functions at the plasma membrane other than the ‘second-wave' GPCR signalling in early endosomes. Agonists binding to GPCRs trigger a series of conformational changes, which are then turned into specific signalling through either G protein subtypes or arrestins. Arrestins first terminate G protein signalling through desensitization and then initiate their own signalling^13^,^14^,^15^. In contrast to the quick response of G protein activity, arrestin-mediated GPCR signalling, such as extracellular signal-regulated kinase activation or protein ubiquitination, is often slower, starting 2–3 min after ligand exposure^16^,^32^,^49^. In the traditional view, the slower response of the arrestin pathway is partially because of the required timeline for assembling the arrestin signalling complex in endosomes after receptor endocytosis. In recent studies, the application of the conformational selective biosensors to the β-adrenergic receptor system enabled the direct observation of the ‘three waves' of GPCR signalling, of which the first wave is initiated by the GPCR–G protein complex in the plasma membrane, the second wave is triggered by the GPCR–arrestin complex in endosomes and the third wave is initiated by the signalling of the GPCR–G protein complex in endosomes^13^,^15^. In such conventional thoughts, functions of arrestins are sequentially timed. Arrestins first terminate G protein signalling and then initiate G protein-independent pathways.

However, the subsidiary role of arrestin in first-wave receptor signalling is challenged by evolutionary analysis, in which the arrestins appear earlier in archaea and bacteria compared with the later appearance of the G proteins in eukaryotes^18^. Evolutionary analysis suggests an equal importance of arrestins and G proteins in receptor signalling. During our manuscript submission, a study shows that β-arrestin-2-mediated MAPK signalling could be initiated through clathrin-coated pits downstream of specific β1-adrenergic receptor signalling^26^,^50^. Here using the arrestin-biased agonist and different knockout models, the present study identified that the β-arrestin-1 agonism of GPCR allowed acute responses of extracellular hormone stimulation similar to G protein functions, in which the timeline was less than 30 s. β-arrestin agonism-inducing TRPC3 activity is mainly restricted to the plasma membrane (Fig. 5a). Therefore, the coupling of a single receptor to different downstream effects not only allows initiating signalling events in a temporal manner but also controls localized GPCR functions in microcellular domains. Taken together, the current results revise the conventional thinking of the working manner of arrestins after GPCR activation and introduce a new paradigm for GPCR signalling and arrestin functions.

In conclusion, our studies revealed a previously unrecognized role of β-arrestin subtype-biased agonism in acute hormone secretion, linking seven transmembrane receptors with the activation of a specific TRP channel. β-arrestin-1 is capable of recruiting both PLCγ and TRPC3 to receptor–β-arrestin-1 in order to assemble a functional complex that can mediate catecholamine secretion in chromaffin cells (Fig. 8g). The newly identified GPCR signalling mode will revise the current thinking on the arrestin functional paradigm and may have broad implications for the working mechanism of cell responses to extracellular stimuli in different physiological contexts.

## Methods

### Reagents

The anti-TRPC3/6/7 (sc-15058, 1/1,000), anti-GFP (sc-9996, 1/1,000), anti-AT1R (sc-1173, 1/1,000), anti-HA (sc-7392, 1/1,000), anti-PLCγ1 (sc-81, 1/1,000) antibodies were purchased from Santa Cruz. The anti-Flag (2368, 1/1,000), anti-GAPDH (5174, 1/1,000), anti-GST (2622, 1/1,000) antibodies were from Cell Signaling. The anti-HA-tag beads and anti-GFP-tag beads were from Medical & Biological Laboratories Co., Ltd. (Japan). The anti-Flag M2 beads (A2220) were from Sigma. The anti-β-arrestin-1 (A1CT, 1/5,000) and anti-β-arrestin-2 antibodies (A2CT, 1/2,000) were generous gifts from Dr R.J. Lefkowitz (Duke University). The pertussis toxin was from Enzo Life Sciences. The SNX482 was from Abcam. The LaCl_3_ and CdCl_2_ were purchased from Sangon Biotech (Shanghai) Co. The Fura-2-AM was from Invitrogen. The DMEM medium was from Thermo Scientific. All other chemical or reagents were purchased from Sigma.

### Constructs

Plasmids encoding β-arrestin-1, TRPC3 and AT1R were generous gifts from Dr R.J. Lefkowitz at Duke University (Supplementary Data 1). The SH3 domain of PLCγ was synthesized by DNA synthesis according to the human PLCγ sequence. The mutants of β-arrestin-1 (1–385, 1–367, 1–320, P88-89-91/3A, P121A, P180A) and TRPC3 (73–848, 178–848, 334–848, 1–790, 1–759) were generated by the Quikchange Mutagenesis Kit (Stratagene). All constructs were verified by DNA sequencing.

### Cell culture and transfection

Primary chromaffin cells derived from wild-type, β-arrestin, Gq or TRPC3 knockout mice, as well as HEK293 cells were maintained at 37 °C and 5% CO_2_ in DMEM medium containing 10% fetal bovine serum (FBS) and 1% penicillin/streptomycin. For electrophysiology and cellular experiments, cells were transfected with plasmids encoding different constructs of AT1R, β-arrestin-1 or TRPC3 using PEI (Polysciences) according to the manufacturer's instructions.

### Calcium measurements

[Ca^2+^]_i_ was measured as previously described^51^,^52^. The mouse adrenal chromaffin cells derived from WT, *β-arrestin-1*^*−/−*^ or *β-arrestin-2*^*−/−*^ mice were cultured for 2–4 days before the experiments. All experiments of calcium measurements using primary chromaffin or HEK293 cells were carried out at room temperature (22–25 °C). The primary cells derived from WT, *β-arrestin-1*^*−/−*^ or *β-arrestin-2*^*−/−*^ mice or HEK293 cells transfected with different plasmids were incubated in imaging buffer I (10 mM glucose,150 mM NaCl, 5 mM KCl, 1.3 mM MgCl_2_, 1.2 mM NaH_2_PO_4_, 3 mM CaCl2, 20 mM HEPES, pH 7.4). In the Ca^2+^-free bath, the 3 mM CaCl_2_ of the imaging buffer I was replaced by 5 mM EGTA. The change of [Ca^2+^]_i_ concentration was measured by an intracellular Ca^2+^ imaging system (TILL, Germany)^51^. Isolated mouse adrenal chromaffin cells from wild type or different knockout mice were incubated at 37 °C for 30 min in a solution containing 2 μM Fura-2/AM. We then used the F340/F380 ratio of Fura-2 intensity to monitor the intracellular calcium change challenged by a spectrum of AT1R agonists, such as Ang II (100 nM), SII (1 μM) and TRV120027 (100 nM). We measured the intracellular Ca^2+^ concentration in primary cells by dual-wavelength ratio-metric fluorometry. We excited the Fura-2 with alternative light between 340 and 380 nm by a monochromator-based system (TILL Photonics). The cooled charge-coupled device was used to measure the resulting fluorescence signals. We calculated the relative changes in [Ca^2+^]_i_ by the ratio of F340 to F380. The electrophysiology recording was analysed using the Igor software (WaveMetrix). The statistical analysis was performed with *t*-test or two-way analysis of variance (ANOVA).

### Current measurement in primary chromaffin cells

Adrenal chromaffin cells were isolated from 8-week-old wild-type female mice, and were cultured on coverslips used for patch-clamp experiments after 70–100 h incubation. We used the whole-cell patch-clamp to record the membrane currents of adrenal chromaffin cells derived from wild-type or different knockout mice. The extracellular buffer for these primary cell cultures contains 145 mM NaCl, 2.8 mM KCl, 10 mM HEPES, 1.0 mM MgCl_2_.6H_2_O, 2.0 mM CaCl_2_.2H_2_O, 10 mM glucose and pH 7.4 adjusted with NaOH. The K+ outward current was blocked by 50 mM Tetraethylammonium and the activation of the Na^+^ current was abolished by 100 nM tetrodotoxin. Electrode with a resistance of 3.9∼5.1 MΩ was filled with internal solution (145 mM CsCl, 8.0 mM Nacl, 1.0 mM Mgcl_2_.6H_2_O, 10 mM HEPES, 0.4 mM GTP and 2.0 mM Mg-ATP, which was adjusted to pH 7.3 with CsOH). We ruptured the primary cell membrane with suction when the high-resistance seal is greater than 1.5 gigohm. The leak currents of a single primary cell derived from wild-type mice or knockout mice that are greater than −30 pA were excluded from further analysis. We monitored the gap-free recording and some cell parameters, such as *C*_cell_ (cell capacitance), *R*_s_ (series resistance), *R*_m_ (membrane resistance), to ascertain the constancy of the patch. During whole-cell recording, we used the amplifier circuitry to minimize the capacity current, and the *R*_s_ was compensated by 80% (ref. 52). Inward current in a single primary mouse chromaffin cell was induced by TRV120027 when bathed in an extracellular solution with 1 μM tetrodotoxin and 1 μM 4-AP and voltage-clamped at −60 mV. TRV120027 (final concentration, 5 μM) was added in the bath to stimulate primary cells. TheTRPC3 inhibitor Pyr3 was used in the bath at a final concentration of 5 μM. We perfused the recording chamber with standard external solution under gravity at a rate of ∼12 ml h^−1^. We recorded the ionic current by the data acquisition system (2012, DigiData 1322A, Axon) and an amplifier machine (2012, Axopatch-200B, Axon, USA). We used the pClamp Version 9 software to control the command voltages. All experiments and recordings were performed at room temperature (28 °C). We use patch-clamp amplifier (2012, HEKA EPC10) and patchmaster software (HEKA, Lambr-echt/Pfalz, Germany). Current and voltage signals of primary cells derived from wild type or knockout mice were low-pass-filtered (DC to 10 KHz) and acquired at 20 KHz. Data, graphs and current traces were analysed with the Igor software package.

### Current measurement in HEK293 cells

Whole-cell current recording was performed 20–38 h after co-transfection with Flag-AT1R-cherry, HA-β-arrestin-1 and TRPC3-GFP. For the negative control, the cells were co-transfected with plasmids encoding TRPC3-GFP and HA-β-arrestin-1, or Flag-AT1R-cherry and HA-β-arrestin-1. We used thin-walled glass to make patch electrodes, and the resistance of the electrodes should be 4.0∼6.0 MΩ when filled by internal solution (140 mM CsCl, 3 mM Mg-ATP, 2 mM Mgcl_2_.6H_2_O, 3.0 mM EGTA, 10 mM HEPES and PH 7.2 adjusted with CsOH). The extracellular solution was: 136 mM NaCl, 5.5 mM KCl, 2 mM MgCl_2_.6H_2_O, 2 mM CaCl_2_.2H_2_O, 10 mM HEPES, 10 mM glucose and pH 7.4 adjusted with NaOH. Membrane potential was held at 0 mV. Currents were elicited followed a ramp protocol ranged from −100 to +100 mV, lasting 100 ms and repeated every 5 s. All experiments were performed at 37 °C.

### Sequential immunoprecipitation experiments

HEK293 cells (generally six 100-mm culture dishes for each) co-transfected with Flag-AT1R-cherry, HA-β-arrestin-1 and TRPC3-GFP were stimulated with TRV120027 (100 nM) or control vehicle for 1 min. The plasma membrane fractions were first isolated by centrifugation at 20,000*g* for 1 h and then washed with PBS. The protein complexes containing Flag-AT1R were immunoprecipitated by Anti-Flag M2 agarose and then eluted with 3*Flag peptide (final concentration, 100 μg ml^−1^). The complexes containing Flag-AT1R were then immunoprecipitated by anti-HA beads or anti-GFP beads. TRPC3 associated with Flag-AT1R-HA-β-arrestin-1 or PLCγ1 associated with the AT1R–TRPC3 was detected by a specific antibody.

### Western blotting

To examine endogenous TRPC3/6/7, β-arrestin-1 and β-arrestin-2 expression in different tissues, the fresh brain or muscle tissues were isolated from WT, *β-arrestin-1*^*−/−*^, *β-arrestin-2*^*−/−*^, *TRPC3*^*+/−*^*TRPC6*^*−/−*^*TRPC7*^*−/−*^ or *TRPC3*^*−/−*^*TRPC6*^*−/−*^*TRPC7*^*−/−*^ mice. We then mixed tissues using cold lysis buffer (50 mM Tris, pH 7.4, 10 mM Pyrophosphate, 150 mM NaCl, 1 mM NaF, 1 mM phenylmethyl sulphonyl fluoride (Phenylmethylsulfonyl fluoride), 1% Triton, 2 mM EDTA, 10% glycerol, 1% NP-40, 0.25% sodium deoxycholate, 1 mM sodium orthovanadate, 0.3 μM Aprotinin, 130 mΜ Bestqatin, 1 μM Leupeptin, 1 μM Repstatin and 5 mM iodoacetate) and then these tissues (brain or muscles) were dounced in a glass tube for 15 min. Next, the mixtures were centrifuged at 1,000*g* for 15 min to discard the unbroken tissues and we subjected the mixtures to end-to-end rotation at 4 °C for 20 min and spun at 14,000*g* at 4 °C for 30 min. The supernatant was collected, denatured in loading buffer and detected by western blot. We quantified the protein bands of western blots by using the Image J software (Bethesda, MD).

### Co-immunoprecipitation

Co-immunoprecipitation was performed as previously described^10^,^53^,^54^. HEK293 cells were co-transfected with Flag-AT1R-cherry, TRPC3-GFP (WT/Truncations) and HA-β-arrestin1 (WT/Truncations/Mutants). Forty-eight hours after transfection, the cells were starved for 12 h and then stimulated with Ang II (100 nM), TRV120055 (30 nM) or TRV120027 (100 nM) for 1 min. Subsequently, the cells were washed with cold PBS and then collected in cold lysis buffer. The cell lysates were subjected to immunoprecipitation using different antibody-conjugated beads (Flag or HA-conjugated beads), which were incubated overnight at 4 °C. Immune complexes containing AT1R or arrestin were extensively washed for at least five times with cold lysis buffer and analysed by western blotting with specific antibodies.

### BRET assay

BRET assays were performed as previously described^28^. For the measurement of β-arrestin-1 recruitment to the plasma membrane, HEK293 cells were co-transfected with plasmids encoding Flag-AT1R, Luc-β-arrestin-1, TRPC3 and Lyn-YFP. For the intermolecular BRET, HEK293 cells were co-transfected with plasmids encoding Flag-AT1R, Luc-β-arrestin-1 or Luc-β-arrestin-2 and TRPC3-YFP or TRPV1-YFP/TRPC6-YFP. For the intramolecular BRET, HEK293 cells were co-transfected with plasmids encoding Flag-AT1R, HA-β-arrestin-1 and Luc-TRPC3-YFP plasmids for 48 h. After 12 h of starvation, cells were harvested and washed at least three times with PBS, and then cells were stimulated with AngII (100 nM), TRV120027 (100 nM) or other AT1R agonists for 1 min at 37 °C. Subsequently, we incubated the transfected cells with Coelenterazine h at room temperature (Promega S2011, final concentration, 5 μM) and two different light emissions were used for measurement (480/20 nm for luciferase and 530/20 nm for yellow fluorescent protein). All the BRET measurements were performed by a plate reader Mithras LB 940 (2013, Berthold Technologies) and the signal was determined by calculating the ratio of the light intensity emitted by yellow fluorescent protein over the intensity emitted by luciferase.

### Confocal microscopy

Confocal microscopy was performed as previously described^26^,^55^. HEK293 cells were co-transfected with plasmids encoding HA-β-arrestin-1, TRPC3-GFP and Flag-AT1R-cherry. The day following transfection, the cells were seeded on fibronectin-coated glass-bottom, 35-mm, at the density of 3 × 10^5^ cells per dish. The next day, the cells were starved and then stimulated with Ang II (100 nM) or TRV120027 (100 nM) for 1 min at 37 °C. Samples were then analysed using LSM 780 (Zeiss) laser-scanning confocal microscope.

### ELISA assay

ELISA assay was performed as previously described^10^,^11^. For epinephrine and norepinephrine secretion measurement in mouse adrenal medullas, freshly adrenal medullas were isolated from adult female mice (6–8 weeks) and cultured in DMEM medium containing 10% FBS and 1% penicillin/streptomycin. After 2 h of starvation, adrenal medullas were stimulated with different AT1R agonists for 1 min or 30 min at 37 °C. The supernatants were collected, and the epinephrine or norepinephrine secretion was measured by epinephrine or norepinephrine ELISA kit (Merck Millipore) according to the manufacturer's instructions.

### Protein expression and purification

The expression and purification of His-β-arrestin-1-WT or different Mutants were performed as previously described^53^. For the expression and purification of GST-PLCγ-SH3 protein, the plasmid encoding GST-PLCγ-SH3 was transformed into BL21 *Escherichia coli* and then the GST-PLCγ-SH3-transformed *E. coli* was cultured with 6 l LB (Luria Bertani) medium, and then we induced the *E. coli* with 0.3 mM isopropyl-b-D-thiogalactoside at 25 °C for 16 h and pelleted by centrifugation at 3,000*g*. We resuspended the cell pellets in GST incubation buffer (25 mM Tris-HCl pH 8.0, 150 mM NaCl, 5% glycerol, 0.5% Triton X-100, 2 mM EDTA and 1 mM dithiothreitol (DTT)) and broken by French press, followed with centrifugation at 13,500*g* for 1 h at 4 °C. We collected the supernatant and incubated it with 2 ml of glutathione-Sepharose 4B at 4 °C for 2 h. The beads were washed extensively with GST incubation buffer, and we eluted the bound GST-PLCγ-SH3 protein for GST pull down experiments by using 10 mM GSH (glutathione).

### GST pull-down assays

Binding of GST-PLCγ-SH3 to β-arrestin-1 was performed as previously described^26^. In detail, 10 μM purified β-arrestin-1-WT or different poly-proline mutant proteins were first mixed with equal molar of GRK6-phospho-β2AR peptides and incubated at room temperature in binding buffer containing 20 mM Tris-HCl, pH 8.0, 150 mM NaCl, 2 mM EDTA, 1 mM DL-DTT for 30 min. Subsequently, 10 μM purified GST-PLCγ-SH3 protein was added, and we incubated the mixtures at room temperature for another 1 h. Then, we added 10 μl GST-agarose to the mixture, and we subjected the mixtures to end-to-end rotation at 4 °C for 2 h. We collected the GST beads and washed them five times with cold binding buffer. After the final wash, we removed the supernatant and resuspended the samples in 2 × SDS loading buffer and the samples were boiled for 10 min. The arrestin–PLCγ–SH3 complexes were detected by using western blot with specific antibodies.

### Animals and primary chromaffin cell preparation

All animal care, usage and experiments were reviewed and approved by the Animal Use Committee of Shandong University School of Medicine. *β-arrestin-1*^*−/−*^(*Arrb1*^*−/−*^) and *β-arrestin-2*^*−/−*^ (*Arrb2*^*−/−*^) mice were obtained from Dr R.J. Lefkowitz (Duke University, Durham, NC, USA) and G. Pei (Tongji University, Shanghai, China); *TRPC3*^*−/−*^ and *TRPC6*^*−/−*^ mice were purchased from the Jackson Laboratory (ME, USA) and also were maintained and crossed as described previously^56^,^57^. *Gq*^*−/−*^ mice were obtained from Dr J.L. Liu at Shanghai Jiao Tong University and from C.S. Du at Tongji University. Methods for primary chromaffin cell isolation and maintenance were adapted by adjustment to previous studies^58^. All primary chromaffin cells were prepared from female mice. Two to four adrenal glands were collected following decapitation of 6–8 W C57B/L mice (Removal of cervical vertebra) and transferred to cold sterile D-Hank's solution. The isolated adrenal glands were decapsulated, trimmed and placed in cold fresh D-Hank's solution for removal of remaining cortical tissue. The use of microscopic instruments forceps can help in quickly removing the adrenal gland. The adrenal glands were removed and placed in D-Hank's. Pieces of adrenal medulla were then resuspended in enzymatic solution (Papain solution) and transferred to Eppendorf tube for ∼40 min digestion at 37 °C. To facilitate dispersion, cells were mechanically disrupted every 20 min on the flip. The enzyme solution was removed by sucking, followed with adding D-Hank's solution to stop the digestion, which were repeated for three times. Then 400∼600 μl D-Hank's solution was added to Eppendorf tube, with gentle pipetting until chromaffin cells were visible under the microscope. Then the supernatant was transferred to sheets, following with repeated pipetting until all precipitates disappear. Then the sheets were transferred to the incubator for 30 min at 37 °C. The supernatant was discarded and the cells were resuspended with 2 ml of cell culture medium. Mouse adrenal medulla chromaffin cells were cultured in DMEM medium containing 10% FBS, penicillin (100 UI ml^−1^) and streptomycin (100 μg ml^−1^) for at least 24 h before subsequent experiments. A mouse cell volume can be covered with three coverslips around the proposed 48 h to complete the relevant experimental operation.

### Electrochemical amperometry

We performed electrochemical amperometry using an Multiclamp 700B amplifier (2012, Axon, Molecular Devices, USA) interfaced to Digidata 1440A with the pClamp 10.2 software (Molecular Devices)^3^. All experiments were performed at room temperature (20–25 °C). Highly sensitive, low-noise, 5-μm CFEs (ProCFE, Dagan) were mildly positioned to touch a single mouse adrenal mouse adrenal medulla chromaffin cell to monitor the quantal release of the hormone containing Catecholamine substances. The holding potential was set at 780 mV and the Catecholamine substances released by chromaffin cells were detected by current changes. The standard external solution for our amperometry measurement is as follows: 10 mM HEPES pH 7.4, 10 mM glucose, 150 mM NaCl, 5 mM KCl, 2 mM CaCl_2_ and 2 mM MgCl_2_. Only events >5 s.d. were incorporated to analyse the kinetic properties of each amperometric spike. We analysed all data using Igor (WaveMetrix, Lake Oswego, Oregon) and a custom-made macro programme. Statistical data were given as the mean±s.d and analysed with *t*-test or two-way ANOVA.

### Statistics

All data are presented as mean±s.d. Differences between different groups were accessing using one-way ANOVA or two-way ANOVA in the GraphPad Prism 5 software.

### Data availability

The authors declare that data supporting the findings of this study are available within the paper and its Supplementary Information files and from the corresponding author upon reasonable request.

## Additional information

**How to cite this article:** Liu, C.-H. *et al*. Arrestin-biased AT1R agonism induces acute catecholamine secretion through TRPC3 coupling. *Nat. Commun.* **8,** 14335 doi: 10.1038/ncomms14335 (2017).

**Publisher's note:** Springer Nature remains neutral with regard to jurisdictional claims in published maps and institutional affiliations.

## Supplementary Material

Supplementary Information Supplementary Figures, Supplementary Tables.

Supplementary Data 1 Nucleotide sequences of plasmids. Nucleotide sequences of cDNAs of HA-βarr1-WT, HA-βarr1 1-320, HA-βarr1 1-367, HA-βarr1 1-385, βarr1-RFP, Luc-βarr1, Luc-βarr2, HA-βarr1N-βarr2C, HA-βarr2N-βarr1C, TRPC3-GFP-WT, TRPC3-GFP 73-848, TRPC3-GFP 178-848, TRPC3-GFP 334-848, TRPC3-GFP 1-790, TRPC3-GFP 1-759, TRPC3-YFP, Luc-TRPC3-YFP, Flag-AT1R-cherry, Flag-AT1R, Lyn-YFP

## Figures and Tables

**Table 1:** Summary of pharmacological properties of AT1R ligands.

**Ligand**	**G protein activation**	**β-arrestin-1 recruitment**	**β-arrestin-2 recruitment**
AngII (100 nM)	**+++ +**	**+++ +**	**+++ +**
TRV120055 (30 nM)	**+++ +**	**−**	**−**
TRV120056 (50 nM)	**+++**	**−**	**−**
S II (1 μM)	**−**	**+ +**	**+++**
TRV120026 (500 nM)	**−**	**+++ +**	**+++**
TRV120027 (100 nM)	**−**	**+++**	**+++ +**
Candesartan (100 nM)	**−**	**−**	**−**

AngII, angiotensin II; AT1R, angiotensin II receptor type 1.

Qualitative functional properties of AT1R ligands are expressed relative to the maximal stimulations of the cell in response to endogenous ligand AngII (++++). Amino-acid sequence, estimation of ligand activity and further description can be found in Supplementary Fig.1 and Supplementary Tables 1 and 2.
