# Supplementary material for: Arrestin-biased AT1R agonism induces acute catecholamine secretion through TRPC3 coupling
Source: Nat Commun. 2017 Feb 9;8:14335. doi: 10.1038/ncomms14335 (PMC5309860; doi:10.1038/ncomms14335)
Supplement: Supplementary Information — Supplementary Figures, Supplementary Tables. [file ncomms14335-s1.pdf]

Supplementary Figure 1

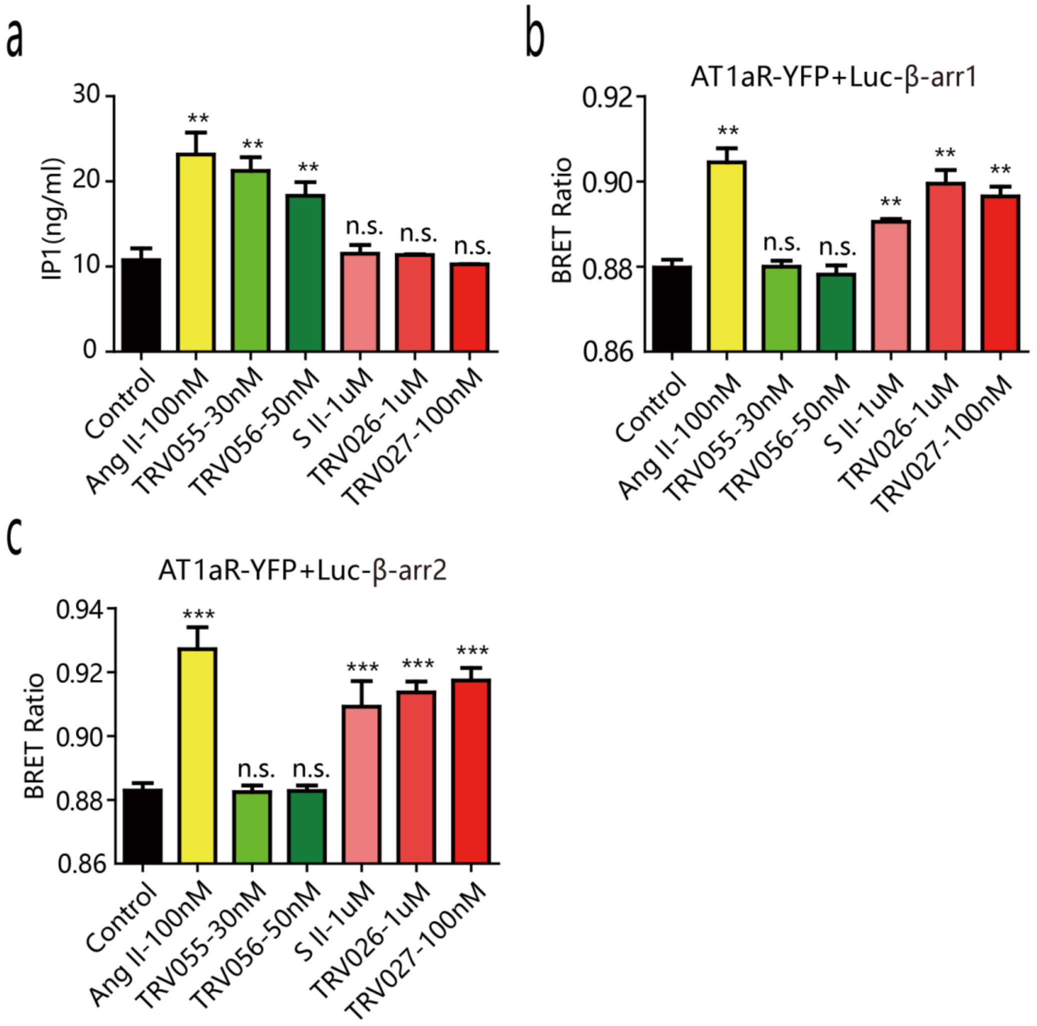

Supplementary Figure 1 In vivo quantification of ligand effect at Gq or β-arrestin signaling pathways.

a. Bar graph representation of IP1 production induced by different AT1R ligands. HEK293 cells were transfected with AT1R-YFP. After starvation, the cells were stimulated with different AT1aR ligands and the IP1 concentration was measured with an Elisa Kit.

b. Bar graph of β-arrestin-1 recruitment induced by different AT1R ligands. HEK293 cells were co-transfected with AT1R-YFP and Luc-β-arrestin-1. After starvation, the cells were stimulated with different AT1R ligands for 10 min and the interaction between the receptor and the β-arrestin-1 were measured using BRET.

c. Summary bar graph of β-arrestin-2 recruitment induced by different AT1R ligands. HEK293 cells were co-transfected with AT1R-YFP and Luc-β-arrestin-2. After

starvation, the cells were stimulated with different AT1aR ligand for 10 min and the interaction between the receptor and the β-arrestin-2 were measured using BRET.

a-c) **, $p<0.01$; ***, $p<0.005$; different AT1R agonist treatments were compared with control vehicles. ns, no significant difference. The bars represent mean±s.d of three independent experiments and the data were analyzed using one-way ANOVA.

Supplementary Figure 2

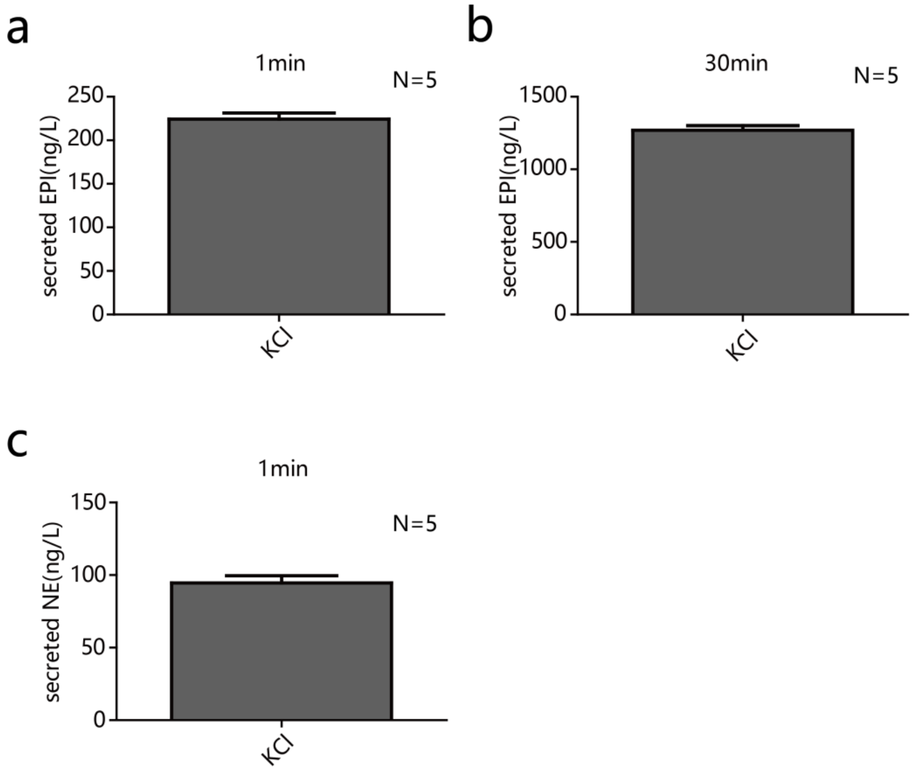

Supplementary Figure 2 High potassium chloride-induced epinephrine or norepinephrine secretion from the adrenal medulla examined using Elisa and correlated to Fig. 1i-k.

a. KCl-induced epinephrine secretion in the adrenal medulla, measured using an Elisa Kit at 1 min.
b. KCl-induced epinephrine secretion in the adrenal medulla, measured using an Elisa Kit at 30 min.
c. KCl-induced norepinephrine secretion in the adrenal medulla, measured using an Elisa Kit at 1 min. The bars represent mean±s.d and the data were analyzed using one-way ANOVA.

Supplementary Figure 3

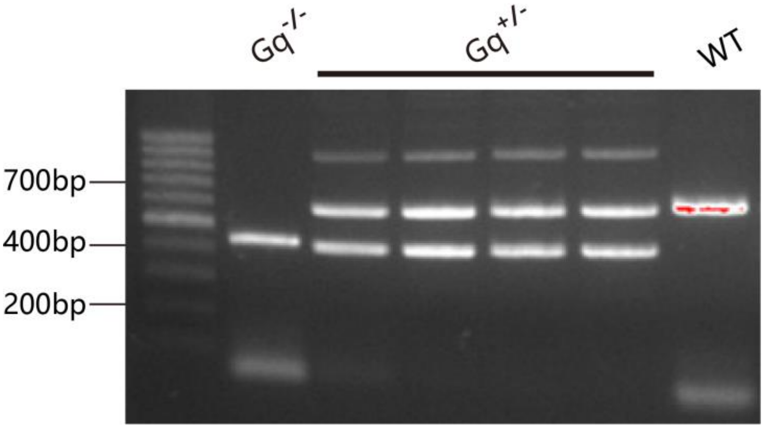

Supplementary Figure 3 Genotyping result of the Gq$^{-/-}$ mice

The PCR of the Gq$^{-/-}$ mice generates a product at 480 bp, the PCR of the wild-type mice generates a product at approximately 700 bp, and the PCR of the Gq$^{+/-}$ mice generates products at both 480 bp and ~700 bp.

Supplementary Figure 4

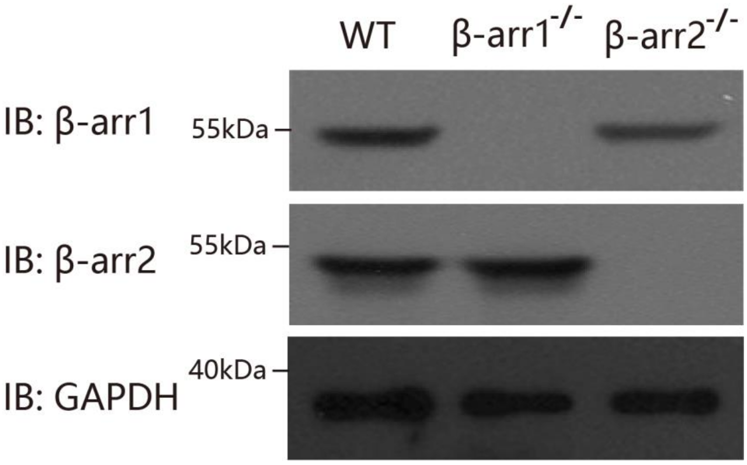

Supplementary Figure 4 Western results shows deficiency of β-arrestins in β-arrestin knock out mice.

Western blot of β-arrestin-1/2 in wild type， β-arrestin-1$^{-/-}$ or β-arrestin-2$^{-/-}$ mice.

Supplementary Figure 5

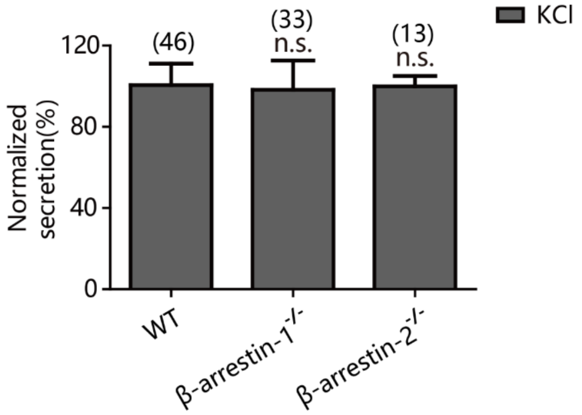

Supplementary Figure 5 Statistical analysis of the high potassium chloride-induced catecholamine secretion of the primary chromaffin cells derived from the β-arrestin-1$^{-/-}$ mice, the β-arrestin-2$^{-/-}$ mice or their wild type littermates.

The secretion was measured by the integration of the amperometric current traces after patch clamp measurements. ns, no significant difference between the β-arrestin-1$^{-/-}$ mice and their wild type littermates. The bars represent mean±s.d and the data were analyzed using one-way ANOVA.

Supplementary Figure 6

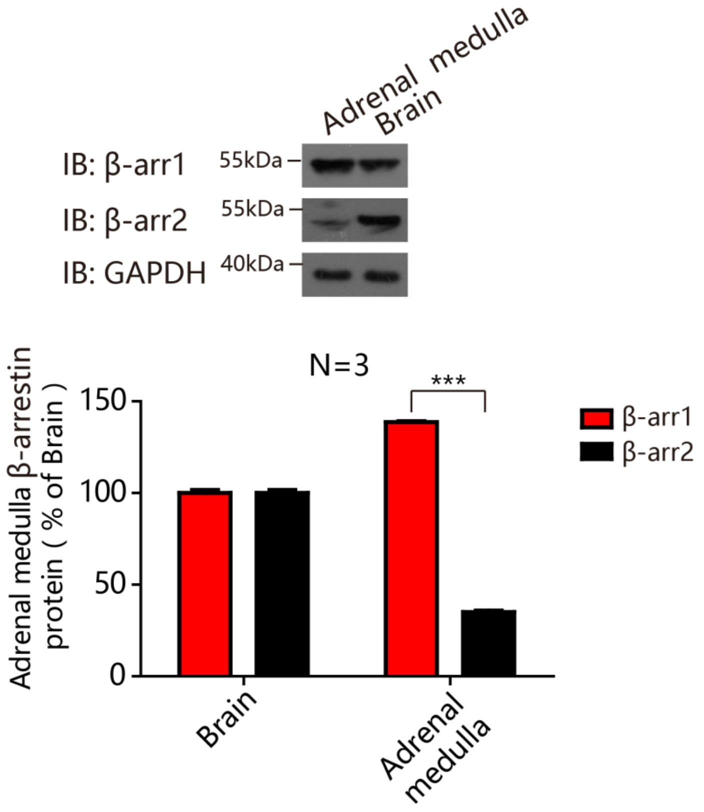

Supplementary Figure 6 Expression levels of β-arrestin-1 and β-arrestin-2 in adrenal medulla and brain examined by western blot. Top**,** representative blots; bottom, bar graph and quantification statistics. ***, $p<0.005$ (one-way ANOVA); β-arrestin-1 were compared with β-arrestin-2 in adrenal medulla. The bars represent mean±s.d.

Supplementary Figure 7

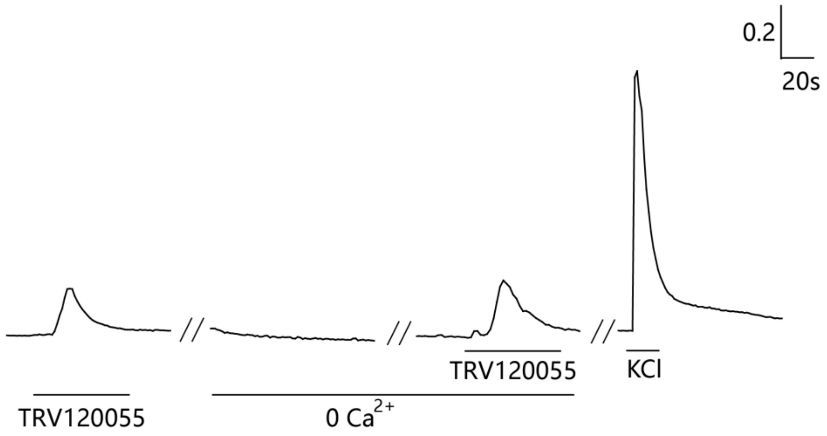

Supplementary Figure 7 TRV120055 (30 nM)-induced [Ca$^{2±}$]$_i$ elevation in chromaffin cells was not affected by a Ca$^{2±}$-deficient bath (related to Fig. 3k).

Supplementary Figure 8

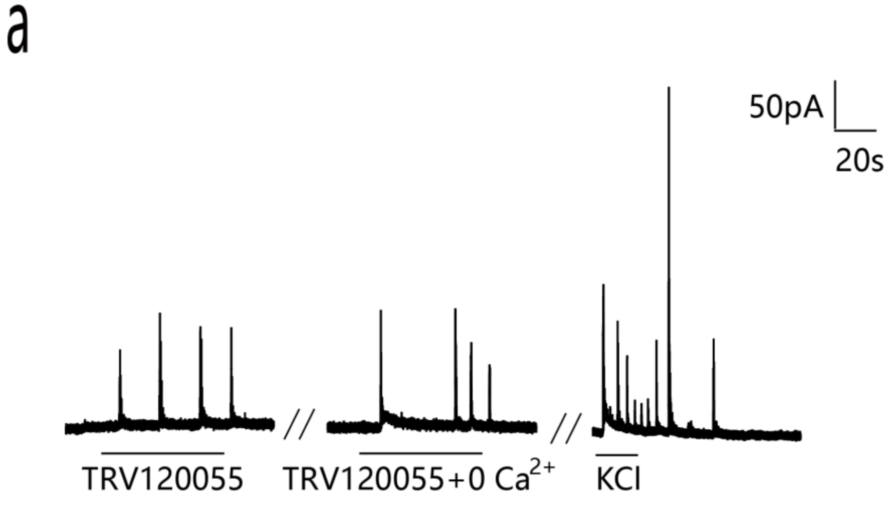

Supplementary Figure 8 TRV120055 (30 nM)-induced acute catecholamine secretion in chromaffin cells was not affected by a Ca$^{2±}$-deficient bath (related to Fig. 3l).

Supplementary Figure 9

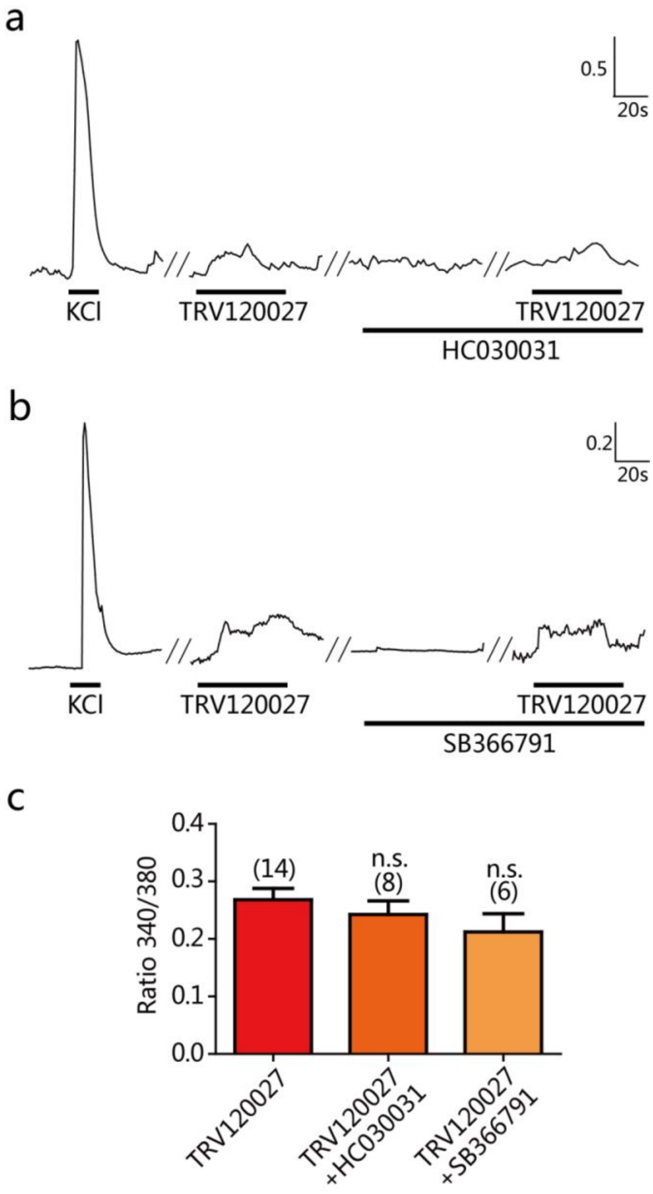

Supplementary Figure 9 TRV120027 induced $[Ca^{2+}]_i$ increase is independent of TRPA1 and TRPV1.

a. Representative curves for the effects of the TRPA1 blocker HC030031 (60μM) on TRV120027-(100nM) induced $[Ca^{2+}]_i$ elevation in primary chromaffin cells.

b. Representative curves for the effects of the TRPV1 antagonist SB366791(30μM) on TRV120027-(100nM) induced $[Ca^{2+}]_i$ elevation in primary chromaffin cells.

c. Statistical analysis and Summary bar graph of the $[Ca^{2+}]_i$ responses of the blockade in TRV120027-(100nM) induced [Ca2+]i elevation by the presence of HC030031 and

SB366791 in primary chromaffin cells. ns, no significant difference (one-way ANOVA). The bars represent mean±s.d.

Supplementary Figure 10

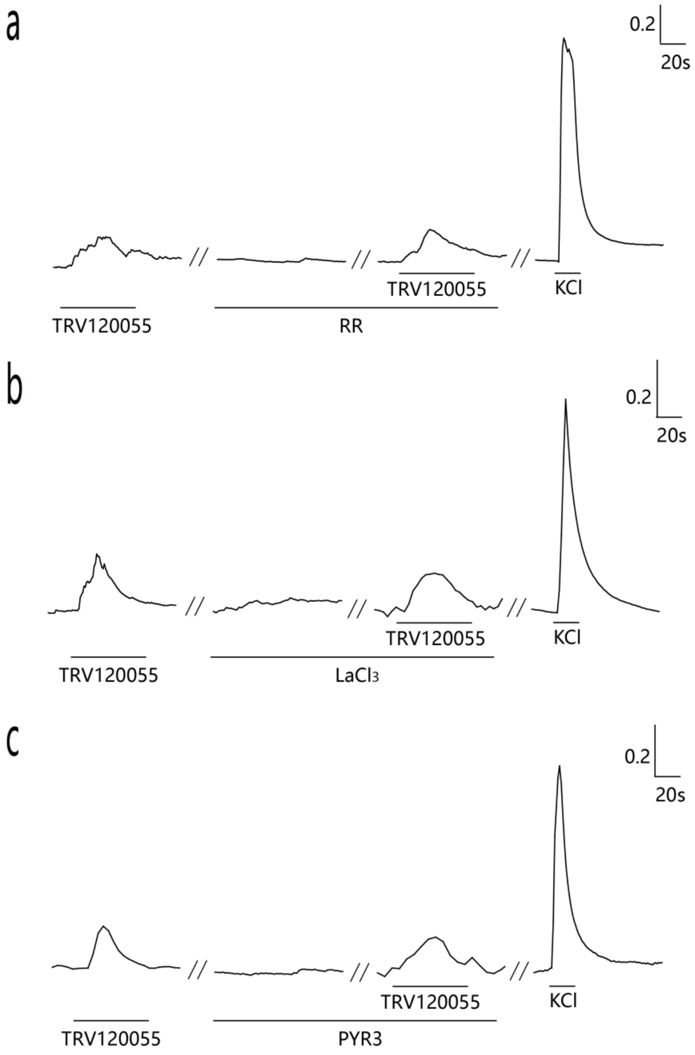

Supplementary Figure 10 Effect of different TRP channel blockers on TRV120055-induced $[Ca^{2\pm}]_i$ increase.

Representative $[Ca^{2+}]_i$ responses are shown for ruthenium red (RR，10μM)，a non-specific TRP channel blocker (a); lanthanum chloride (100μM), a non-selective TRPC3/6/7 blocker (b); and Pyr3(10μM), a selective TRPC3 blocker (c).

Supplementary Figure 11

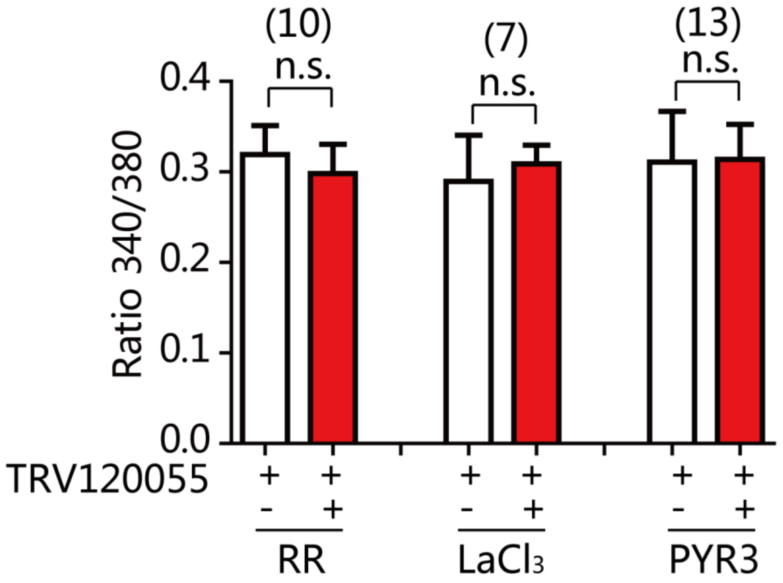

Supplementary Figure 11 Statistical analysis and summary bar graph of the $[Ca^{2\pm}]_i$ responses of the blockade in TRV120055 (30 nM)-induced [Ca2±]i elevation (Related to Supplementary Fig. 12). ns, no significant difference between the blocker treated cells compared to the vehicle-treated control cells (one-way ANOVA). The bars represent mean±s.d.

Supplementary Figure 12

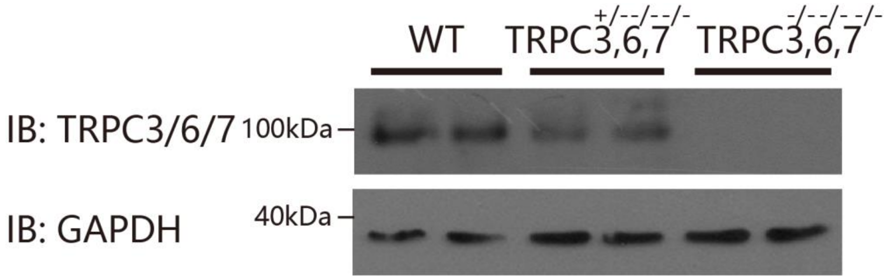

Supplementary Figure 12 Western results shows deficiency for TRPC3/6/7 knock out mice.

Western blot of TRPC3/TRPC6/TRPC7 of adrenal medulla derived from, TRPC3$^{+/-}$TRPC6$^{-/-}$TPC7$^{-/-}$ or TRPC3$^{-/-}$TRPC6$^{-/-}$TPC7$^{-/-}$ mice.

Supplementary Figure 13

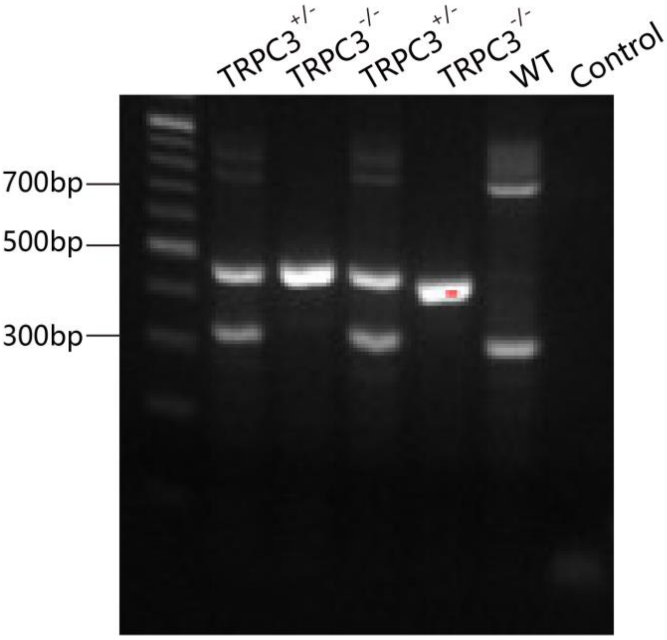

Supplementary Figure 13 Genotyping results of the TRPC3$^{-/-}$ mice

The PCR of the TRPC3$^{-/-}$ mice generates a product at 480 bp, the PCR of the wild type mice generates a product at 380 bp, and the PCR of the TRPC3$^{+/-}$ mice generates products at both 480 bp and 380 bp.

Supplementary Figure 14

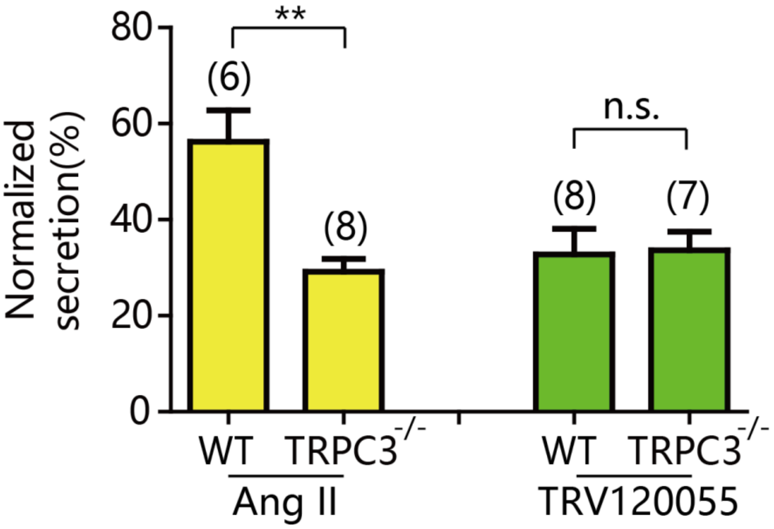

Supplementary Figure 14 Statistical analysis and summary bar graph of the acute catecholamine secretion in AngII (100 nM) or TRV12055 (30 nM)-treated

cells (Related to Fig. 4p). The TRV12055-induced acute catecholamine secretion was not significantly different between the TRPC3$^{-/-}$ mice and that of their wild type littermates. In contrast, the AngII-induced acute catecholamine secretion was significantly lower in the TRPC3$^{-/-}$ mice compared with that of their wild type littermates, which may due to the impairment of β-arrestin-1-TRPC3 signaling. **, p<0.01 (one-way ANOVA); the TRPC3$^{-/-}$ mice were compared with their wild type littermates. ns, no significant difference. The bars represent mean±s.d.

Supplementary Figure 15

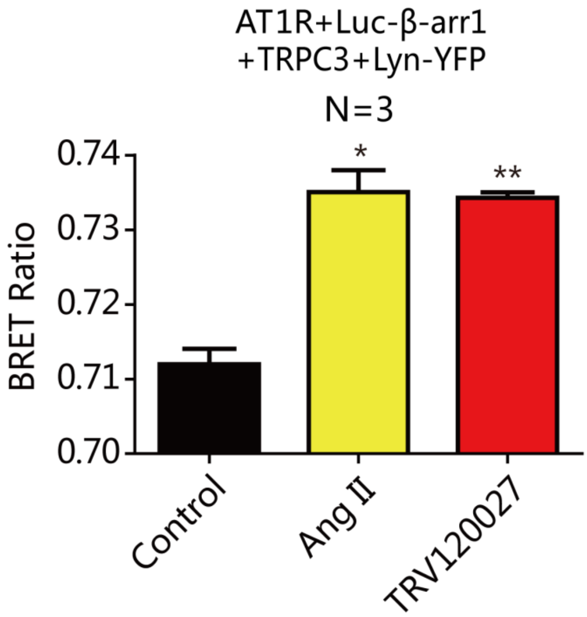

Supplementary Figure 15 Recruitment of β-arrestin-1 to the plasma membrane measured by the BRET assay. The Lyn-YFP construct was made as described[1]. HEK293 cells were co-transfected with Flag-AT1R, Luc-β-arrestin-1, TRPC3 and Lyn-YFP. After starvation, the cells were stimulated with Angiotensin II (100 nM) or TRV120027 (100 nM) for 1 min. The BRET signals were measured accordingly. *, p<0.05; **, p<0.01; (one-way ANOVA); cells stimulated by agonists were compared with the vehicle-treated control cells. The bars represent mean±s.d.

Supplementary Figure 16

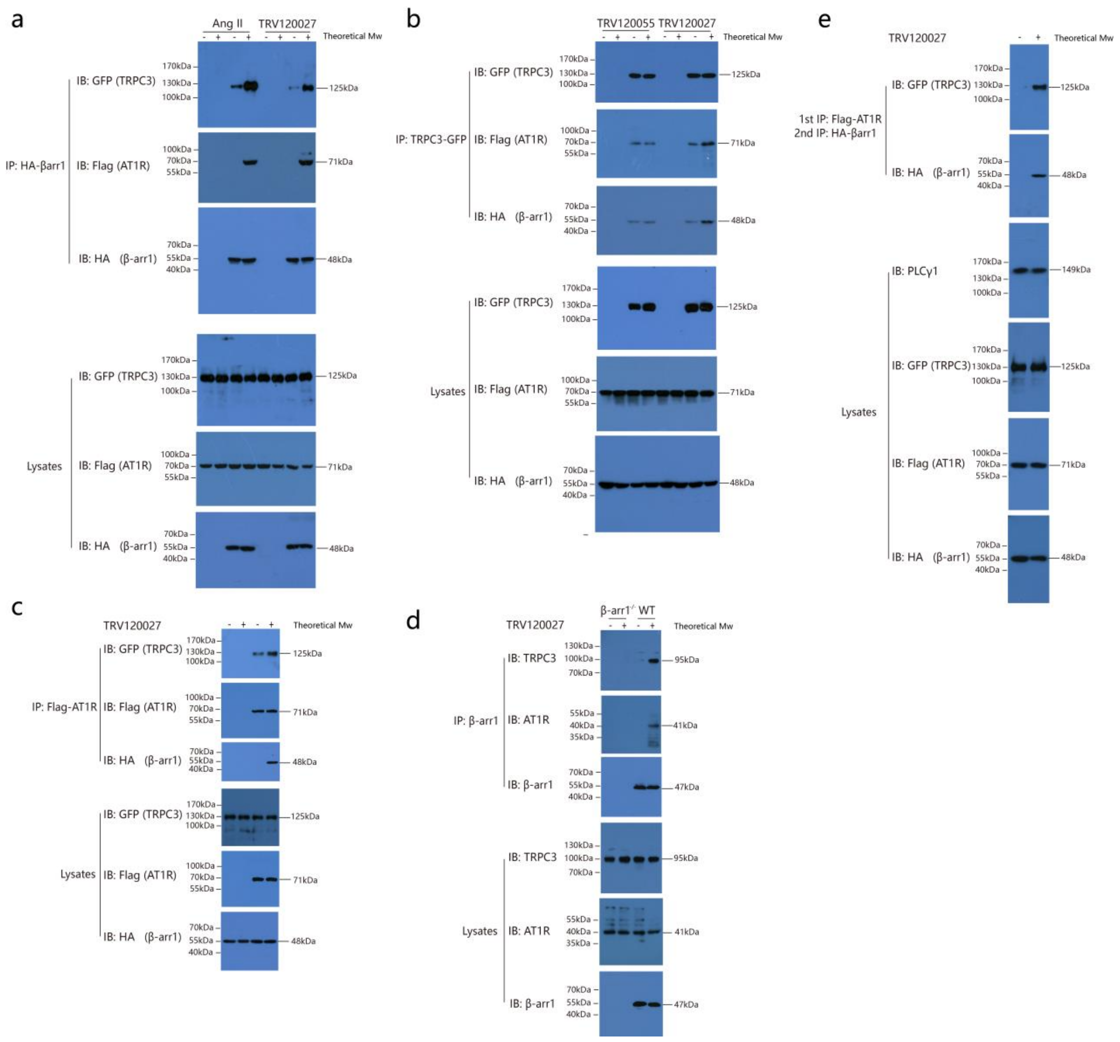

Supplementary Figure 16 Western blots in Fig. 5b， c, d, e and f are labeled with molecular weight markers (left side of each western blot) and the theoretical molecular weights (right side of each western blot) of the targeted proteins.

Supplementary Figure 17

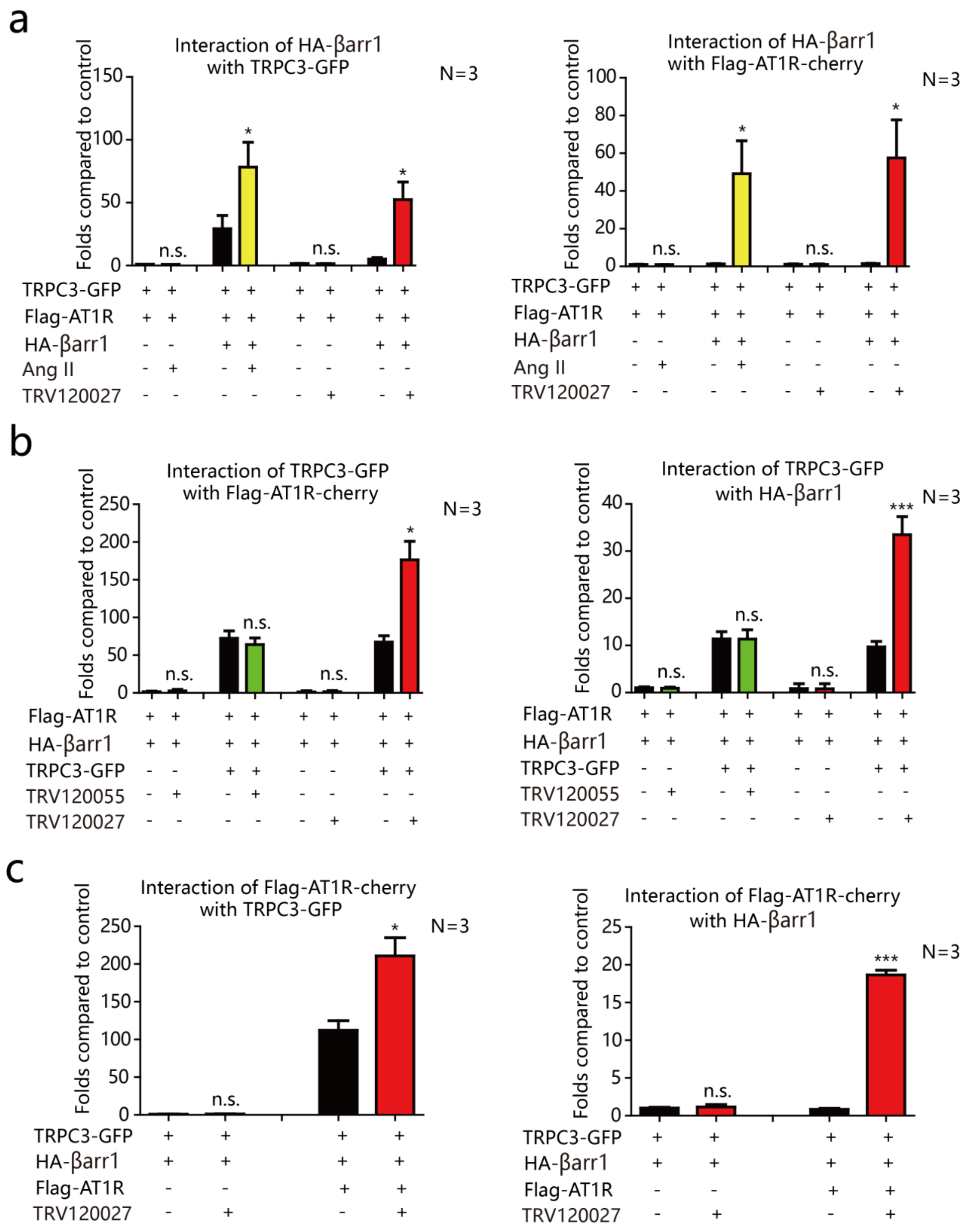

Supplementary Figure 17 TRV120027 promoted complex formation of AT1R, β-arrestin-1, and TRPC3

a. Bar graph and quantification statistics of the TRPC3-GFP or Flag-AT1R associated with the HA-β-arrestin-1 stimulated by Ang II, TRV120027 in Fig. 5b.

b. Bar graph and quantification statistics of the TRPC3-GFP associated with the Flag-AT1aR-cherry or HA-β-arrestin-1 stimulated by agonists in the Fig. 5c

c. Bar graph and quantification statistics of the Flag-AT1R associated with the TRPC3 or HA-β-arrestin-1 stimulated by agonists of Fig. 5d.

a-c) *, $p<0.05$; ***, $p<0.005$; (one-way ANOVA) different AT1R agonist treatments were compared with control vehicles. ns, no significant difference. The bars represent mean±s.d.

Supplementary Figure 18

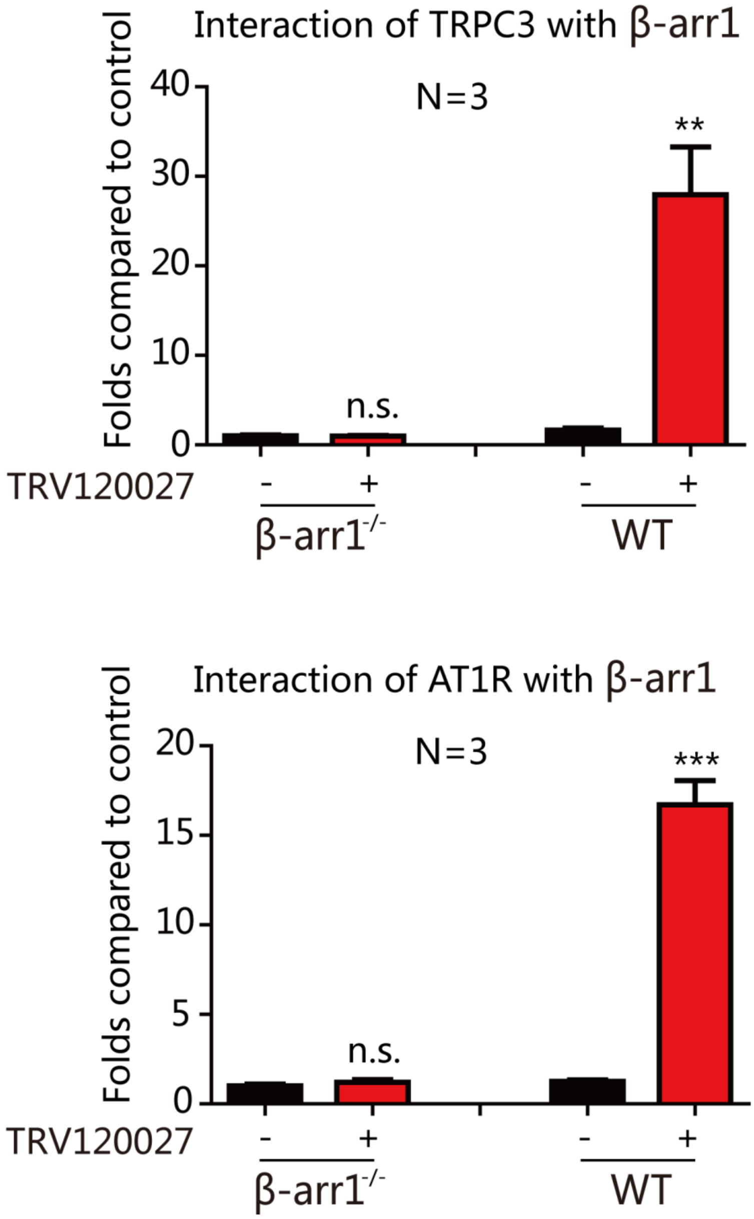

Supplementary Figure 18 Bar graph and quantification statistics of the endogenous TRPC3 or AT1R associated with the β-arrestin-1 stimulated by TRV120027 (100 nM) in Fig. 5e. **, $p<0.01$; ***, $p<0.005$ (one-way ANOVA);

TRV120027 treatments were compared with control vehicles. ns, no significant difference. The bars represent mean±s.d.

Supplementary Figure 19

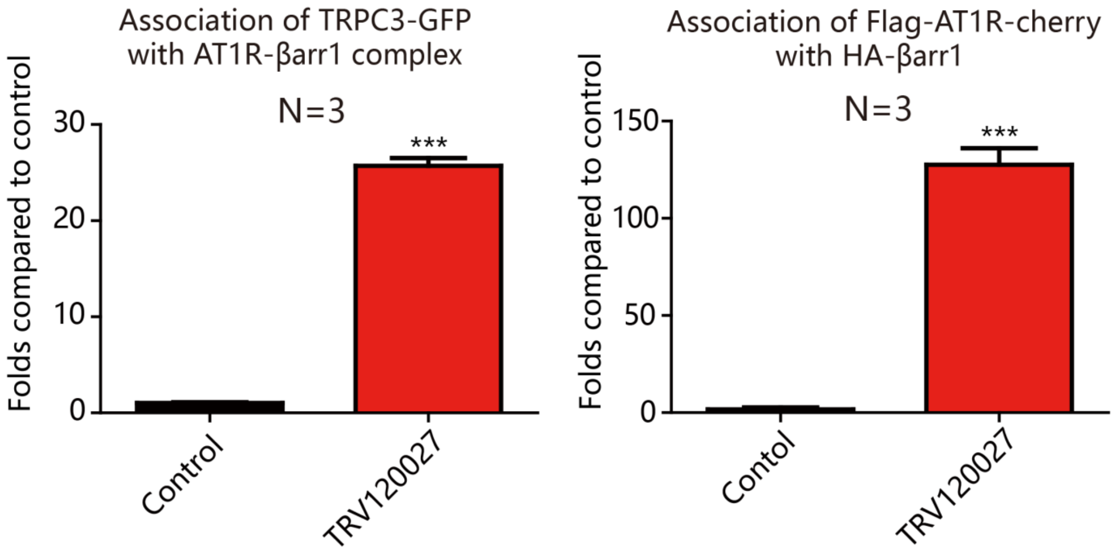

Supplementary Figure 19 Bar graph and quantification statistics of Fig. 5f Western blots represented in Fig. 5f were quantified and were shown as bar graphs. Data from at least 3 independent experiments were calculated. ***, $p<0.005$; (one-way ANOVA); TRV120027-stimulated cells were compared with vehicle-treated control cells. The bars represent mean±s.d.

Supplementary Figure 20

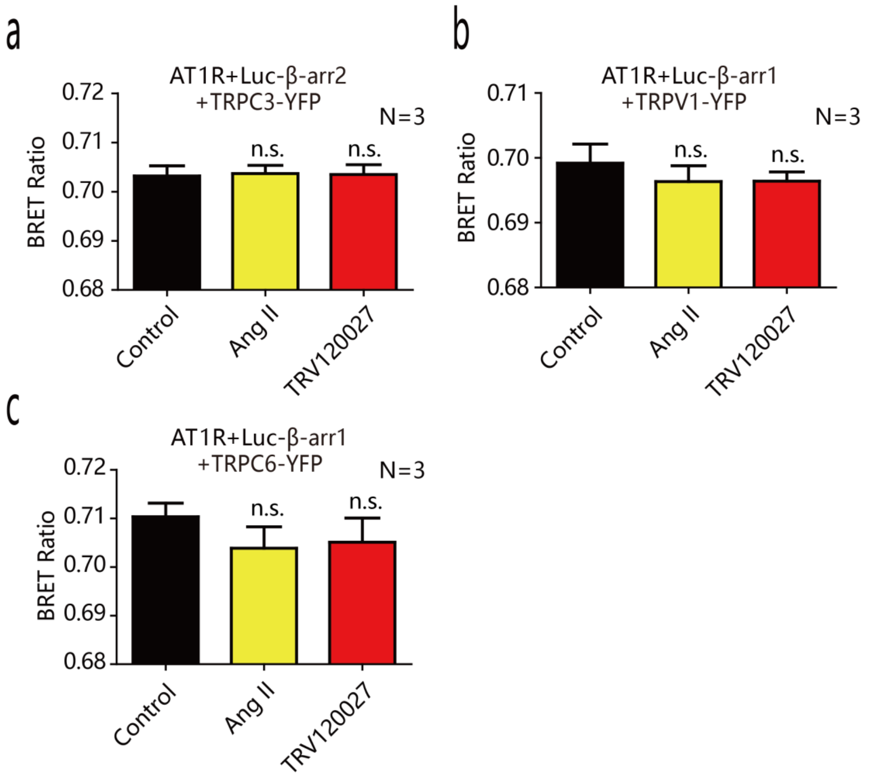

Supplementary Figure 20 BRET signal between β-arrestin-2 and TRPC3; β-arrestin-1 and TRPV1; and β-arrestin-1 and TRPC6 downstream of AT1R activation. HEK293 cells were co-transfected with Flag-AT1R, Luc-β-arrestin-2 and TRPC3-YFP (a); Flag-AT1R, Luc-β-arrestin-1 and TRPV1-YFP (b); Flag-AT1R, Luc-β-arrestin-1 and TRPC6-YFP (c). After starvation, the cells were stimulated with Angiotensin II (100 nM) or TRV120027 (100 nM) for 1 min. The BRET signals were measured accordingly. ns, no significant differences (one-way ANOVA). The bars represent mean±s.d.

Supplementary Figure 21

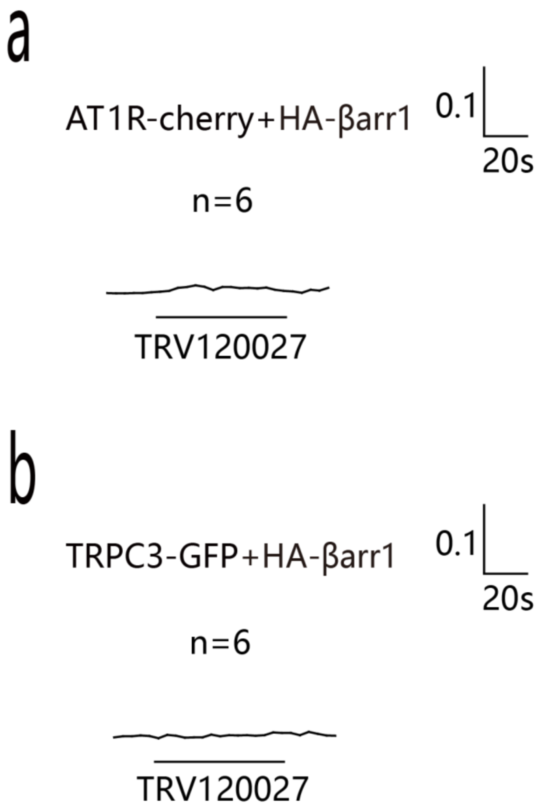

Supplementary Figure 21 Three components, AT1R, β-arrestin-1 and TRPC3, are required for TRV120027 induced calcium increase in HEK293 cells. Representative TRV120027-induced $[Ca^{2\pm}]_i$ responses of HEK293 cells co-transfected with Flag-AT1R-Cherry and HA-β-arrestin-1 (a) or with TRPC3-GFP and HA-β-arrestin-1 (b).

Supplementary Figure 22

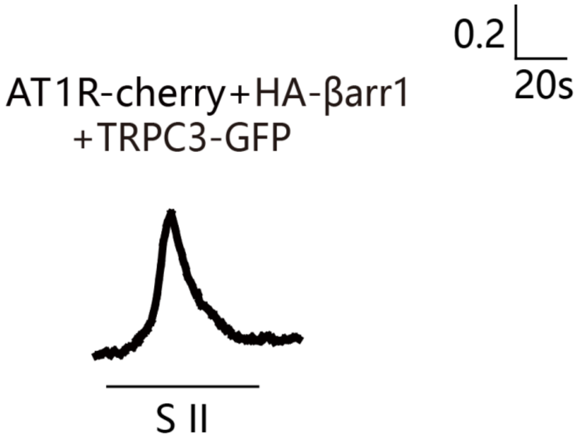

Supplementary Figure 22 Representative $[Ca^{2\pm}]_i$ responses of HEK293 cells co-transfected with Flag-AT1R-Cherry, HA-β-arrestin-1 and TRPC3-GFP to SII (1 μM).

Supplementary Figure 23

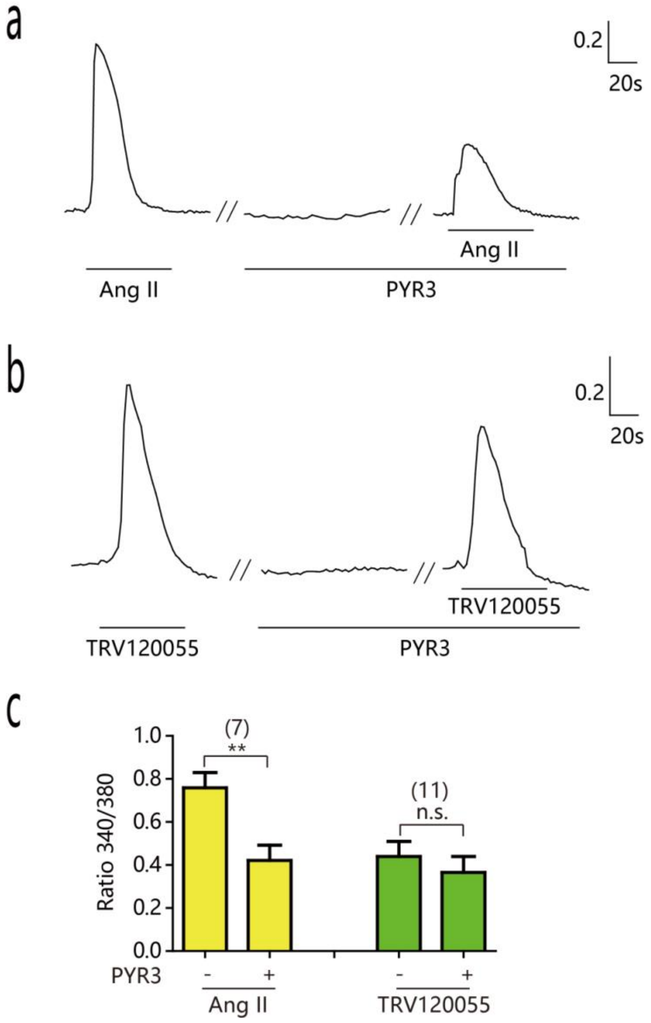

Supplementary Figure 23 Effects of Pyr3 on AngII or TRV120055-induced $[Ca^{2\pm}]_I$ increase in the recombinant HEK293 system.

HEK293 cells were co-transfected with Flag-AT1R-Cherry, HA-β-arrestin-1 and TRPC3-GFP. a. Representative $[Ca^{2\pm}]_i$ responses to AngII (100 nM), with or without Pyr3 (10 μM) incubation.

b. Representative $[Ca^{2\pm}]_i$ responses to TRV120055 (30 nM), with or without Pyr3 (10 μM) incubation.

c. Bar graph and quantification statistics of $[Ca^{2\pm}]_i$ responses in supplementary Fig. 23a and 23b. **, $p<0.01$; (one-way ANOVA); Pyr3 treatments were compared with control vehicles. ns, no significant differences. The bars represent mean±s.d.

Supplementary Figure 24

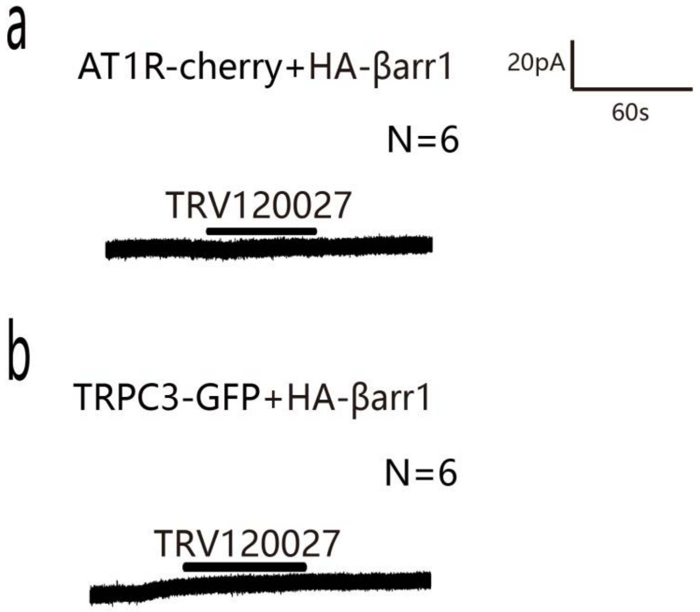

Supplementary Figure 24 Three components, AT1R, β-arrestin-1 and TRPC3, are required for TRV120027-induced TRPC3 currents in HEK293 cells. No significant TRV120027-induced inward current in HEK293 cells co-transfected with only Flag-AT1R-cherry and HA-β-arrestin-1 (a) or HA-β-arrestin-1 and TRPC3-GFP (b) were observed.

Supplementary Figure 25

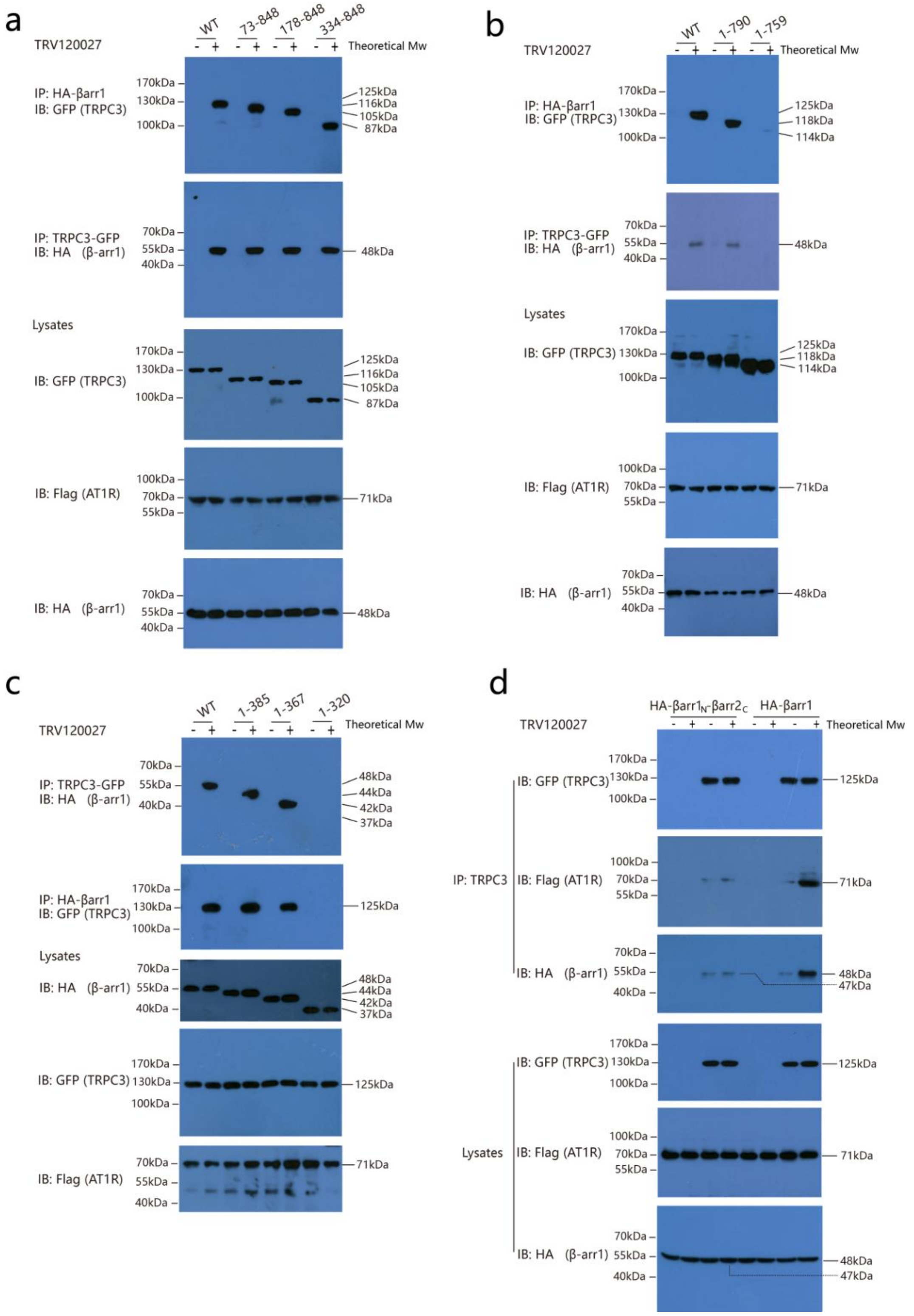

Supplementary Figure 25 Western blots in Fig. 6 b, c, e and f were labeled with molecular weight markers (left side of each western blot) and the theoretical molecular weights (right side of each western blot) of the targeted proteins.

Supplementary Figure 26

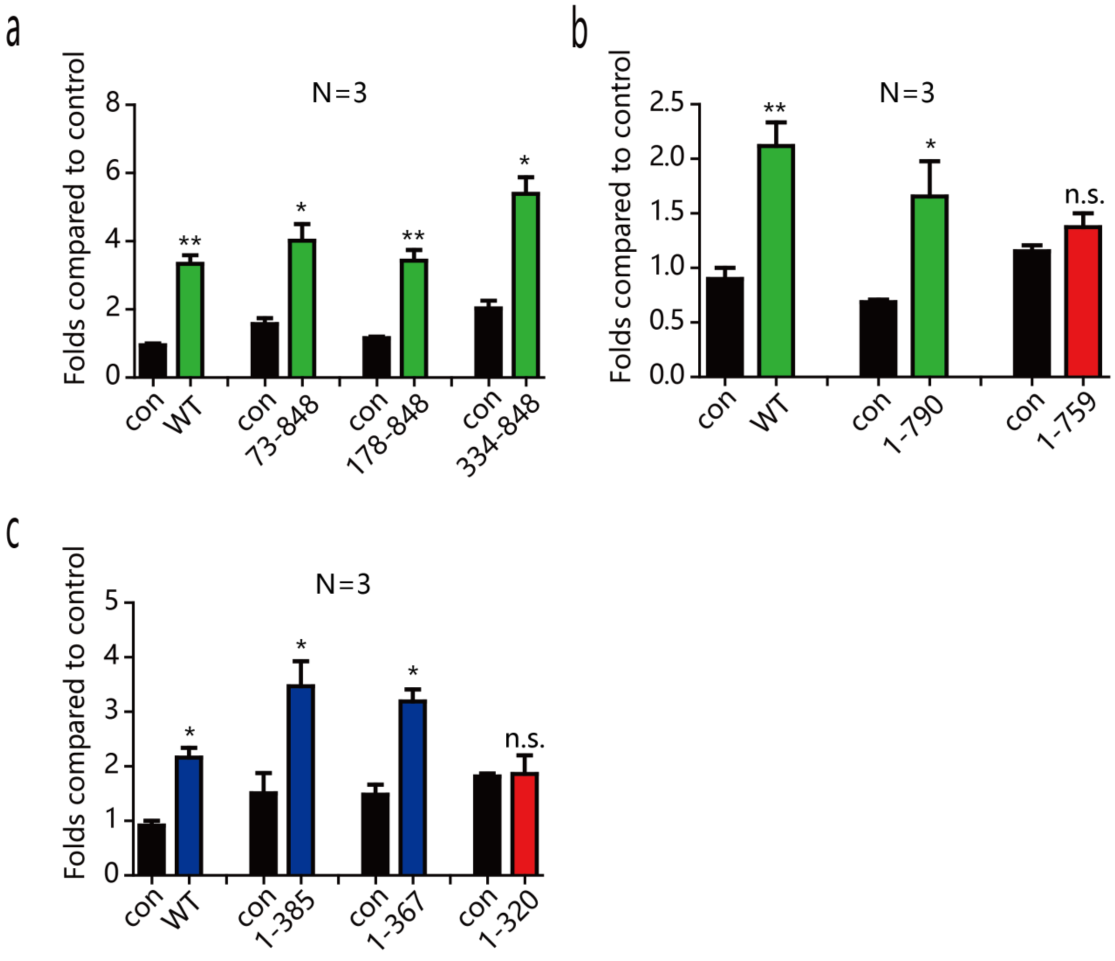

Supplementary Figure 26 Mapping the interface between the TRPC3 and β-arrestin-1

a. Bar graph and quantification statistics of the TRPC3-GFP-WT or different TRPC3 N-terminal deletions associated with the HA-β-arrestin-1-WT stimulated by TRV120027 in Fig. 6b.

b. Bar graph and quantification statistics of the TRPC3-GFP-WT or different TRPC3 C-terminal deletions associated with the HA-β-arrestin-1-WT stimulated by TRV120027 in Fig. 6c.

c. Bar graph and quantification statistics of the HA-β-arrestin-1-WT or its different C-terminal deletions associated with the TRPC3-GFP-WT stimulated by TRV120027 in Fig. 6e.

(a-c) All statistics of the immunoprecipitation results were normalized to the expression level of the target proteins in the lysates. (densitometry of the band in first

lane were normalized by densitometry of the band in the third lane) *, $p<0.05$; **, $p<0.01$; (one-way ANOVA); different truncations of TRPC3-GFP or HA-β-arrestin-1 were compared with wild type. ns, no significant differences. The bars represent mean±s.d.

Supplementary Figure 27

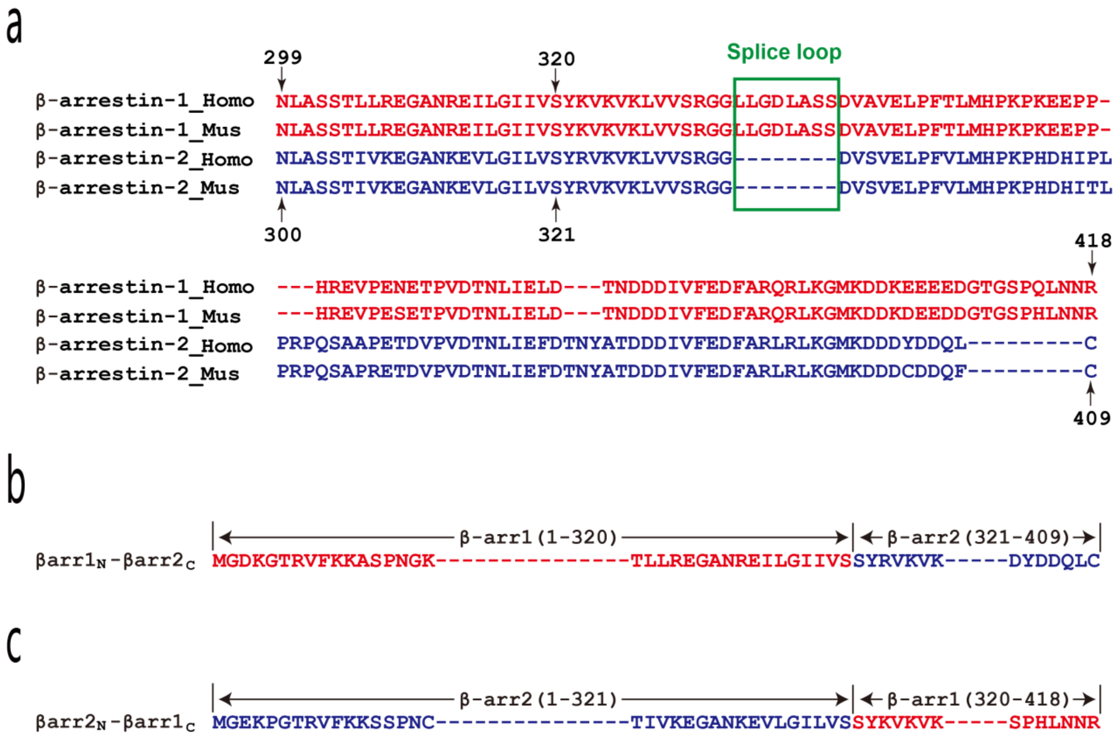

Supplementary Figure 27 Schematic representation of the construction of the chimeric protein βarr1$_N$-βarr2$_C$.

a. Sequence alignment of the C-terminal region of β-arrestin-1 and β-arrestin-2 from Homo sapiens and Mus muscles
b. Chimeric protein βarr1$_N$-βarr2$_C$ was made by conjugation of the N-terminal of β-arrestin-1 (1-320) to the C-terminal of the β-arrestin-2 (321-409)
c. Chimeric protein βarr2$_N$-βarr1$_C$ was made by conjugation of the N-terminal of β-arrestin-2 (1-321) to the C-terminal of the β-arrestin-1 (320-418)

Supplementary Figure 28

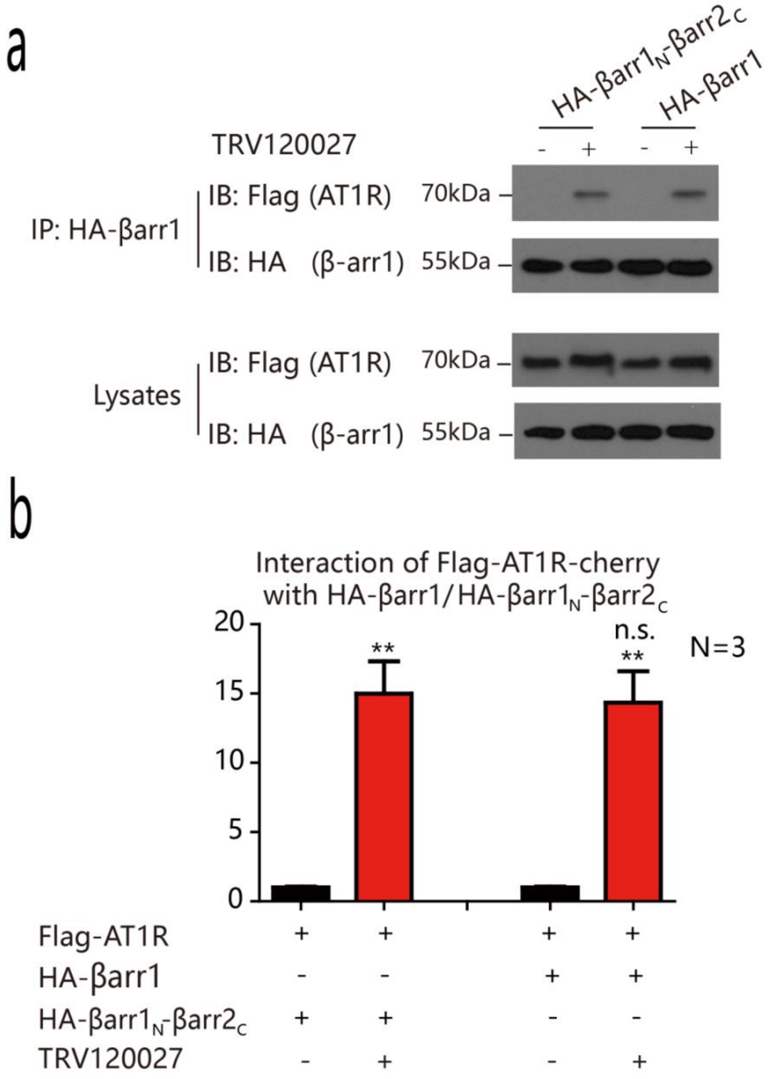

Supplementary Figure 28 TRV120027-induced AT1R-βarr1$_N$-βarr2$_C$ complex formation.

(a) HEK293 cells co-transfected with Flag-AT1R-cherry and HA-β-arrestin-1-WT or HA-βarr1$_N$-βarr2$_C$ were stimulated with TRV120027 (100nM) for 1 min. The HA-β-arrestin-1-WT or HA-βarr1$_N$-βarr2$_C$ was immunoprecipitated with the anti-HA beads; the association of AT1R with HA-β-arrestin-1 or HA-βarr1$_N$-βarr2$_C$ was detected by western blot. A representative western blot from at least three independent experiments is shown.

(b) Bar graph and quantification statistics of the Supplementary Fig. 28a. **, $p<0.01$; TRV120027-stimulated cells were compared with the vehicle-treated control cells. ns, no significant difference between the HA-β-arrestin-1-WT and HA-βarr1$_N$-βarr2$_C$ transfected cells (one-way ANOVA). The bars represent mean±s.d.

Supplementary Figure 29

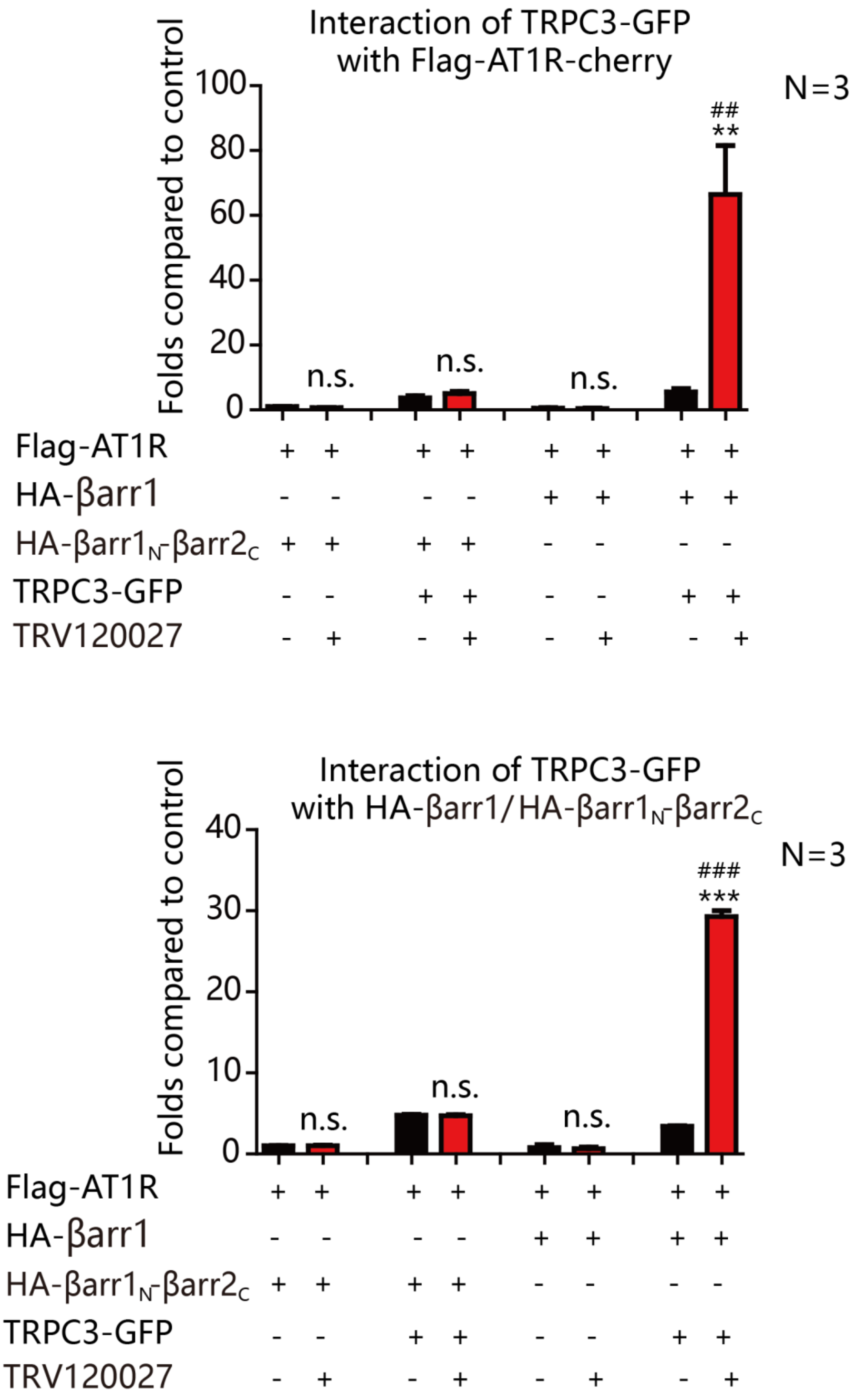

Supplementary Figure 29 Bar graph and quantification statistics of Fig. 6f

**, $p<0.01$; ***, $p<0.005$; TRV120027-stimulated cells were compared with vehicle-treated control cells. ##, $p<0.01$; ###, $p<0.005$; HA-βarr1$_N$-βarr2$_C$ transfected cells were compared with HA-β-arrestin-1-WT transfected cells. The bars represent mean±s.d and the data were analyzed using one-way ANOVA.

Supplementary Figure 30

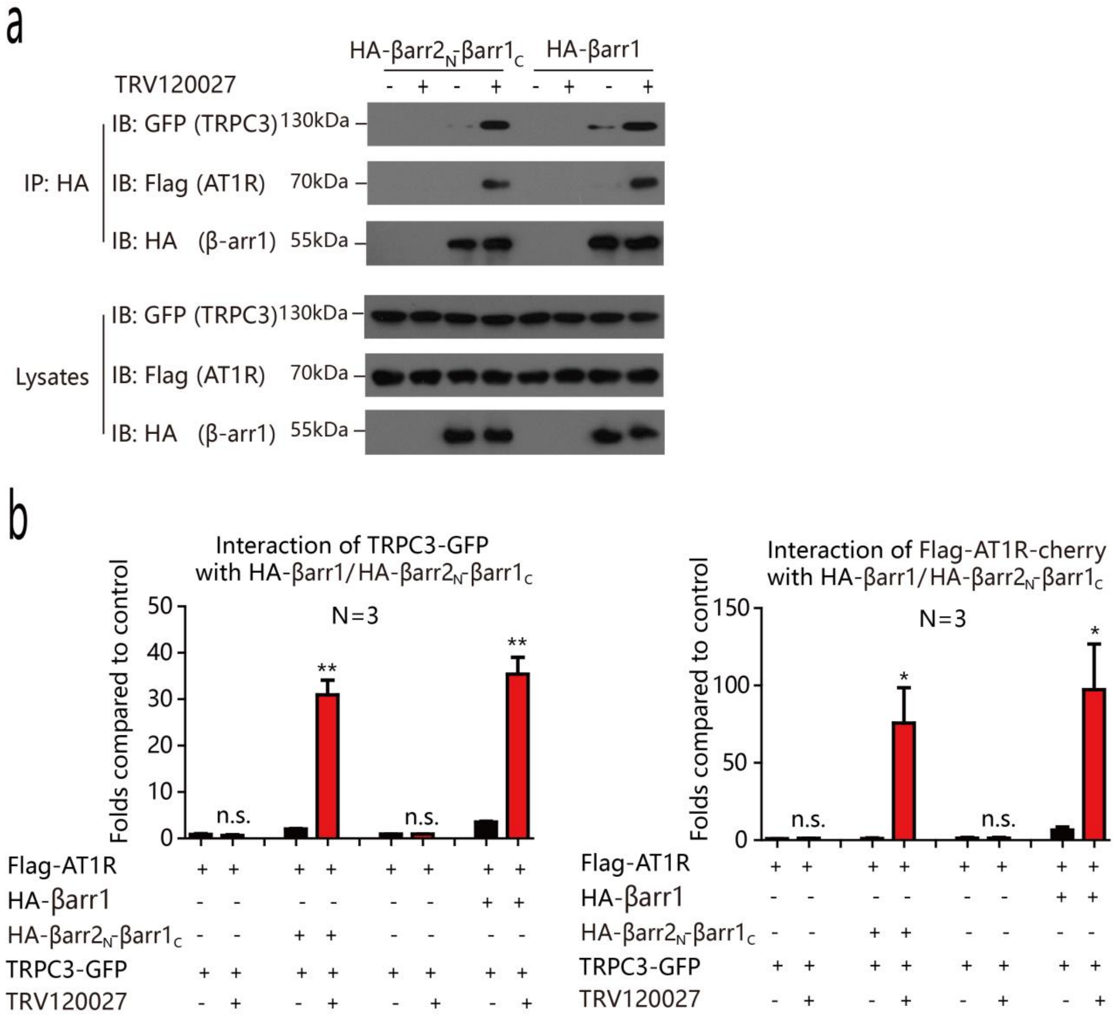

Supplementary Figure 30 TRV120027 stimulates the association of HA-β-arrestin-1-WT or HA-βarr2$_N$-βarr1$_C$ with TRPC3 or AT1R

(a) HEK293 cells co-transfected with Flag-AT1R-cherry, TRPC3-GFP with or without HA-β-arrestin-1-WT or HA-βarr2$_N$-βarr1$_C$ were stimulated with TRV120027 (100 nM) for 1 min. The HA-tagged protein was immunoprecipitated with the anti-HA beads. The association of the HA-β-arrestin-1 with the TRPC3 or AT1R was detected by western.

(b) Bar graph and quantification statistics of the Supplementary Fig. 30a. *, $p<0.05$; **, $p<0.01$; TRV120027-stimulated cells were compared with vehicle-treated control cells. ns, no significant difference. The bars represent mean±s.d and the data were analyzed using one-way ANOVA.

Supplementary Figure 31

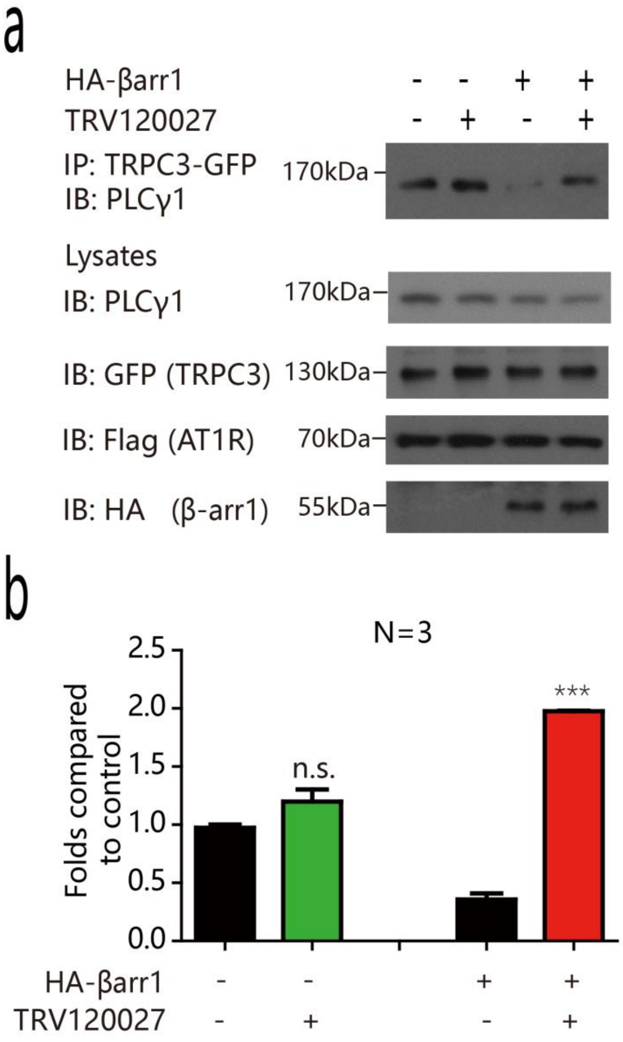

Supplementary Figure 31 TRV120027 stimulates complex formation between PLCγ and TRPC3

(a) β-arrestin-1 biased ligand TRV120027 (100nM) stimulates complex formation between PLCγ and TRPC3.

(b) Bar graph and quantification statistics of the Supplementary Fig. 31a. ***, $p<0.005$; TRV120027-stimulated cells were compared with vehicle-treated control cells. ns, no significant difference. The bars represent mean±s.d and the data were analyzed using one-way ANOVA.

In HEK293 cells overexpressing AT1R and TRPC3, a portion of PLCγ constitutively interacted with TRPC3, and this interaction was not affected by TRV120027 administration. However, in cells co-transfected with AT1R, β-arrestin-1 and TRPC3, more than 3-fold the amount of PLCγ was associated with TRPC3 after TRV120027 stimulation (Supplementary Fig. 32). We noticed that without TRV120027

administration, the basal association of GFP-TRPC3 and PLC-γ decreased in the cells with HA-β-arrestin-1 overexpression. We speculate that these phenotypes were due to the basal interaction between the PLC-γ and β-arrestin-1. The overexpression of β-arrestin-1 sequestered PLC-γ into the intracellular portion, which decreased the basal interaction between TRPC3 and PLC-γ in the rest state. In response to TRV120027 application, a fraction of β-arrestin-1 was recruited to TRPC3, which also promoted the association between PLC-γ and TRPC3.

Supplementary Figure 32

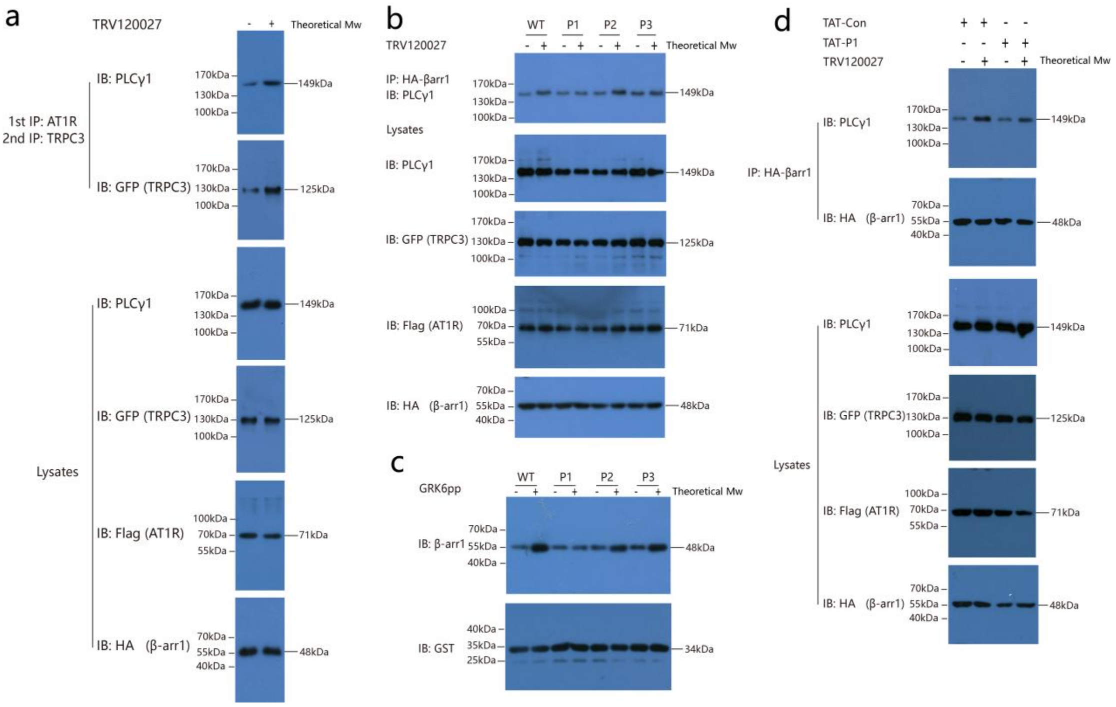

Supplementary Figure 32 Western blots in Fig.7 a, d, e and g were labeled with molecular weight marker （left side of each western blot）and the theoretical molecular weights (right side of each western blot) of the targeted proteins.

Supplementary Figure 33

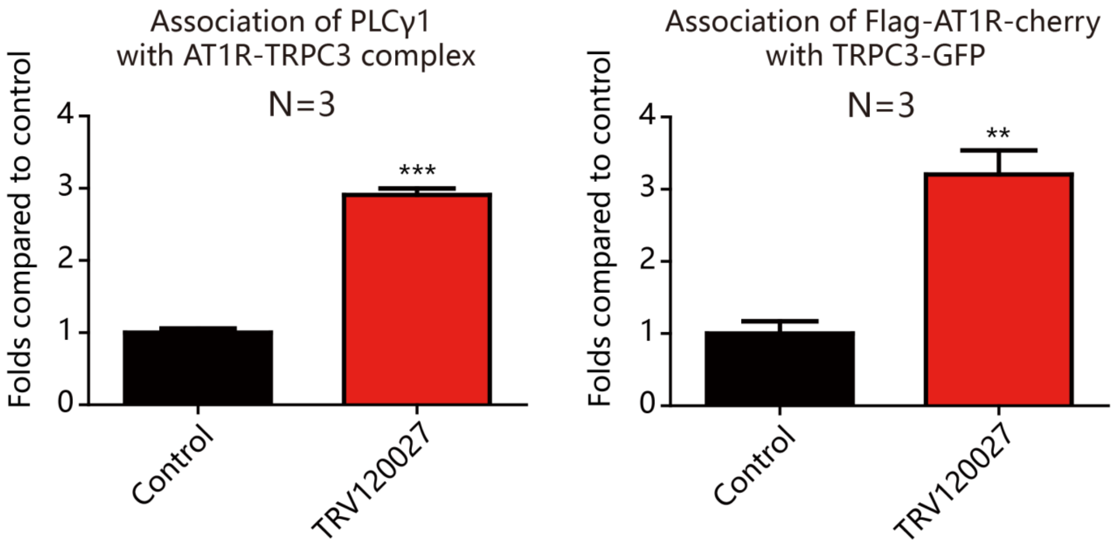

Supplementary Figure 33 Bar graph and quantification statistics of Fig. 7a

Western blots represented in Fig. 7a were quantified and were shown as bar graphs. Data from at least 3 independent experiments were calculated. **, $p<0.01$;***, $p<0.005$; TRV120027-stimulated cells were compared with vehicle-treated control cells. The bars represent mean±s.d and the data were analyzed using one-way ANOVA.

Supplementary Figure 34

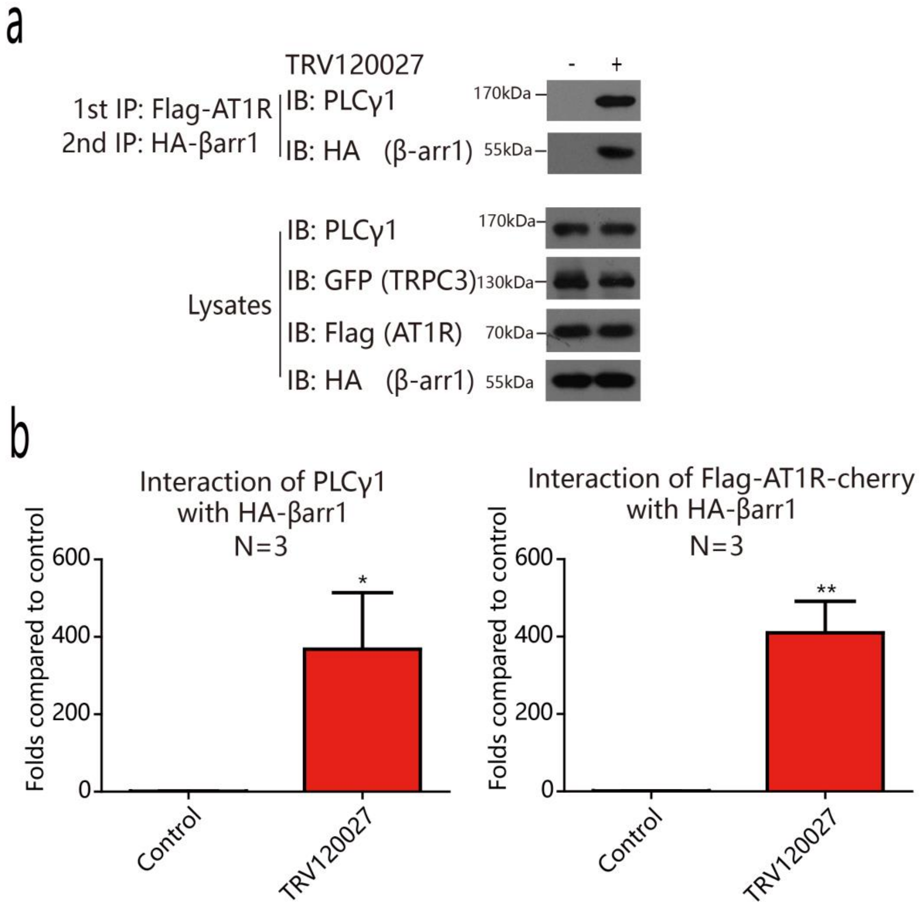

Supplementary Figure 34 TRV120027 stimulates complex formation between PLCγ1 and Flag-AT1R-HA-β-arrestin-1

(a) Sequential immunoprecipitation experiments. HEK293 cells co-transfected with Flag-AT1R-cherry, HA-β-arrestin-1 and TRPC3-GFP were stimulated with TRV120027 (100 nM) or control vehicle for 1 min. The plasma membrane fractions were first isolated by centrifugation. The protein complexes containing Flag-AT1R were immunoprecipitated by Anti-Flag M2 agarose and then eluted with 3*Flag peptide. The complexes containing Flag-AT1R were then immunoprecipitated by anti-HA agarose. PLCγ1 associated with Flag-AT1R-HA-β-arrestin-1 was detected by a specific antibody.

(b) Bar graph and quantification statistics of the Supplementary Fig. 34a. Data are collected from 3 independent experiments. *, $p<0.05$;**, $p<0.01$; TRV120027-stimulated cells were compared with vehicle-treated control cells. The bars represent mean±s.d and the data were analyzed using one-way ANOVA.

Supplementary Figure 35

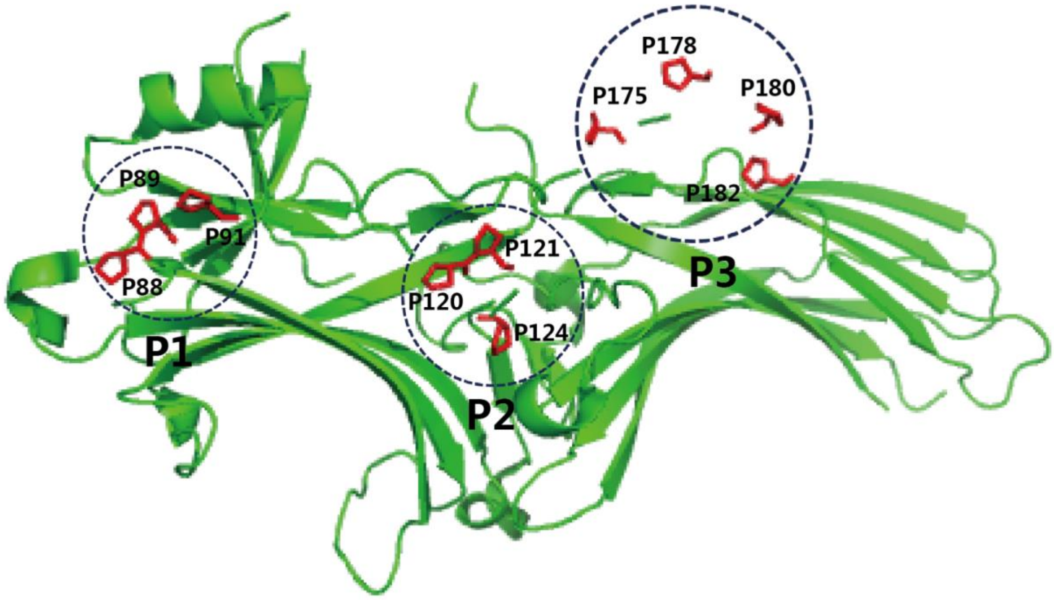

Supplementalry Figure 35 Structural representation and sequence information for the poly-proline region of the β-arrestin-1

Ribbon diagram and the frontal view of the three poly proline sites in the inactive β-arrestin-1 crystal structure (PDB: 1G4M, green). The secondary structure was shown in green and the poly prolines were shown in red.

Supplementary Figure 36

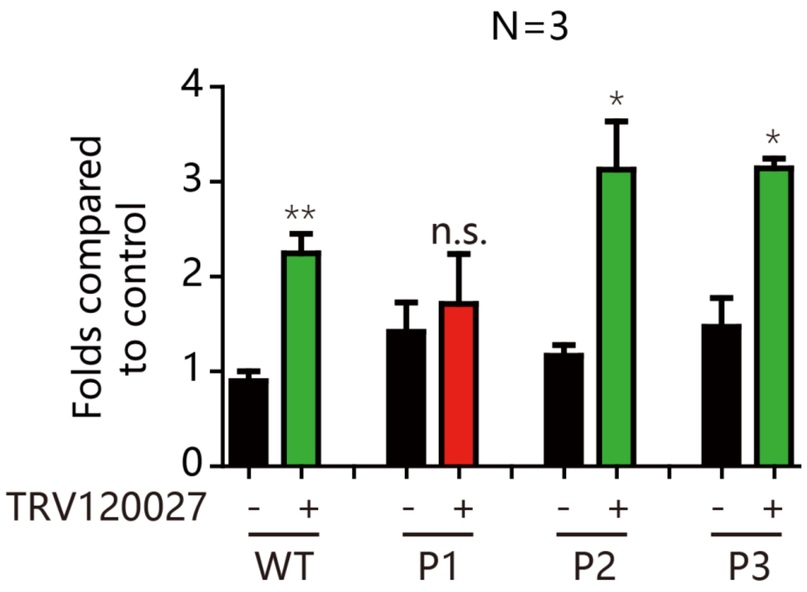

Supplementary Figure 36 TRV120027 induced β-arrestin-1-PLCγ1 complex formation through the poly-proline region 1 of the β-arrestin-1

Bar graph and quantification statistics of the PLCγ1 associated with HA-β-arrestin-1 WT or different HA-β-arrestin-1 mutants in Fig. 7d.

*, $p<0.05$; **, $p<0.01$, TRV120027 treatment was compared with control vehicles. ns, no significant difference. The bars represent mean±s.d and the data were analyzed using one-way ANOVA.

Supplementary Figure 37

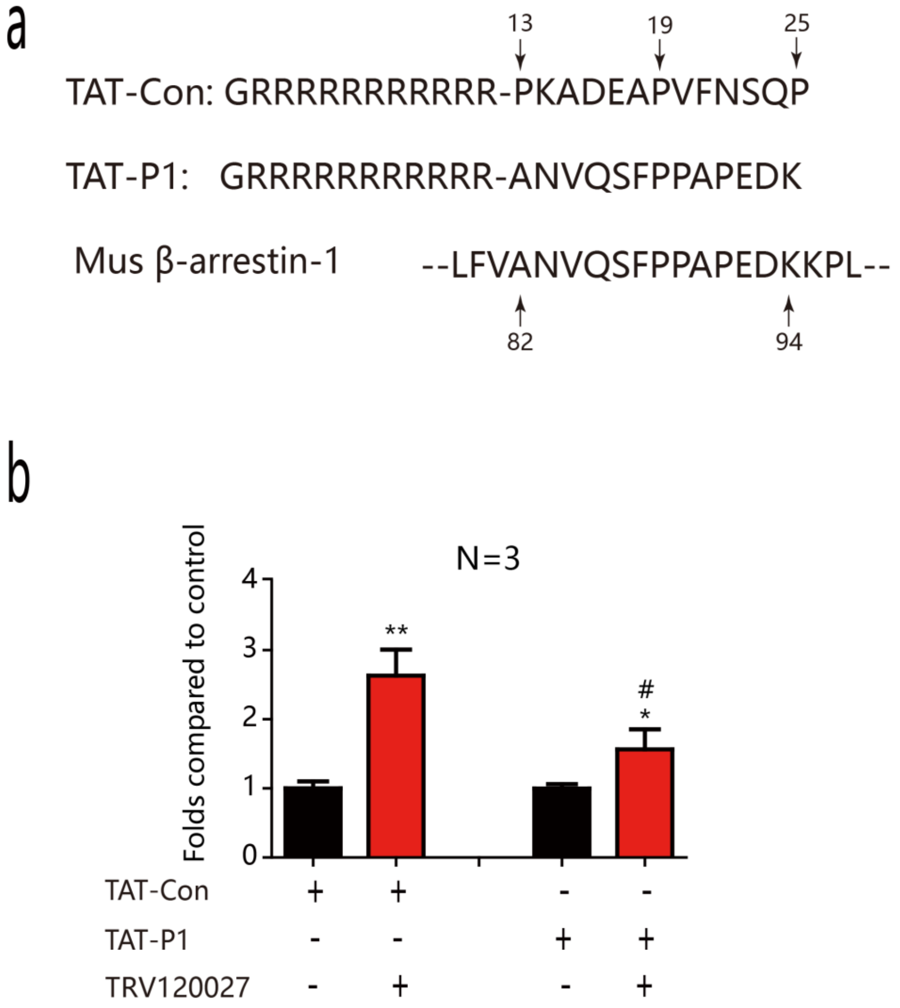

Supplementary Figure 37 Schematic representation of the construction of the TAT-P1 peptide and the quantification analysis.

a. Strategy to make the TAT-P1. The Poly-proline region P1 of β-arrestin-1(82-94) was linked to the C-terminal of the HIV-TAT sequence. A random poly-proline region peptide was fused to HIV-TAT to serve as a control (TAT-Con).

b. Bar graph and quantification statistics of Fig. 7g. *, $p<0.05$; **, $p<0.01$; TRV120027-treated cells were compared with vehicle-treated control cells. #, $p<0.05$, TAT-P1 treated cells were compared with TAT-Con treated cells. The bars represent mean±s.d and the data were analyzed using one-way ANOVA.

Supplementary Figure 38

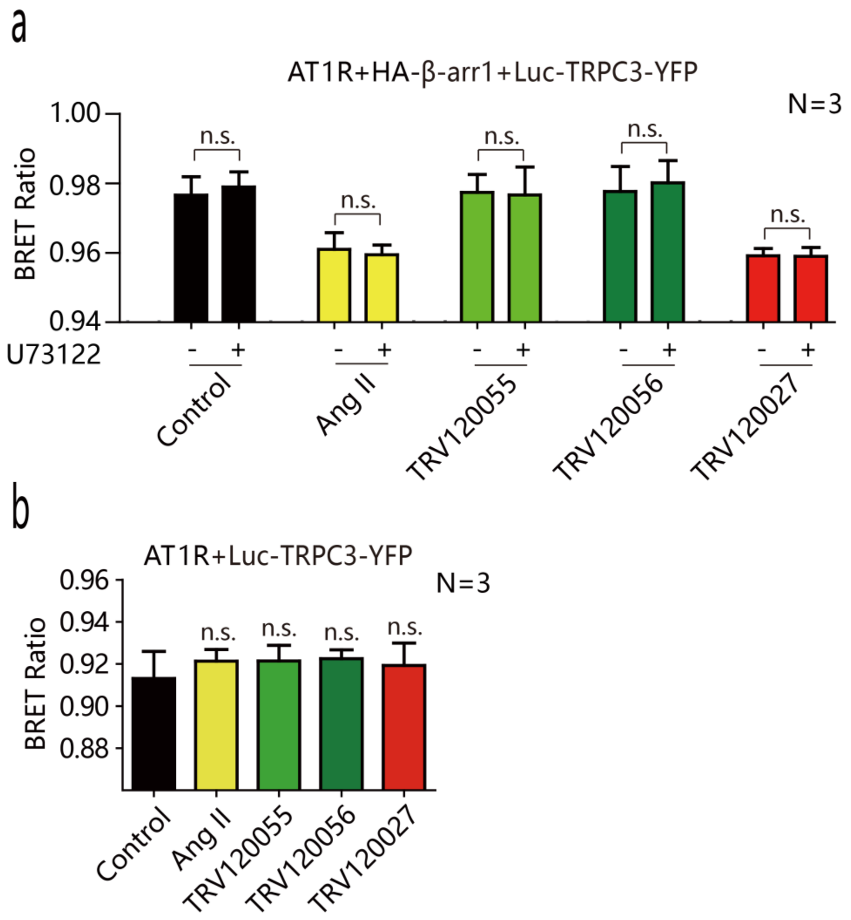

Supplementary Figure 38 Conformational change of TRPC3 detected by BRET assays.

a. The effect of U73122 (10 μM) on the BRET signal of Luc-TRPC3-YFP after stimulation with different AT1R agonists or incubation with control vehicles.

b. The AT1R agonists could not induce detectable BRET signals in HEK293 cells co-transfected with only AT1R and Luc-TRPC3-YFP. ns, no significant difference. The bars represent mean±s.d and the data were analyzed using one-way ANOVA.

Supplementary Figure 39

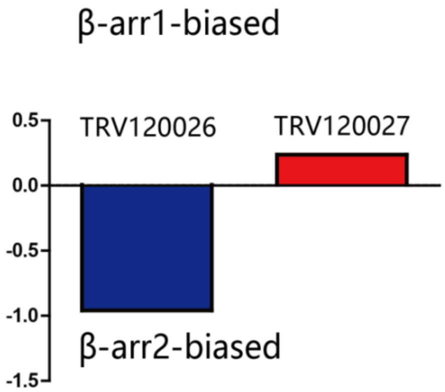

Supplementary Figure 39 Bar graph of Bias factors (β) calculated from equiactive comparison using AngII as a reference molecule. HEK293 cells were co-transfected with AT1R-YFP, Luc-β-arrestin-1 or Luc-β-arrestin-2. The dose response curves of the BRET experiments were used to derive the molecular efficacy for either β-arrestin-1 or β-arrestin-2 recruitment by different ligands. Positive bias values (red) represent β-arr1-biased molecular efficacies, whereas negative values (blue) represent β-arr2-biased molecular efficacies.

Supplementary Table1: Information for peptide ligands of AT1R.

Ligand	Peptide Sequence	Agonist type
Ang II	Asp-Arg-Val-Tyr-Ile-His-Pro-Phe	Full agonist
TRV120055	Gly-Val-Tyr-Ile-His-Pro-Phe	Gq-biased
TRV120056	Asp-Arg-Gly-Val-Tyr-Ile-His-Pro-Phe	Gq-biased
S II	Sar-Arg-Val-Ile-Ile-His-Pro-Ile	β-arrestin-biased
TRV120026	Sar-Arg-Val-Tyr-Tyr-His-Pro-NH_2	β-arrestin-biased
TRV120027	Sar-Arg-Val-Tyr-Ile-His-Pro-(D-Ala)	β-arrestin-biased

All the peptides were purchased from China Peptides Co., Ltd. (Shanghai, China).

Supplementary Table2: Molecular efficacy of the β-arrestin-1 or β-arrestin-2 recruitment to AT1R induced by different β-arrestin biased ligand,as detected by BRET assay.

Ligand	β-arrestin-1		β-arrestin-2			
	EC50(nM)	Emax($*10^{-2}$)	EC50(nM)	Emax($*10^{-2}$)	β	bias
Ang II	1.75±0.62	1.24±0.86	8.31±0.16	2.25±0.23	0.00	
TRV120026	7.71±0.10	5.29±0.32	0.58±0.24	1.36±0.54	-0.96	β-arr2
TRV120027	1.21±0.37	0.29±0.13	18.07±0.33	0.96±0.16	0.24	β-arr1
