# Supplementary material for: Arrestin-biased AT1R agonism induces acute catecholamine secretion through TRPC3 coupling
Source: Nat Commun. 2017 Feb 9;8:14335. doi: 10.1038/ncomms14335 (PMC5309860; doi:10.1038/ncomms14335)
Supplement: Supplementary Data 1 — Nucleotide sequences of plasmids. Nucleotide sequences of cDNAs of HA-βarr1-WT, HA-βarr1 1-320, HA-βarr1 1-367, HA-βarr1 1-385, βarr1-RFP, Luc-βarr1, Luc-βarr2, HA-βarr1N-βarr2C, HA-βarr2N-βarr1C, TRPC3-GFP-WT, TRPC3-GFP 73-848, TRPC3-GFP 178-848, TRPC3-GFP 334-848, TRPC3-GFP 1-790, TRPC3-GFP 1-759, TRPC3-YFP, Luc-TRPC3-YFP, Flag-AT1R-cherry, Flag-AT1R, Lyn-YFP [file ncomms14335-s2.docx]

**Supplementary Data 1. Nucleotide sequences of plasmids.**

Nucleotide sequences of cDNAs of HA-βarr1-WT, HA-βarr1 1-320, HA-βarr1 1-367, HA-βarr1 1-385, βarr1-RFP, Luc-βarr1, Luc-βarr2, HA-βarr1_N_-βarr2_C,_ HA-βarr2_N_-βarr1_C,_ TRPC3-GFP-WT, TRPC3-GFP 73-848, TRPC3-GFP 178-848, TRPC3-GFP 334-848, TRPC3-GFP 1-790, TRPC3-GFP 1-759, TRPC3-YFP, Luc-TRPC3-YFP, Flag-AT1R-cherry, Flag-AT1R, Lyn-YFP

**HA-βarr1-WT**

ATGGGCGACAAAGGGACACGAGTGTTCAAGAAGGCAAGCCCCAATGGAAAGCTCACCGTCTACCTGGGAAAGCGGGACTTTGTGGACCACATTGACCTGGTGGACCCCGTGGATGGCGTGGTCCTGGTGGATCCTGAGTATCTCAAAGAAAGGCGAGTCTACGTGACACTGACCTGCGCCTTCCGGTATGGCCGGGAAGACCTGGATGTCTTGGGTCTGACTTTTCGCAAAGACCTGTTTGTGGCTAACGTGCAGTCCTTCCCACCGGCCCCTGAGGACAAGAAGCCACTGACTCGGCTACAAGAGCGACTCATCAAGAAGCTGGGCGAGCATGCCTACCCCTTCACCTTTGAGATCCCGCCAAACCTTCCGTGCTCAGTCACATTGCAACCTGGGCCTGAGGACACAGGGAAGGCCTGCGGTGTGGATTATGAAGTGAAAGCCTTCTGTGCTGAGAACCTGGAGGAGAAGATCCACAAAAGGAATTCTGTGCGGCTAGTCATCCGGAAGGTTCAATATGCCCCTGAGAGGCCTGGCCCTCAGCCCACGGCTGAGACCACCAGACAGTTCCTCATGTCGGACAAGCCCCTGCACCTTGAGGCATCTCTGGATAAGGAGATCTATTATCATGGAGAACCCATCAGCGTCAATGTCCATGTCACCAACAACACCAACAAGACTGTGAAGAAGATCAAGATCTCGGTGCGCCAGTATGCAGACATCTGTCTCTTCAACACAGCTCAGTACAAGTGCCCAGTGGCCATGGAGGAAGCTGATGATACTGTGGCACCCAGCTCAACATTCTGCAAGGTCTACACACTGACTCCCTTCCTGGCAAACAACAGAGAGAAGCGGGGGCTTGCCCTCGACGGGAAGCTCAAGCATGAAGACACAAATCTGGCTTCCAGCACTCTGTTGCGGGAAGGCGCCAACCGTGAAATCCTGGGTATCATTGTTTCCTACAAAGTCAAAGTGAAGCTGGTGGTGTCCCGGGGCGGCCTGTTGGGAGACCTTGCATCCAGTGACGTGGCTGTGGAACTGCCCTTTACCTTAATGCACCCCAAGCCTAAAGAGGAGCCCCCACATCGGGAAGTTCCAGAGAGCGAGACTCCAGTAGACACCAATCTCATAGAGCTTGACACCAATGATGACGACATTGTGTTTGAGGACTTTGCTCGTCAGCGGCTGAAAGGCATGAAGGATGACAAGGACGAAGAGGATGATGGCACCGGCTCTCCACACCTCAACAACAGATACCCATACGACGTCCCAGACTACGCCTAG

**HA-βarr1 1-320**

ATGGGCGACAAAGGGACACGAGTGTTCAAGAAGGCAAGCCCCAATGGAAAGCTCACCGTCTACCTGGGAAAGCGGGACTTTGTGGACCACATTGACCTGGTGGACCCCGTGGATGGCGTGGTCCTGGTGGATCCTGAGTATCTCAAAGAAAGGCGAGTCTACGTGACACTGACCTGCGCCTTCCGGTATGGCCGGGAAGACCTGGATGTCTTGGGTCTGACTTTTCGCAAAGACCTGTTTGTGGCTAACGTGCAGTCCTTCCCACCGGCCCCTGAGGACAAGAAGCCACTGACTCGGCTACAAGAGCGACTCATCAAGAAGCTGGGCGAGCATGCCTACCCCTTCACCTTTGAGATCCCGCCAAACCTTCCGTGCTCAGTCACATTGCAACCTGGGCCTGAGGACACAGGGAAGGCCTGCGGTGTGGATTATGAAGTGAAAGCCTTCTGTGCTGAGAACCTGGAGGAGAAGATCCACAAAAGGAATTCTGTGCGGCTAGTCATCCGGAAGGTTCAATATGCCCCTGAGAGGCCTGGCCCTCAGCCCACGGCTGAGACCACCAGACAGTTCCTCATGTCGGACAAGCCCCTGCACCTTGAGGCATCTCTGGATAAGGAGATCTATTATCATGGAGAACCCATCAGCGTCAATGTCCATGTCACCAACAACACCAACAAGACTGTGAAGAAGATCAAGATCTCGGTGCGCCAGTATGCAGACATCTGTCTCTTCAACACAGCTCAGTACAAGTGCCCAGTGGCCATGGAGGAAGCTGATGATACTGTGGCACCCAGCTCAACATTCTGCAAGGTCTACACACTGACTCCCTTCCTGGCAAACAACAGAGAGAAGCGGGGGCTTGCCCTCGACGGGAAGCTCAAGCATGAAGACACAAATCTGGCTTCCAGCACTCTGTTGCGGGAAGGCGCCAACCGTGAAATCCTGGGTATCATTGTTTCCTACCCATACGACGTCCCAGACTACGCCTAG

**HA-βarr1 1-367**

ATGGGCGACAAAGGGACACGAGTGTTCAAGAAGGCAAGCCCCAATGGAAAGCTCACCGTCTACCTGGGAAAGCGGGACTTTGTGGACCACATTGACCTGGTGGACCCCGTGGATGGCGTGGTCCTGGTGGATCCTGAGTATCTCAAAGAAAGGCGAGTCTACGTGACACTGACCTGCGCCTTCCGGTATGGCCGGGAAGACCTGGATGTCTTGGGTCTGACTTTTCGCAAAGACCTGTTTGTGGCTAACGTGCAGTCCTTCCCACCGGCCCCTGAGGACAAGAAGCCACTGACTCGGCTACAAGAGCGACTCATCAAGAAGCTGGGCGAGCATGCCTACCCCTTCACCTTTGAGATCCCGCCAAACCTTCCGTGCTCAGTCACATTGCAACCTGGGCCTGAGGACACAGGGAAGGCCTGCGGTGTGGATTATGAAGTGAAAGCCTTCTGTGCTGAGAACCTGGAGGAGAAGATCCACAAAAGGAATTCTGTGCGGCTAGTCATCCGGAAGGTTCAATATGCCCCTGAGAGGCCTGGCCCTCAGCCCACGGCTGAGACCACCAGACAGTTCCTCATGTCGGACAAGCCCCTGCACCTTGAGGCATCTCTGGATAAGGAGATCTATTATCATGGAGAACCCATCAGCGTCAATGTCCATGTCACCAACAACACCAACAAGACTGTGAAGAAGATCAAGATCTCGGTGCGCCAGTATGCAGACATCTGTCTCTTCAACACAGCTCAGTACAAGTGCCCAGTGGCCATGGAGGAAGCTGATGATACTGTGGCACCCAGCTCAACATTCTGCAAGGTCTACACACTGACTCCCTTCCTGGCAAACAACAGAGAGAAGCGGGGGCTTGCCCTCGACGGGAAGCTCAAGCATGAAGACACAAATCTGGCTTCCAGCACTCTGTTGCGGGAAGGCGCCAACCGTGAAATCCTGGGTATCATTGTTTCCTACAAAGTCAAAGTGAAGCTGGTGGTGTCCCGGGGCGGCCTGTTGGGAGACCTTGCATCCAGTGACGTGGCTGTGGAACTGCCCTTTACCTTAATGCACCCCAAGCCTAAAGAGGAGCCCCCACATCGGGAAGTTCCAGAGTACCCATACGACGTCCCAGACTACGCCTAG

**HA-βarr1 1-385**

ATGGGCGACAAAGGGACACGAGTGTTCAAGAAGGCAAGCCCCAATGGAAAGCTCACCGTCTACCTGGGAAAGCGGGACTTTGTGGACCACATTGACCTGGTGGACCCCGTGGATGGCGTGGTCCTGGTGGATCCTGAGTATCTCAAAGAAAGGCGAGTCTACGTGACACTGACCTGCGCCTTCCGGTATGGCCGGGAAGACCTGGATGTCTTGGGTCTGACTTTTCGCAAAGACCTGTTTGTGGCTAACGTGCAGTCCTTCCCACCGGCCCCTGAGGACAAGAAGCCACTGACTCGGCTACAAGAGCGACTCATCAAGAAGCTGGGCGAGCATGCCTACCCCTTCACCTTTGAGATCCCGCCAAACCTTCCGTGCTCAGTCACATTGCAACCTGGGCCTGAGGACACAGGGAAGGCCTGCGGTGTGGATTATGAAGTGAAAGCCTTCTGTGCTGAGAACCTGGAGGAGAAGATCCACAAAAGGAATTCTGTGCGGCTAGTCATCCGGAAGGTTCAATATGCCCCTGAGAGGCCTGGCCCTCAGCCCACGGCTGAGACCACCAGACAGTTCCTCATGTCGGACAAGCCCCTGCACCTTGAGGCATCTCTGGATAAGGAGATCTATTATCATGGAGAACCCATCAGCGTCAATGTCCATGTCACCAACAACACCAACAAGACTGTGAAGAAGATCAAGATCTCGGTGCGCCAGTATGCAGACATCTGTCTCTTCAACACAGCTCAGTACAAGTGCCCAGTGGCCATGGAGGAAGCTGATGATACTGTGGCACCCAGCTCAACATTCTGCAAGGTCTACACACTGACTCCCTTCCTGGCAAACAACAGAGAGAAGCGGGGGCTTGCCCTCGACGGGAAGCTCAAGCATGAAGACACAAATCTGGCTTCCAGCACTCTGTTGCGGGAAGGCGCCAACCGTGAAATCCTGGGTATCATTGTTTCCTACAAAGTCAAAGTGAAGCTGGTGGTGTCCCGGGGCGGCCTGTTGGGAGACCTTGCATCCAGTGACGTGGCTGTGGAACTGCCCTTTACCTTAATGCACCCCAAGCCTAAAGAGGAGCCCCCACATCGGGAAGTTCCAGAGAGCGAGACTCCAGTAGACACCAATCTCATAGAGCTTGACACCAATGATGACGACTACCCATACGACGTCCCAGACTACGCCTAG

**βarr1-RFP**

ATGGGCGACAAAGGGACACGAGTGTTCAAGAAGGCAAGCCCCAATGGAAAGCTCACCGTCTACCTGGGAAAGCGGGACTTTGTGGACCACATTGACCTGGTGGACCCCGTGGATGGCGTGGTCCTGGTGGATCCTGAGTATCTCAAAGAAAGGCGAGTCTACGTGACACTGACCTGCGCCTTCCGGTATGGCCGGGAAGACCTGGATGTCTTGGGTCTGACTTTTCGCAAAGACCTGTTTGTGGCTAACGTGCAGTCCTTCCCACCGGCCCCTGAGGACAAGAAGCCACTGACTCGGCTACAAGAGCGACTCATCAAGAAGCTGGGCGAGCATGCCTACCCCTTCACCTTTGAGATCCCGCCAAACCTTCCGTGCTCAGTCACATTGCAACCTGGGCCTGAGGACACAGGGAAGGCCTGCGGTGTGGATTATGAAGTGAAAGCCTTCTGTGCTGAGAACCTGGAGGAGAAGATCCACAAAAGGAATTCTGTGCGGCTAGTCATCCGGAAGGTTCAATATGCCCCTGAGAGGCCTGGCCCTCAGCCCACGGCTGAGACCACCAGACAGTTCCTCATGTCGGACAAGCCCCTGCACCTTGAGGCATCTCTGGATAAGGAGATCTATTATCATGGAGAACCCATCAGCGTCAATGTCCATGTCACCAACAACACCAACAAGACTGTGAAGAAGATCAAGATCTCGGTGCGCCAGTATGCAGACATCTGTCTCTTCAACACAGCTCAGTACAAGTGCCCAGTGGCCATGGAGGAAGCTGATGATACTGTGGCACCCAGCTCAACATTCTGCAAGGTCTACACACTGACTCCCTTCCTGGCAAACAACAGAGAGAAGCGGGGGCTTGCCCTCGACGGGAAGCTCAAGCATGAAGACACAAATCTGGCTTCCAGCACTCTGTTGCGGGAAGGCGCCAACCGTGAAATCCTGGGTATCATTGTTTCCTACAAAGTCAAAGTGAAGCTGGTGGTGTCCCGGGGCGGCCTGTTGGGAGACCTTGCATCCAGTGACGTGGCTGTGGAACTGCCCTTTACCTTAATGCACCCCAAGCCTAAAGAGGAGCCCCCACATCGGGAAGTTCCAGAGAGCGAGACTCCAGTAGACACCAATCTCATAGAGCTTGACACCAATGATGACGACATTGTGTTTGAGGACTTTGCTCGTCAGCGGCTGAAAGGCATGAAGGATGACAAGGACGAAGAGGATGATGGCACCGGCTCTCCACACCTCAACAACAGACTCGAGGCCTCCTCCGAGGACGTCATCAAGGAGTTCATGCGCTTCAAGGTGCGCATGGAGGGCTCCGTGAACGGCCACGAGTTCGAGATCGAGGGCGAGGGCGAGGGCCGCCCCTACGAGGGCACCCAGACCGCCAAGCTGAAGGTGACCAAGGGCGGCCCCCTGCCCTTCGCCTGGGACATCCTGTCCCCTCAGTTCCAGTACGGCTCCAAGGCCTACGTGAAGCACCCCGCCGACATCCCCGACTACTTGAAGCTGTCCTTCCCCGAGGGCTTCAAGTGGGAGCGCGTGATGAACTTCGAGGACGGCGGCGTGGTGACCGCGACCCAGGACTCCTCCCTGCAGGACGGCGAGTTCATCTACAAGGTGAAGCTGCGCGGCACCAACTTCCCCTCCGACGGCCCCGTAATGCAGAAGAAGACCATGGGCTGGGAGGCCTCCACCGAGCGGATGTACCCCGAGGACGGCGCCCTGAAGGGCGAGATCAAGATGAGGCTGAAGCTGAAGGACGGCGGCCACTACGACGCCGAGGTCAAGACCACCTACATGGCCAAGAAGCCCGTGCAGCTGCCCGGCGCCTACAAGACCGACATCAAGCTGGACATCACCTCCCACAACGAGGACTACACCATCGTGGAACAGTACGAGCGCGCCGAGGGCCGCCACTCCACCGGCGCCTAA

**Luc-βarr1**

atgaccagcaaggtgtacgaccccgagcagaggaagaggatgatcaccggcccccagtggtgggccaggtgcaagcagatgaacgtgctggacagcttcatcaactactacgacagcgagaagcacgccgagaacgccgtgatcttcctgcacggcaacgccgctagcagctacctgtggaggcacgtggtgccccacatcgagcccgtggccaggtgcatcatccccgatctgatcggcatgggcaagagcggcaagagcggcaacggcagctacaggctgctggaccactacaagtacctgaccgcctggttcgagctcctgaacctgcccaagaagatcatcttcgtgggccacgactggggcgcctgcctggccttccactacagctacgagcaccaggacaagatcaaggccatcgtgcacgccgagagcgtggtggacgtgatcgagagctgggacgagtggccagacatcgaggaggacatcgccctgatcaagagcgaggagggcgagaagatggtgctggagaacaacttcttcgtggagaccatgctgcccagcaagatcatgagaaagctggagcccgaggagttcgccgcctacctggagcccttcaaggagaagggcgaggtgagaagacccaccctgagctggcccagagagatccccctggtgaagggcggcaagcccgacgtggtgcagatcgtgagaaactacaacgcctacctgagagccagcgacgacctgcccaagatgttcatcgagagcgaccccggcttcttcagcaacgccatcgtggagggcgccaagaagttccccaacaccgagttcgtgaaggtgaagggcctgcacttcagccaggaggacgcccccgacgagatgggcaagtacatcaagagcttcgtggagagagtgctgaagaacgagcagggggatctccggcgagctctcgagaattctcacgcgtctgcaggatatcaagcttgcggtaccggatccATGGGCGACAAAGGGACACGAGTGTTCAAGAAGGCAAGCCCCAATGGAAAGCTCACCGTCTACCTGGGAAAGCGGGACTTTGTGGACCACATTGACCTGGTGGACCCCGTGGATGGCGTGGTCCTGGTGGATCCTGAGTATCTCAAAGAAAGGCGAGTCTACGTGACACTGACCTGCGCCTTCCGGTATGGCCGGGAAGACCTGGATGTCTTGGGTCTGACTTTTCGCAAAGACCTGTTTGTGGCTAACGTGCAGTCCTTCCCACCGGCCCCTGAGGACAAGAAGCCACTGACTCGGCTACAAGAGCGACTCATCAAGAAGCTGGGCGAGCATGCCTACCCCTTCACCTTTGAGATCCCGCCAAACCTTCCGTGCTCAGTCACATTGCAACCTGGGCCTGAGGACACAGGGAAGGCCTGCGGTGTGGATTATGAAGTGAAAGCCTTCTGTGCTGAGAACCTGGAGGAGAAGATCCACAAAAGGAATTCTGTGCGGCTAGTCATCCGGAAGGTTCAATATGCCCCTGAGAGGCCTGGCCCTCAGCCCACGGCTGAGACCACCAGACAGTTCCTCATGTCGGACAAGCCCCTGCACCTTGAGGCATCTCTGGATAAGGAGATCTATTATCATGGAGAACCCATCAGCGTCAATGTCCATGTCACCAACAACACCAACAAGACTGTGAAGAAGATCAAGATCTCGGTGCGCCAGTATGCAGACATCTGTCTCTTCAACACAGCTCAGTACAAGTGCCCAGTGGCCATGGAGGAAGCTGATGATACTGTGGCACCCAGCTCAACATTCTGCAAGGTCTACACACTGACTCCCTTCCTGGCAAACAACAGAGAGAAGCGGGGGCTTGCCCTCGACGGGAAGCTCAAGCATGAAGACACAAATCTGGCTTCCAGCACTCTGTTGCGGGAAGGCGCCAACCGTGAAATCCTGGGTATCATTGTTTCCTACAAAGTCAAAGTGAAGCTGGTGGTGTCCCGGGGCGGCCTGTTGGGAGACCTTGCATCCAGTGACGTGGCTGTGGAACTGCCCTTTACCTTAATGCACCCCAAGCCTAAAGAGGAGCCCCCACATCGGGAAGTTCCAGAGAGCGAGACTCCAGTAGACACCAATCTCATAGAGCTTGACACCAATGATGACGACATTGTGTTTGAGGACTTTGCTCGTCAGCGGCTGAAAGGCATGAAGGATGACAAGGACGAAGAGGATGATGGCACCGGCTCTCCACACCTCAACAACAGATAA

**Luc-βarr2**

atgaccagcaaggtgtacgaccccgagcagaggaagaggatgatcaccggcccccagtggtgggccaggtgcaagcagatgaacgtgctggacagcttcatcaactactacgacagcgagaagcacgccgagaacgccgtgatcttcctgcacggcaacgccgctagcagctacctgtggaggcacgtggtgccccacatcgagcccgtggccaggtgcatcatccccgatctgatcggcatgggcaagagcggcaagagcggcaacggcagctacaggctgctggaccactacaagtacctgaccgcctggttcgagctcctgaacctgcccaagaagatcatcttcgtgggccacgactggggcgcctgcctggccttccactacagctacgagcaccaggacaagatcaaggccatcgtgcacgccgagagcgtggtggacgtgatcgagagctgggacgagtggccagacatcgaggaggacatcgccctgatcaagagcgaggagggcgagaagatggtgctggagaacaacttcttcgtggagaccatgctgcccagcaagatcatgagaaagctggagcccgaggagttcgccgcctacctggagcccttcaaggagaagggcgaggtgagaagacccaccctgagctggcccagagagatccccctggtgaagggcggcaagcccgacgtggtgcagatcgtgagaaactacaacgcctacctgagagccagcgacgacctgcccaagatgttcatcgagagcgaccccggcttcttcagcaacgccatcgtggagggcgccaagaagttccccaacaccgagttcgtgaaggtgaagggcctgcacttcagccaggaggacgcccccgacgagatgggcaagtacatcaagagcttcgtggagagagtgctgaagaacgagcagggggatctccggcgagctctcgagaattctcacgcgtctgcaggatatcaagcttgcggtaccggatccATGGGGGAGAAACCCGGGACCAGGGTCTTCAAGAAGTCGAGCCCTAACTGCAAGCTCACCGTGTACTTGGGCAAGCGGGACTTCGTAGATCACCTGGACAAAGTGGACCCTGTAGATGGCGTGGTGCTTGTGGACCCTGACTACCTGAAGGACCGCAAAGTGTTTGTGACCCTCACCTGCGCCTTCCGCTATGGCCGTGAAGACCTGGATGTGCTGGGCTTGTCCTTCCGCAAAGACCTGTTCATCGCCACCTACCAGGCCTTCCCCCCGGTGCCCAACCCACCCCGGCCCCCCACCCGCCTGCAGGACCGGCTGCTGAGGAAGCTGGGCCAGCATGCCCACCCCTTCTTCTTCACCATACCCCAGAATCTTCCATGCTCCGTCACACTGCAGCCAGGCCCAGAGGATACAGGAAAGGCCTGCGGCGTAGACTTTGAGATTCGAGCCTTCTGTGCTAAATCACTAGAAGAGAAAAGCCACAAAAGGAACTCTGTGCGGCTGGTGATCCGAAAGGTGCAGTTCGCCCCGGAGAAACCCGGCCCCCAGCCTTCAGCCGAAACCACACGCCACTTCCTCATGTCTGACCGGTCCCTGCACCTCGAGGCTTCCCTGGACAAGGAGCTGTACTACCATGGGGAGCCCCTCAATGTAAATGTCCACGTCACCAACAACTCCACCAAGACCGTCAAGAAGATCAAAGTCTCTGTGAGACAGTACGCCGACATCTGCCTCTTCAGCACCGCCCAGTACAAGTGTCCTGTGGCTCAACTCGAACAAGATGACCAGGTATCTCCCAGCTCCACATTCTGTAAGGTGTACACCATAACCCCACTGCTCAGTGACAACCGGGAGAAGCGGGGTCTCGCCCTGGATGGGAAACTCAAGCACGAGGACACCAACCTGGCTTCCAGCACCATCGTGAAGGAGGGTGCCAACAAGGAGGTGCTGGGAATCCTGGTGTCCTACAGGGTCAAGGTGAAGCTGGTGGTGTCTCGAGGCGGGGATGTCTCTGTGGAGCTGCCTTTTGTTCTTATGCACCCCAAGCCCCACGACCACATCCCCCTCCCCAGACCCCAGTCAGCCGCTCCGGAGACAGATGTCCCTGTGGACACCAACCTCATTGAATTTGATACCAACTATGCCACAGATGATGACATTGTGTTTGAGGACTTTGCCCGGCTTCGGCTGAAGGGGATGAAGGATGACGACTATGATGATCAACTCTGCTAA

**HA-βarr1_N_-βarr2_C_**

ATGGGCGACAAAGGGACACGAGTGTTCAAGAAGGCAAGCCCCAATGGAAAGCTCACCGTCTACCTGGGAAAGCGGGACTTTGTGGACCACATTGACCTGGTGGACCCCGTGGATGGCGTGGTCCTGGTGGATCCTGAGTATCTCAAAGAAAGGCGAGTCTACGTGACACTGACCTGCGCCTTCCGGTATGGCCGGGAAGACCTGGATGTCTTGGGTCTGACTTTTCGCAAAGACCTGTTTGTGGCTAACGTGCAGTCCTTCCCACCGGCCCCTGAGGACAAGAAGCCACTGACTCGGCTACAAGAGCGACTCATCAAGAAGCTGGGCGAGCATGCCTACCCCTTCACCTTTGAGATCCCGCCAAACCTTCCGTGCTCAGTCACATTGCAACCTGGGCCTGAGGACACAGGGAAGGCCTGCGGTGTGGATTATGAAGTGAAAGCCTTCTGTGCTGAGAACCTGGAGGAGAAGATCCACAAAAGGAATTCTGTGCGGCTAGTCATCCGGAAGGTTCAATATGCCCCTGAGAGGCCTGGCCCTCAGCCCACGGCTGAGACCACCAGACAGTTCCTCATGTCGGACAAGCCCCTGCACCTTGAGGCATCTCTGGATAAGGAGATCTATTATCATGGAGAACCCATCAGCGTCAATGTCCATGTCACCAACAACACCAACAAGACTGTGAAGAAGATCAAGATCTCGGTGCGCCAGTATGCAGACATCTGTCTCTTCAACACAGCTCAGTACAAGTGCCCAGTGGCCATGGAGGAAGCTGATGATACTGTGGCACCCAGCTCAACATTCTGCAAGGTCTACACACTGACTCCCTTCCTGGCAAACAACAGAGAGAAGCGGGGGCTTGCCCTCGACGGGAAGCTCAAGCATGAAGACACAAATCTGGCTTCCAGCACTCTGTTGCGGGAAGGCGCCAACCGTGAAATCCTGGGTATCATTGTTTCCTCCTACAGGGTCAAGGTGAAGCTGGTGGTGTCTCGAGGCGGGGATGTCTCTGTGGAGCTGCCTTTTGTTCTTATGCACCCCAAGCCCCACGACCACATCCCCCTCCCCAGACCCCAGTCAGCCGCTCCGGAGACAGATGTCCCTGTGGACACCAACCTCATTGAATTTGATACCAACTATGCCACAGATGATGACATTGTGTTTGAGGACTTTGCCCGGCTTCGGCTGAAGGGGATGAAGGATGACGACTATGATGATCAACTCTGCTACCCATACGACGTCCCAGACTACGCCTAG

**HA-βarr2_N_-βarr1_C_**

atgggggagaaacccgggaccagggtcttcaagaagtcgagccctaactgcaagctcaccgtgtacttgggcaagcgggacttcgtagatcacctggacaaagtggaccctgtagatggcgtggtgcttgtggaccctgactacctgaaggaccgcaaagtgtttgtgaccctcacctgcgccttccgctatggccgtgaagacctggatgtgctgggcttgtccttccgcaaagacctgttcatcgccacctaccaggccttccccccggtgcccaacccaccccggccccccacccgcctgcaggaccggctgctgaggaagctgggccagcatgcccaccccttcttcttcaccataccccagaatcttccatgctccgtcacactgcagccaggcccagaggatacaggaaaggcctgcggcgtagactttgagattcgagccttctgtgctaaatcactagaagagaaaagccacaaaaggaactctgtgcggctggtgatccgaaaggtgcagttcgccccggagaaacccggcccccagccttcagccgaaaccacacgccacttcctcatgtctgaccggtccctgcacctcgaggcttccctggacaaggagctgtactaccatggggagcccctcaatgtaaatgtccacgtcaccaacaactccaccaagaccgtcaagaagatcaaagtctctgtgagacagtacgccgacatctgcctcttcagcaccgcccagtacaagtgtcctgtggctcaactcgaacaagatgaccaggtatctcccagctccacattctgtaaggtgtacaccataaccccactgctcagtgacaaccgggagaagcggggtctcgccctggatgggaaactcaagcacgaggacaccaacctggcttccagcaccatcgtgaaggagggtgccaacaaggaggtgctgggaatcctggtgtccTCCTACAAAGTCAAAGTGAAGCTGGTGGTGTCCCGGGGCGGCCTGTTGGGAGACCTTGCATCCAGTGACGTGGCTGTGGAACTGCCCTTTACCTTAATGCACCCCAAGCCTAAAGAGGAGCCCCCACATCGGGAAGTTCCAGAGAGCGAGACTCCAGTAGACACCAATCTCATAGAGCTTGACACCAATGATGACGACATTGTGTTTGAGGACTTTGCTCGTCAGCGGCTGAAAGGCATGAAGGATGACAAGGACGAAGAGGATGATGGCACCGGCTCTCCACACCTCAACAACAGATACCCATACGACGTCCCAGACTACGCCTAG

**TRPC3-GFP-WT**

ATGGAGGGAAGCCCATCCCTGAGACGCATGACAGTGATGCGGGAGAAGGGCCGGCGCCAGGCTGTCAGGGGCCCGGCCTTCATGTTCAATGACCGCGGCACCAGCCTCACCGCCGAGGAGGAGCGCTTCCTCGACGCCGCCGAGTACGGCAACATCCCAGTGGTGCGCAAGATGCTGGAGGAGTCCAAGACGCTGAACGTCAACTGCGTGGACTACATGGGCCAGAACGCGCTGCAGCTGGCTGTGGGCAACGAGCACCTGGAGGTGACCGAGCTGCTGCTCAAGAAGGAGAACCTGGCGCGCATTGGCGACGCCCTGCTGCTCGCCATCAGCAAGGGCTACGTGCGCATCGTAGAGGCCATCCTCAACCACCCTGGCTTCGCGGCCAGCAAGCGTCTCACTCTGAGCCCCTGTGAGCAGGAGCTGCAGGACGACGACTTCTACGCTTACGACGAGGACGGCACGCGCTTCTCGCCGGACATCACCCCCATCATCCTGGCGGCGCACTGCCAGAAATACGAAGTGGTGCACATGCTGCTGATGAAGGGTGCCAGGATCGAGCGGCCGCACGACTATTTCTGCAAGTGCGGGGACTGCATGGAGAAGCAGAGGCACGACTCCTTCAGCCACTCACGCTCGAGGATCAATGCCTACAAGGGGCTGGCCAGCCCGGCTTACCTCTCATTGTCCAGCGAGGACCCGGTGCTTACGGCCCTAGAGCTCAGCAACGAGCTGGCCAAGCTGGCCAACATAGAGAAGGAGTTCAAGAATGACTATCGGAAGCTCTCCATGCAATGCAAAGACTTTGTAGTGGGTGTGCTGGATCTCTGCCGAGACTCAGAAGAGGTAGAAGCCATTCTGAATGGAGATCTGGAATCAGCAGAGCCTCTGGAGGTACACAGGCACAAAGCTTCATTAAGTCGTGTCAAACTTGCCATTAAGTATGAAGTCAAAAAGTTTGTGGCTCATCCCAACTGCCAGCAGCAGCTCTTGACGATCTGGTATGAGAACCTCTCAGGCCTAAGGGAGCAGACCATAGCTATCAAGTGTCTCGTTGTGCTGGTCGTGGCCCTGGGCCTTCCATTCCTGGCCATTGGCTACTGGATCGCACCTTGCAGCAGGCTGGGGAAAATTCTGCGAAGCCCTTTTATGAAGTTTGTAGCACATGCAGCTTCTTTCATCATCTTCCTGGGTCTGCTTGTGTTCAATGCCTCAGACAGGTTCGAAGGCATCACCACGCTGCCCAATATCACAGTTACTGACTATCCCAAACAGATCTTCAGGGTGAAAACCACCCAGTTTACATGGACTGAAATGCTAATTATGGTCTGGGTTCTTGGAATGATGTGGTCTGAATGTAAAGAGCTCTGGCTGGAAGGACCTAGGGAATACATTTTGCAGTTGTGGAATGTGCTTGACTTTGGGATGCTGTCCATCTTCATTGCTGCTTTCACAGCCAGATTCCTAGCTTTCCTTCAGGCAACGAAGGCACAACAGTATGTGGACAGTTACGTCCAAGAGAGTGACCTCAGTGAAGTGACACTCCCACCAGAGATACAGTATTTCACTTATGCTAGAGATAAATGGCTCCCTTCTGACCCTCAGATTATATCTGAAGGCCTTTATGCCATAGCTGTTGTGCTCAGCTTCTCTCGGATTGCGTACATCCTCCCTGCAAATGAGAGCTTTGGCCCCCTGCAGATCTCTCTTGGAAGGACTGTAAAGGACATATTCAAGTTCATGGTCCTCTTTATTATGGTGTTTTTTGCCTTTATGATTGGCATGTTCATACTTTATTCTTACTACCTTGGGGCTAAAGTTAATGCTGCTTTTACCACTGTAGAAGAAAGTTTCAAGACTTTATTTTGGTCAATATTTGGGTTGTCTGAAGTGACTTCCGTTGTGCTCAAATATGATCACAAATTCATAGAAAATATTGGATACGTTCTTTATGGAATATACAATGTAACTATGGTGGTCGTTTTACTCAACATGCTAATTGCTATGATTAATAGCTCATATCAAGAAATTGAGGATGACAGTGATGTAGAATGGAAGTTTGCTCGTTCAAAACTTTGGTTATCCTATTTTGATGATGGAAAAACATTACCTCCACCTTTCAGTCTAGTTCCTAGTCCAAAATCATTTGTTTATTTCATCATGCGAATTGTTAACTTTCCCAAATGCAGAAGGAGAAGGCTTCAGAAGGATATAGAAATGGGAATGGGTAACTCAAAGTCCAGGTTAAACCTCTTCACTCAGTCTAACTCAAGAGTTTTTGAATCACACAGTTTTAACAGCATTCTCAATCAGCCAACACGTTATCAGCAGATAATGAAAAGACTTATAAAGCGGTATGTTTTGAAAGCACAAGTAGACAAAGAAAATGATGAAGTTAATGAAGGTGAATTAAAAGAAATCAAGCAAGATATCTCCAGCCTTCGTTATGAACTTTTGGAAGACAAGAGCCAAGCAACTGAGGAATTAGCCATTCTAATTCATAAACTTAGTGAGAAACTGAATCCCAGCATGCTGAGATGTGAAGGCGCGCCTATGGTGAGCAAGGGCGAGGAGCTGTTCACCGGGGTGGTGCCCATCCTGGTCGAGCTGGACGGCGACGTAAACGGCCACAAGTTCAGCGTGTCCGGCGAGGGCGAGGGCGATGCCACCTACGGCAAGCTGACCCTGAAGTTCATCTGCACCACCGGCAAGCTGCCCGTGCCCTGGCCCACCCTCGTGACCACCCTGACCTACGGCGTGCAGTGCTTCAGCCGCTACCCCGACCACATGAAGCAGCACGACTTCTTCAAGTCCGCCATGCCCGAAGGCTACGTCCAGGAGCGCACCATCTTCTTCAAGGACGACGGCAACTACAAGACCCGCGCCGAGGTGAAGTTCGAGGGCGACACCCTGGTGAACCGCATCGAGCTGAAGGGCATCGACTTCAAGGAGGACGGCAACATCCTGGGGCACAAGCTGGAGTACAACTACAACAGCCACAACGTCTATATCATGGCCGACAAGCAGAAGAACGGCATCAAGGTGAACTTCAAGATCCGCCACAACATCGAGGACGGCAGCGTGCAGCTCGCCGACCACTACCAGCAGAACACCCCCATCGGCGACGGCCCCGTGCTGCTGCCCGACAACCACTACCTGAGCACCCAGTCCGCCCTGAGCAAAGACCCCAACGAGAAGCGCGATCACATGGTCCTGCTGGAGTTCGTGACCGCCGCCGGGATCACTCTCGGCATGGACGAGCTGTACAAGTAA

**TRPC3-GFP 73-848**

ATGGGCCAGAACGCGCTGCAGCTGGCTGTGGGCAACGAGCACCTGGAGGTGACCGAGCTGCTGCTCAAGAAGGAGAACCTGGCGCGCATTGGCGACGCCCTGCTGCTCGCCATCAGCAAGGGCTACGTGCGCATCGTAGAGGCCATCCTCAACCACCCTGGCTTCGCGGCCAGCAAGCGTCTCACTCTGAGCCCCTGTGAGCAGGAGCTGCAGGACGACGACTTCTACGCTTACGACGAGGACGGCACGCGCTTCTCGCCGGACATCACCCCCATCATCCTGGCGGCGCACTGCCAGAAATACGAAGTGGTGCACATGCTGCTGATGAAGGGTGCCAGGATCGAGCGGCCGCACGACTATTTCTGCAAGTGCGGGGACTGCATGGAGAAGCAGAGGCACGACTCCTTCAGCCACTCACGCTCGAGGATCAATGCCTACAAGGGGCTGGCCAGCCCGGCTTACCTCTCATTGTCCAGCGAGGACCCGGTGCTTACGGCCCTAGAGCTCAGCAACGAGCTGGCCAAGCTGGCCAACATAGAGAAGGAGTTCAAGAATGACTATCGGAAGCTCTCCATGCAATGCAAAGACTTTGTAGTGGGTGTGCTGGATCTCTGCCGAGACTCAGAAGAGGTAGAAGCCATTCTGAATGGAGATCTGGAATCAGCAGAGCCTCTGGAGGTACACAGGCACAAAGCTTCATTAAGTCGTGTCAAACTTGCCATTAAGTATGAAGTCAAAAAGTTTGTGGCTCATCCCAACTGCCAGCAGCAGCTCTTGACGATCTGGTATGAGAACCTCTCAGGCCTAAGGGAGCAGACCATAGCTATCAAGTGTCTCGTTGTGCTGGTCGTGGCCCTGGGCCTTCCATTCCTGGCCATTGGCTACTGGATCGCACCTTGCAGCAGGCTGGGGAAAATTCTGCGAAGCCCTTTTATGAAGTTTGTAGCACATGCAGCTTCTTTCATCATCTTCCTGGGTCTGCTTGTGTTCAATGCCTCAGACAGGTTCGAAGGCATCACCACGCTGCCCAATATCACAGTTACTGACTATCCCAAACAGATCTTCAGGGTGAAAACCACCCAGTTTACATGGACTGAAATGCTAATTATGGTCTGGGTTCTTGGAATGATGTGGTCTGAATGTAAAGAGCTCTGGCTGGAAGGACCTAGGGAATACATTTTGCAGTTGTGGAATGTGCTTGACTTTGGGATGCTGTCCATCTTCATTGCTGCTTTCACAGCCAGATTCCTAGCTTTCCTTCAGGCAACGAAGGCACAACAGTATGTGGACAGTTACGTCCAAGAGAGTGACCTCAGTGAAGTGACACTCCCACCAGAGATACAGTATTTCACTTATGCTAGAGATAAATGGCTCCCTTCTGACCCTCAGATTATATCTGAAGGCCTTTATGCCATAGCTGTTGTGCTCAGCTTCTCTCGGATTGCGTACATCCTCCCTGCAAATGAGAGCTTTGGCCCCCTGCAGATCTCTCTTGGAAGGACTGTAAAGGACATATTCAAGTTCATGGTCCTCTTTATTATGGTGTTTTTTGCCTTTATGATTGGCATGTTCATACTTTATTCTTACTACCTTGGGGCTAAAGTTAATGCTGCTTTTACCACTGTAGAAGAAAGTTTCAAGACTTTATTTTGGTCAATATTTGGGTTGTCTGAAGTGACTTCCGTTGTGCTCAAATATGATCACAAATTCATAGAAAATATTGGATACGTTCTTTATGGAATATACAATGTAACTATGGTGGTCGTTTTACTCAACATGCTAATTGCTATGATTAATAGCTCATATCAAGAAATTGAGGATGACAGTGATGTAGAATGGAAGTTTGCTCGTTCAAAACTTTGGTTATCCTATTTTGATGATGGAAAAACATTACCTCCACCTTTCAGTCTAGTTCCTAGTCCAAAATCATTTGTTTATTTCATCATGCGAATTGTTAACTTTCCCAAATGCAGAAGGAGAAGGCTTCAGAAGGATATAGAAATGGGAATGGGTAACTCAAAGTCCAGGTTAAACCTCTTCACTCAGTCTAACTCAAGAGTTTTTGAATCACACAGTTTTAACAGCATTCTCAATCAGCCAACACGTTATCAGCAGATAATGAAAAGACTTATAAAGCGGTATGTTTTGAAAGCACAAGTAGACAAAGAAAATGATGAAGTTAATGAAGGTGAATTAAAAGAAATCAAGCAAGATATCTCCAGCCTTCGTTATGAACTTTTGGAAGACAAGAGCCAAGCAACTGAGGAATTAGCCATTCTAATTCATAAACTTAGTGAGAAACTGAATCCCAGCATGCTGAGATGTGAAGGCGCGCCTATGGTGAGCAAGGGCGAGGAGCTGTTCACCGGGGTGGTGCCCATCCTGGTCGAGCTGGACGGCGACGTAAACGGCCACAAGTTCAGCGTGTCCGGCGAGGGCGAGGGCGATGCCACCTACGGCAAGCTGACCCTGAAGTTCATCTGCACCACCGGCAAGCTGCCCGTGCCCTGGCCCACCCTCGTGACCACCCTGACCTACGGCGTGCAGTGCTTCAGCCGCTACCCCGACCACATGAAGCAGCACGACTTCTTCAAGTCCGCCATGCCCGAAGGCTACGTCCAGGAGCGCACCATCTTCTTCAAGGACGACGGCAACTACAAGACCCGCGCCGAGGTGAAGTTCGAGGGCGACACCCTGGTGAACCGCATCGAGCTGAAGGGCATCGACTTCAAGGAGGACGGCAACATCCTGGGGCACAAGCTGGAGTACAACTACAACAGCCACAACGTCTATATCATGGCCGACAAGCAGAAGAACGGCATCAAGGTGAACTTCAAGATCCGCCACAACATCGAGGACGGCAGCGTGCAGCTCGCCGACCACTACCAGCAGAACACCCCCATCGGCGACGGCCCCGTGCTGCTGCCCGACAACCACTACCTGAGCACCCAGTCCGCCCTGAGCAAAGACCCCAACGAGAAGCGCGATCACATGGTCCTGCTGGAGTTCGTGACCGCCGCCGGGATCACTCTCGGCATGGACGAGCTGTACAAGTAA

**TRPC3-GFP 178-848**

ATGCTGCTGATGAAGGGTGCCAGGATCGAGCGGCCGCACGACTATTTCTGCAAGTGCGGGGACTGCATGGAGAAGCAGAGGCACGACTCCTTCAGCCACTCACGCTCGAGGATCAATGCCTACAAGGGGCTGGCCAGCCCGGCTTACCTCTCATTGTCCAGCGAGGACCCGGTGCTTACGGCCCTAGAGCTCAGCAACGAGCTGGCCAAGCTGGCCAACATAGAGAAGGAGTTCAAGAATGACTATCGGAAGCTCTCCATGCAATGCAAAGACTTTGTAGTGGGTGTGCTGGATCTCTGCCGAGACTCAGAAGAGGTAGAAGCCATTCTGAATGGAGATCTGGAATCAGCAGAGCCTCTGGAGGTACACAGGCACAAAGCTTCATTAAGTCGTGTCAAACTTGCCATTAAGTATGAAGTCAAAAAGTTTGTGGCTCATCCCAACTGCCAGCAGCAGCTCTTGACGATCTGGTATGAGAACCTCTCAGGCCTAAGGGAGCAGACCATAGCTATCAAGTGTCTCGTTGTGCTGGTCGTGGCCCTGGGCCTTCCATTCCTGGCCATTGGCTACTGGATCGCACCTTGCAGCAGGCTGGGGAAAATTCTGCGAAGCCCTTTTATGAAGTTTGTAGCACATGCAGCTTCTTTCATCATCTTCCTGGGTCTGCTTGTGTTCAATGCCTCAGACAGGTTCGAAGGCATCACCACGCTGCCCAATATCACAGTTACTGACTATCCCAAACAGATCTTCAGGGTGAAAACCACCCAGTTTACATGGACTGAAATGCTAATTATGGTCTGGGTTCTTGGAATGATGTGGTCTGAATGTAAAGAGCTCTGGCTGGAAGGACCTAGGGAATACATTTTGCAGTTGTGGAATGTGCTTGACTTTGGGATGCTGTCCATCTTCATTGCTGCTTTCACAGCCAGATTCCTAGCTTTCCTTCAGGCAACGAAGGCACAACAGTATGTGGACAGTTACGTCCAAGAGAGTGACCTCAGTGAAGTGACACTCCCACCAGAGATACAGTATTTCACTTATGCTAGAGATAAATGGCTCCCTTCTGACCCTCAGATTATATCTGAAGGCCTTTATGCCATAGCTGTTGTGCTCAGCTTCTCTCGGATTGCGTACATCCTCCCTGCAAATGAGAGCTTTGGCCCCCTGCAGATCTCTCTTGGAAGGACTGTAAAGGACATATTCAAGTTCATGGTCCTCTTTATTATGGTGTTTTTTGCCTTTATGATTGGCATGTTCATACTTTATTCTTACTACCTTGGGGCTAAAGTTAATGCTGCTTTTACCACTGTAGAAGAAAGTTTCAAGACTTTATTTTGGTCAATATTTGGGTTGTCTGAAGTGACTTCCGTTGTGCTCAAATATGATCACAAATTCATAGAAAATATTGGATACGTTCTTTATGGAATATACAATGTAACTATGGTGGTCGTTTTACTCAACATGCTAATTGCTATGATTAATAGCTCATATCAAGAAATTGAGGATGACAGTGATGTAGAATGGAAGTTTGCTCGTTCAAAACTTTGGTTATCCTATTTTGATGATGGAAAAACATTACCTCCACCTTTCAGTCTAGTTCCTAGTCCAAAATCATTTGTTTATTTCATCATGCGAATTGTTAACTTTCCCAAATGCAGAAGGAGAAGGCTTCAGAAGGATATAGAAATGGGAATGGGTAACTCAAAGTCCAGGTTAAACCTCTTCACTCAGTCTAACTCAAGAGTTTTTGAATCACACAGTTTTAACAGCATTCTCAATCAGCCAACACGTTATCAGCAGATAATGAAAAGACTTATAAAGCGGTATGTTTTGAAAGCACAAGTAGACAAAGAAAATGATGAAGTTAATGAAGGTGAATTAAAAGAAATCAAGCAAGATATCTCCAGCCTTCGTTATGAACTTTTGGAAGACAAGAGCCAAGCAACTGAGGAATTAGCCATTCTAATTCATAAACTTAGTGAGAAACTGAATCCCAGCATGCTGAGATGTGAAGGCGCGCCTATGGTGAGCAAGGGCGAGGAGCTGTTCACCGGGGTGGTGCCCATCCTGGTCGAGCTGGACGGCGACGTAAACGGCCACAAGTTCAGCGTGTCCGGCGAGGGCGAGGGCGATGCCACCTACGGCAAGCTGACCCTGAAGTTCATCTGCACCACCGGCAAGCTGCCCGTGCCCTGGCCCACCCTCGTGACCACCCTGACCTACGGCGTGCAGTGCTTCAGCCGCTACCCCGACCACATGAAGCAGCACGACTTCTTCAAGTCCGCCATGCCCGAAGGCTACGTCCAGGAGCGCACCATCTTCTTCAAGGACGACGGCAACTACAAGACCCGCGCCGAGGTGAAGTTCGAGGGCGACACCCTGGTGAACCGCATCGAGCTGAAGGGCATCGACTTCAAGGAGGACGGCAACATCCTGGGGCACAAGCTGGAGTACAACTACAACAGCCACAACGTCTATATCATGGCCGACAAGCAGAAGAACGGCATCAAGGTGAACTTCAAGATCCGCCACAACATCGAGGACGGCAGCGTGCAGCTCGCCGACCACTACCAGCAGAACACCCCCATCGGCGACGGCCCCGTGCTGCTGCCCGACAACCACTACCTGAGCACCCAGTCCGCCCTGAGCAAAGACCCCAACGAGAAGCGCGATCACATGGTCCTGCTGGAGTTCGTGACCGCCGCCGGGATCACTCTCGGCATGGACGAGCTGTACAAGTAA

**TRPC3-GFP 334-848**

ATGTGGTATGAGAACCTCTCAGGCCTAAGGGAGCAGACCATAGCTATCAAGTGTCTCGTTGTGCTGGTCGTGGCCCTGGGCCTTCCATTCCTGGCCATTGGCTACTGGATCGCACCTTGCAGCAGGCTGGGGAAAATTCTGCGAAGCCCTTTTATGAAGTTTGTAGCACATGCAGCTTCTTTCATCATCTTCCTGGGTCTGCTTGTGTTCAATGCCTCAGACAGGTTCGAAGGCATCACCACGCTGCCCAATATCACAGTTACTGACTATCCCAAACAGATCTTCAGGGTGAAAACCACCCAGTTTACATGGACTGAAATGCTAATTATGGTCTGGGTTCTTGGAATGATGTGGTCTGAATGTAAAGAGCTCTGGCTGGAAGGACCTAGGGAATACATTTTGCAGTTGTGGAATGTGCTTGACTTTGGGATGCTGTCCATCTTCATTGCTGCTTTCACAGCCAGATTCCTAGCTTTCCTTCAGGCAACGAAGGCACAACAGTATGTGGACAGTTACGTCCAAGAGAGTGACCTCAGTGAAGTGACACTCCCACCAGAGATACAGTATTTCACTTATGCTAGAGATAAATGGCTCCCTTCTGACCCTCAGATTATATCTGAAGGCCTTTATGCCATAGCTGTTGTGCTCAGCTTCTCTCGGATTGCGTACATCCTCCCTGCAAATGAGAGCTTTGGCCCCCTGCAGATCTCTCTTGGAAGGACTGTAAAGGACATATTCAAGTTCATGGTCCTCTTTATTATGGTGTTTTTTGCCTTTATGATTGGCATGTTCATACTTTATTCTTACTACCTTGGGGCTAAAGTTAATGCTGCTTTTACCACTGTAGAAGAAAGTTTCAAGACTTTATTTTGGTCAATATTTGGGTTGTCTGAAGTGACTTCCGTTGTGCTCAAATATGATCACAAATTCATAGAAAATATTGGATACGTTCTTTATGGAATATACAATGTAACTATGGTGGTCGTTTTACTCAACATGCTAATTGCTATGATTAATAGCTCATATCAAGAAATTGAGGATGACAGTGATGTAGAATGGAAGTTTGCTCGTTCAAAACTTTGGTTATCCTATTTTGATGATGGAAAAACATTACCTCCACCTTTCAGTCTAGTTCCTAGTCCAAAATCATTTGTTTATTTCATCATGCGAATTGTTAACTTTCCCAAATGCAGAAGGAGAAGGCTTCAGAAGGATATAGAAATGGGAATGGGTAACTCAAAGTCCAGGTTAAACCTCTTCACTCAGTCTAACTCAAGAGTTTTTGAATCACACAGTTTTAACAGCATTCTCAATCAGCCAACACGTTATCAGCAGATAATGAAAAGACTTATAAAGCGGTATGTTTTGAAAGCACAAGTAGACAAAGAAAATGATGAAGTTAATGAAGGTGAATTAAAAGAAATCAAGCAAGATATCTCCAGCCTTCGTTATGAACTTTTGGAAGACAAGAGCCAAGCAACTGAGGAATTAGCCATTCTAATTCATAAACTTAGTGAGAAACTGAATCCCAGCATGCTGAGATGTGAAGGCGCGCCTATGGTGAGCAAGGGCGAGGAGCTGTTCACCGGGGTGGTGCCCATCCTGGTCGAGCTGGACGGCGACGTAAACGGCCACAAGTTCAGCGTGTCCGGCGAGGGCGAGGGCGATGCCACCTACGGCAAGCTGACCCTGAAGTTCATCTGCACCACCGGCAAGCTGCCCGTGCCCTGGCCCACCCTCGTGACCACCCTGACCTACGGCGTGCAGTGCTTCAGCCGCTACCCCGACCACATGAAGCAGCACGACTTCTTCAAGTCCGCCATGCCCGAAGGCTACGTCCAGGAGCGCACCATCTTCTTCAAGGACGACGGCAACTACAAGACCCGCGCCGAGGTGAAGTTCGAGGGCGACACCCTGGTGAACCGCATCGAGCTGAAGGGCATCGACTTCAAGGAGGACGGCAACATCCTGGGGCACAAGCTGGAGTACAACTACAACAGCCACAACGTCTATATCATGGCCGACAAGCAGAAGAACGGCATCAAGGTGAACTTCAAGATCCGCCACAACATCGAGGACGGCAGCGTGCAGCTCGCCGACCACTACCAGCAGAACACCCCCATCGGCGACGGCCCCGTGCTGCTGCCCGACAACCACTACCTGAGCACCCAGTCCGCCCTGAGCAAAGACCCCAACGAGAAGCGCGATCACATGGTCCTGCTGGAGTTCGTGACCGCCGCCGGGATCACTCTCGGCATGGACGAGCTGTACAAGTAA

**TRPC3-GFP 1-790**

ATGGAGGGAAGCCCATCCCTGAGACGCATGACAGTGATGCGGGAGAAGGGCCGGCGCCAGGCTGTCAGGGGCCCGGCCTTCATGTTCAATGACCGCGGCACCAGCCTCACCGCCGAGGAGGAGCGCTTCCTCGACGCCGCCGAGTACGGCAACATCCCAGTGGTGCGCAAGATGCTGGAGGAGTCCAAGACGCTGAACGTCAACTGCGTGGACTACATGGGCCAGAACGCGCTGCAGCTGGCTGTGGGCAACGAGCACCTGGAGGTGACCGAGCTGCTGCTCAAGAAGGAGAACCTGGCGCGCATTGGCGACGCCCTGCTGCTCGCCATCAGCAAGGGCTACGTGCGCATCGTAGAGGCCATCCTCAACCACCCTGGCTTCGCGGCCAGCAAGCGTCTCACTCTGAGCCCCTGTGAGCAGGAGCTGCAGGACGACGACTTCTACGCTTACGACGAGGACGGCACGCGCTTCTCGCCGGACATCACCCCCATCATCCTGGCGGCGCACTGCCAGAAATACGAAGTGGTGCACATGCTGCTGATGAAGGGTGCCAGGATCGAGCGGCCGCACGACTATTTCTGCAAGTGCGGGGACTGCATGGAGAAGCAGAGGCACGACTCCTTCAGCCACTCACGCTCGAGGATCAATGCCTACAAGGGGCTGGCCAGCCCGGCTTACCTCTCATTGTCCAGCGAGGACCCGGTGCTTACGGCCCTAGAGCTCAGCAACGAGCTGGCCAAGCTGGCCAACATAGAGAAGGAGTTCAAGAATGACTATCGGAAGCTCTCCATGCAATGCAAAGACTTTGTAGTGGGTGTGCTGGATCTCTGCCGAGACTCAGAAGAGGTAGAAGCCATTCTGAATGGAGATCTGGAATCAGCAGAGCCTCTGGAGGTACACAGGCACAAAGCTTCATTAAGTCGTGTCAAACTTGCCATTAAGTATGAAGTCAAAAAGTTTGTGGCTCATCCCAACTGCCAGCAGCAGCTCTTGACGATCTGGTATGAGAACCTCTCAGGCCTAAGGGAGCAGACCATAGCTATCAAGTGTCTCGTTGTGCTGGTCGTGGCCCTGGGCCTTCCATTCCTGGCCATTGGCTACTGGATCGCACCTTGCAGCAGGCTGGGGAAAATTCTGCGAAGCCCTTTTATGAAGTTTGTAGCACATGCAGCTTCTTTCATCATCTTCCTGGGTCTGCTTGTGTTCAATGCCTCAGACAGGTTCGAAGGCATCACCACGCTGCCCAATATCACAGTTACTGACTATCCCAAACAGATCTTCAGGGTGAAAACCACCCAGTTTACATGGACTGAAATGCTAATTATGGTCTGGGTTCTTGGAATGATGTGGTCTGAATGTAAAGAGCTCTGGCTGGAAGGACCTAGGGAATACATTTTGCAGTTGTGGAATGTGCTTGACTTTGGGATGCTGTCCATCTTCATTGCTGCTTTCACAGCCAGATTCCTAGCTTTCCTTCAGGCAACGAAGGCACAACAGTATGTGGACAGTTACGTCCAAGAGAGTGACCTCAGTGAAGTGACACTCCCACCAGAGATACAGTATTTCACTTATGCTAGAGATAAATGGCTCCCTTCTGACCCTCAGATTATATCTGAAGGCCTTTATGCCATAGCTGTTGTGCTCAGCTTCTCTCGGATTGCGTACATCCTCCCTGCAAATGAGAGCTTTGGCCCCCTGCAGATCTCTCTTGGAAGGACTGTAAAGGACATATTCAAGTTCATGGTCCTCTTTATTATGGTGTTTTTTGCCTTTATGATTGGCATGTTCATACTTTATTCTTACTACCTTGGGGCTAAAGTTAATGCTGCTTTTACCACTGTAGAAGAAAGTTTCAAGACTTTATTTTGGTCAATATTTGGGTTGTCTGAAGTGACTTCCGTTGTGCTCAAATATGATCACAAATTCATAGAAAATATTGGATACGTTCTTTATGGAATATACAATGTAACTATGGTGGTCGTTTTACTCAACATGCTAATTGCTATGATTAATAGCTCATATCAAGAAATTGAGGATGACAGTGATGTAGAATGGAAGTTTGCTCGTTCAAAACTTTGGTTATCCTATTTTGATGATGGAAAAACATTACCTCCACCTTTCAGTCTAGTTCCTAGTCCAAAATCATTTGTTTATTTCATCATGCGAATTGTTAACTTTCCCAAATGCAGAAGGAGAAGGCTTCAGAAGGATATAGAAATGGGAATGGGTAACTCAAAGTCCAGGTTAAACCTCTTCACTCAGTCTAACTCAAGAGTTTTTGAATCACACAGTTTTAACAGCATTCTCAATCAGCCAACACGTTATCAGCAGATAATGAAAAGACTTATAAAGCGGTATGTTTTGAAAGCAGGCGCGCCTATGGTGAGCAAGGGCGAGGAGCTGTTCACCGGGGTGGTGCCCATCCTGGTCGAGCTGGACGGCGACGTAAACGGCCACAAGTTCAGCGTGTCCGGCGAGGGCGAGGGCGATGCCACCTACGGCAAGCTGACCCTGAAGTTCATCTGCACCACCGGCAAGCTGCCCGTGCCCTGGCCCACCCTCGTGACCACCCTGACCTACGGCGTGCAGTGCTTCAGCCGCTACCCCGACCACATGAAGCAGCACGACTTCTTCAAGTCCGCCATGCCCGAAGGCTACGTCCAGGAGCGCACCATCTTCTTCAAGGACGACGGCAACTACAAGACCCGCGCCGAGGTGAAGTTCGAGGGCGACACCCTGGTGAACCGCATCGAGCTGAAGGGCATCGACTTCAAGGAGGACGGCAACATCCTGGGGCACAAGCTGGAGTACAACTACAACAGCCACAACGTCTATATCATGGCCGACAAGCAGAAGAACGGCATCAAGGTGAACTTCAAGATCCGCCACAACATCGAGGACGGCAGCGTGCAGCTCGCCGACCACTACCAGCAGAACACCCCCATCGGCGACGGCCCCGTGCTGCTGCCCGACAACCACTACCTGAGCACCCAGTCCGCCCTGAGCAAAGACCCCAACGAGAAGCGCGATCACATGGTCCTGCTGGAGTTCGTGACCGCCGCCGGGATCACTCTCGGCATGGACGAGCTGTACAAGTAA

**TRPC3-GFP 1-759**

ATGGAGGGAAGCCCATCCCTGAGACGCATGACAGTGATGCGGGAGAAGGGCCGGCGCCAGGCTGTCAGGGGCCCGGCCTTCATGTTCAATGACCGCGGCACCAGCCTCACCGCCGAGGAGGAGCGCTTCCTCGACGCCGCCGAGTACGGCAACATCCCAGTGGTGCGCAAGATGCTGGAGGAGTCCAAGACGCTGAACGTCAACTGCGTGGACTACATGGGCCAGAACGCGCTGCAGCTGGCTGTGGGCAACGAGCACCTGGAGGTGACCGAGCTGCTGCTCAAGAAGGAGAACCTGGCGCGCATTGGCGACGCCCTGCTGCTCGCCATCAGCAAGGGCTACGTGCGCATCGTAGAGGCCATCCTCAACCACCCTGGCTTCGCGGCCAGCAAGCGTCTCACTCTGAGCCCCTGTGAGCAGGAGCTGCAGGACGACGACTTCTACGCTTACGACGAGGACGGCACGCGCTTCTCGCCGGACATCACCCCCATCATCCTGGCGGCGCACTGCCAGAAATACGAAGTGGTGCACATGCTGCTGATGAAGGGTGCCAGGATCGAGCGGCCGCACGACTATTTCTGCAAGTGCGGGGACTGCATGGAGAAGCAGAGGCACGACTCCTTCAGCCACTCACGCTCGAGGATCAATGCCTACAAGGGGCTGGCCAGCCCGGCTTACCTCTCATTGTCCAGCGAGGACCCGGTGCTTACGGCCCTAGAGCTCAGCAACGAGCTGGCCAAGCTGGCCAACATAGAGAAGGAGTTCAAGAATGACTATCGGAAGCTCTCCATGCAATGCAAAGACTTTGTAGTGGGTGTGCTGGATCTCTGCCGAGACTCAGAAGAGGTAGAAGCCATTCTGAATGGAGATCTGGAATCAGCAGAGCCTCTGGAGGTACACAGGCACAAAGCTTCATTAAGTCGTGTCAAACTTGCCATTAAGTATGAAGTCAAAAAGTTTGTGGCTCATCCCAACTGCCAGCAGCAGCTCTTGACGATCTGGTATGAGAACCTCTCAGGCCTAAGGGAGCAGACCATAGCTATCAAGTGTCTCGTTGTGCTGGTCGTGGCCCTGGGCCTTCCATTCCTGGCCATTGGCTACTGGATCGCACCTTGCAGCAGGCTGGGGAAAATTCTGCGAAGCCCTTTTATGAAGTTTGTAGCACATGCAGCTTCTTTCATCATCTTCCTGGGTCTGCTTGTGTTCAATGCCTCAGACAGGTTCGAAGGCATCACCACGCTGCCCAATATCACAGTTACTGACTATCCCAAACAGATCTTCAGGGTGAAAACCACCCAGTTTACATGGACTGAAATGCTAATTATGGTCTGGGTTCTTGGAATGATGTGGTCTGAATGTAAAGAGCTCTGGCTGGAAGGACCTAGGGAATACATTTTGCAGTTGTGGAATGTGCTTGACTTTGGGATGCTGTCCATCTTCATTGCTGCTTTCACAGCCAGATTCCTAGCTTTCCTTCAGGCAACGAAGGCACAACAGTATGTGGACAGTTACGTCCAAGAGAGTGACCTCAGTGAAGTGACACTCCCACCAGAGATACAGTATTTCACTTATGCTAGAGATAAATGGCTCCCTTCTGACCCTCAGATTATATCTGAAGGCCTTTATGCCATAGCTGTTGTGCTCAGCTTCTCTCGGATTGCGTACATCCTCCCTGCAAATGAGAGCTTTGGCCCCCTGCAGATCTCTCTTGGAAGGACTGTAAAGGACATATTCAAGTTCATGGTCCTCTTTATTATGGTGTTTTTTGCCTTTATGATTGGCATGTTCATACTTTATTCTTACTACCTTGGGGCTAAAGTTAATGCTGCTTTTACCACTGTAGAAGAAAGTTTCAAGACTTTATTTTGGTCAATATTTGGGTTGTCTGAAGTGACTTCCGTTGTGCTCAAATATGATCACAAATTCATAGAAAATATTGGATACGTTCTTTATGGAATATACAATGTAACTATGGTGGTCGTTTTACTCAACATGCTAATTGCTATGATTAATAGCTCATATCAAGAAATTGAGGATGACAGTGATGTAGAATGGAAGTTTGCTCGTTCAAAACTTTGGTTATCCTATTTTGATGATGGAAAAACATTACCTCCACCTTTCAGTCTAGTTCCTAGTCCAAAATCATTTGTTTATTTCATCATGCGAATTGTTAACTTTCCCAAATGCAGAAGGAGAAGGCTTCAGAAGGATATAGAAATGGGAATGGGTAACTCAAAGTCCAGGTTAAACCTCTTCACTCAGTCTAACTCAAGAGTTGGCGCGCCTATGGTGAGCAAGGGCGAGGAGCTGTTCACCGGGGTGGTGCCCATCCTGGTCGAGCTGGACGGCGACGTAAACGGCCACAAGTTCAGCGTGTCCGGCGAGGGCGAGGGCGATGCCACCTACGGCAAGCTGACCCTGAAGTTCATCTGCACCACCGGCAAGCTGCCCGTGCCCTGGCCCACCCTCGTGACCACCCTGACCTACGGCGTGCAGTGCTTCAGCCGCTACCCCGACCACATGAAGCAGCACGACTTCTTCAAGTCCGCCATGCCCGAAGGCTACGTCCAGGAGCGCACCATCTTCTTCAAGGACGACGGCAACTACAAGACCCGCGCCGAGGTGAAGTTCGAGGGCGACACCCTGGTGAACCGCATCGAGCTGAAGGGCATCGACTTCAAGGAGGACGGCAACATCCTGGGGCACAAGCTGGAGTACAACTACAACAGCCACAACGTCTATATCATGGCCGACAAGCAGAAGAACGGCATCAAGGTGAACTTCAAGATCCGCCACAACATCGAGGACGGCAGCGTGCAGCTCGCCGACCACTACCAGCAGAACACCCCCATCGGCGACGGCCCCGTGCTGCTGCCCGACAACCACTACCTGAGCACCCAGTCCGCCCTGAGCAAAGACCCCAACGAGAAGCGCGATCACATGGTCCTGCTGGAGTTCGTGACCGCCGCCGGGATCACTCTCGGCATGGACGAGCTGTACAAGTAA

**TRPC3-YFP**

ATGGAGGGAAGCCCATCCCTGAGACGCATGACAGTGATGCGGGAGAAGGGCCGGCGCCAGGCTGTCAGGGGCCCGGCCTTCATGTTCAATGACCGCGGCACCAGCCTCACCGCCGAGGAGGAGCGCTTCCTCGACGCCGCCGAGTACGGCAACATCCCAGTGGTGCGCAAGATGCTGGAGGAGTCCAAGACGCTGAACGTCAACTGCGTGGACTACATGGGCCAGAACGCGCTGCAGCTGGCTGTGGGCAACGAGCACCTGGAGGTGACCGAGCTGCTGCTCAAGAAGGAGAACCTGGCGCGCATTGGCGACGCCCTGCTGCTCGCCATCAGCAAGGGCTACGTGCGCATCGTAGAGGCCATCCTCAACCACCCTGGCTTCGCGGCCAGCAAGCGTCTCACTCTGAGCCCCTGTGAGCAGGAGCTGCAGGACGACGACTTCTACGCTTACGACGAGGACGGCACGCGCTTCTCGCCGGACATCACCCCCATCATCCTGGCGGCGCACTGCCAGAAATACGAAGTGGTGCACATGCTGCTGATGAAGGGTGCCAGGATCGAGCGGCCGCACGACTATTTCTGCAAGTGCGGGGACTGCATGGAGAAGCAGAGGCACGACTCCTTCAGCCACTCACGCTCGAGGATCAATGCCTACAAGGGGCTGGCCAGCCCGGCTTACCTCTCATTGTCCAGCGAGGACCCGGTGCTTACGGCCCTAGAGCTCAGCAACGAGCTGGCCAAGCTGGCCAACATAGAGAAGGAGTTCAAGAATGACTATCGGAAGCTCTCCATGCAATGCAAAGACTTTGTAGTGGGTGTGCTGGATCTCTGCCGAGACTCAGAAGAGGTAGAAGCCATTCTGAATGGAGATCTGGAATCAGCAGAGCCTCTGGAGGTACACAGGCACAAAGCTTCATTAAGTCGTGTCAAACTTGCCATTAAGTATGAAGTCAAAAAGTTTGTGGCTCATCCCAACTGCCAGCAGCAGCTCTTGACGATCTGGTATGAGAACCTCTCAGGCCTAAGGGAGCAGACCATAGCTATCAAGTGTCTCGTTGTGCTGGTCGTGGCCCTGGGCCTTCCATTCCTGGCCATTGGCTACTGGATCGCACCTTGCAGCAGGCTGGGGAAAATTCTGCGAAGCCCTTTTATGAAGTTTGTAGCACATGCAGCTTCTTTCATCATCTTCCTGGGTCTGCTTGTGTTCAATGCCTCAGACAGGTTCGAAGGCATCACCACGCTGCCCAATATCACAGTTACTGACTATCCCAAACAGATCTTCAGGGTGAAAACCACCCAGTTTACATGGACTGAAATGCTAATTATGGTCTGGGTTCTTGGAATGATGTGGTCTGAATGTAAAGAGCTCTGGCTGGAAGGACCTAGGGAATACATTTTGCAGTTGTGGAATGTGCTTGACTTTGGGATGCTGTCCATCTTCATTGCTGCTTTCACAGCCAGATTCCTAGCTTTCCTTCAGGCAACGAAGGCACAACAGTATGTGGACAGTTACGTCCAAGAGAGTGACCTCAGTGAAGTGACACTCCCACCAGAGATACAGTATTTCACTTATGCTAGAGATAAATGGCTCCCTTCTGACCCTCAGATTATATCTGAAGGCCTTTATGCCATAGCTGTTGTGCTCAGCTTCTCTCGGATTGCGTACATCCTCCCTGCAAATGAGAGCTTTGGCCCCCTGCAGATCTCTCTTGGAAGGACTGTAAAGGACATATTCAAGTTCATGGTCCTCTTTATTATGGTGTTTTTTGCCTTTATGATTGGCATGTTCATACTTTATTCTTACTACCTTGGGGCTAAAGTTAATGCTGCTTTTACCACTGTAGAAGAAAGTTTCAAGACTTTATTTTGGTCAATATTTGGGTTGTCTGAAGTGACTTCCGTTGTGCTCAAATATGATCACAAATTCATAGAAAATATTGGATACGTTCTTTATGGAATATACAATGTAACTATGGTGGTCGTTTTACTCAACATGCTAATTGCTATGATTAATAGCTCATATCAAGAAATTGAGGATGACAGTGATGTAGAATGGAAGTTTGCTCGTTCAAAACTTTGGTTATCCTATTTTGATGATGGAAAAACATTACCTCCACCTTTCAGTCTAGTTCCTAGTCCAAAATCATTTGTTTATTTCATCATGCGAATTGTTAACTTTCCCAAATGCAGAAGGAGAAGGCTTCAGAAGGATATAGAAATGGGAATGGGTAACTCAAAGTCCAGGTTAAACCTCTTCACTCAGTCTAACTCAAGAGTTTTTGAATCACACAGTTTTAACAGCATTCTCAATCAGCCAACACGTTATCAGCAGATAATGAAAAGACTTATAAAGCGGTATGTTTTGAAAGCACAAGTAGACAAAGAAAATGATGAAGTTAATGAAGGTGAATTAAAAGAAATCAAGCAAGATATCTCCAGCCTTCGTTATGAACTTTTGGAAGACAAGAGCCAAGCAACTGAGGAATTAGCCATTCTAATTCATAAACTTAGTGAGAAACTGAATCCCAGCATGCTGAGATGTGAAGGCGCGCCTATGGTAAGTAAGGGCGATGATCTGTTCACCGGAGTGGTGCCCATCCTGGTCGAGCTGGACGGCGACGTAAACGGCCACAAGTTCAGCGTGTCCGGCGAGGGCGAGGGCGATGCCACCTACGGCAAGCTGACCCTGAAGTTCATCTGCACCACCGGCAAGCTGCCCGTGCCCTGGCCCACCCTCGTGACCACCTTCGGCTACGGCCTGCAGTGCTTCGCCCGCTACCCCGACCACATGAAGCAGCACGACTTCTTCAAGTCCGCCATGCCCGAAGGCTACGTCCAGGAGCGCACCATCTTCTTCAAGGACGACGGCAACTACAAGACCCGCGCCGAGGTGAAGTTCGAGGGCGACACCCTGGTGAACCGCATCGAGCTGAAGGGCATCGACTTCAAGGAGGACGGCAACATCCTGGGGCACAAGCTGGAGTACAACTACAACAGCCACAACGTCTATATCATGGCCGACAAGCAGAAGAACGGCATCAAGGTGAACTTCAAGATCCGCCACAACATCGAGGACGGCAGCGTGCAGCTCGCCGACCACTACCAGCAGAACACCCCCATCGGCGACGGCCCCGTGCTGCTGCCCGACAACCACTACCTGAGCTACCAGTCCGCCCTGAGCAAAGACCCCAACGAGAAGCGCGATCACATGGTCCTGCTGGAGTTCGTGACCGCCGCCGGGATCACTCTCGGCATGGACGAGCTGTACAAGTAA

**Luc-TRPC3-YFP**

atgaccagcaaggtgtacgaccccgagcagaggaagaggatgatcaccggcccccagtggtgggccaggtgcaagcagatgaacgtgctggacagcttcatcaactactacgacagcgagaagcacgccgagaacgccgtgatcttcctgcacggcaacgccgctagcagctacctgtggaggcacgtggtgccccacatcgagcccgtggccaggtgcatcatccccgatctgatcggcatgggcaagagcggcaagagcggcaacggcagctacaggctgctggaccactacaagtacctgaccgcctggttcgagctcctgaacctgcccaagaagatcatcttcgtgggccacgactggggcgcctgcctggccttccactacagctacgagcaccaggacaagatcaaggccatcgtgcacgccgagagcgtggtggacgtgatcgagagctgggacgagtggccagacatcgaggaggacatcgccctgatcaagagcgaggagggcgagaagatggtgctggagaacaacttcttcgtggagaccatgctgcccagcaagatcatgagaaagctggagcccgaggagttcgccgcctacctggagcccttcaaggagaagggcgaggtgagaagacccaccctgagctggcccagagagatccccctggtgaagggcggcaagcccgacgtggtgcagatcgtgagaaactacaacgcctacctgagagccagcgacgacctgcccaagatgttcatcgagagcgaccccggcttcttcagcaacgccatcgtggagggcgccaagaagttccccaacaccgagttcgtgaaggtgaagggcctgcacttcagccaggaggacgcccccgacgagatgggcaagtacatcaagagcttcgtggagagagtgctgaagaacgagcagggggatctccggcgagctctcgagaattctcacgcgtctgcaggatatcaagcttgcggtaccggatccATGGAGGGAAGCCCATCCCTGAGACGCATGACAGTGATGCGGGAGAAGGGCCGGCGCCAGGCTGTCAGGGGCCCGGCCTTCATGTTCAATGACCGCGGCACCAGCCTCACCGCCGAGGAGGAGCGCTTCCTCGACGCCGCCGAGTACGGCAACATCCCAGTGGTGCGCAAGATGCTGGAGGAGTCCAAGACGCTGAACGTCAACTGCGTGGACTACATGGGCCAGAACGCGCTGCAGCTGGCTGTGGGCAACGAGCACCTGGAGGTGACCGAGCTGCTGCTCAAGAAGGAGAACCTGGCGCGCATTGGCGACGCCCTGCTGCTCGCCATCAGCAAGGGCTACGTGCGCATCGTAGAGGCCATCCTCAACCACCCTGGCTTCGCGGCCAGCAAGCGTCTCACTCTGAGCCCCTGTGAGCAGGAGCTGCAGGACGACGACTTCTACGCTTACGACGAGGACGGCACGCGCTTCTCGCCGGACATCACCCCCATCATCCTGGCGGCGCACTGCCAGAAATACGAAGTGGTGCACATGCTGCTGATGAAGGGTGCCAGGATCGAGCGGCCGCACGACTATTTCTGCAAGTGCGGGGACTGCATGGAGAAGCAGAGGCACGACTCCTTCAGCCACTCACGCTCGAGGATCAATGCCTACAAGGGGCTGGCCAGCCCGGCTTACCTCTCATTGTCCAGCGAGGACCCGGTGCTTACGGCCCTAGAGCTCAGCAACGAGCTGGCCAAGCTGGCCAACATAGAGAAGGAGTTCAAGAATGACTATCGGAAGCTCTCCATGCAATGCAAAGACTTTGTAGTGGGTGTGCTGGATCTCTGCCGAGACTCAGAAGAGGTAGAAGCCATTCTGAATGGAGATCTGGAATCAGCAGAGCCTCTGGAGGTACACAGGCACAAAGCTTCATTAAGTCGTGTCAAACTTGCCATTAAGTATGAAGTCAAAAAGTTTGTGGCTCATCCCAACTGCCAGCAGCAGCTCTTGACGATCTGGTATGAGAACCTCTCAGGCCTAAGGGAGCAGACCATAGCTATCAAGTGTCTCGTTGTGCTGGTCGTGGCCCTGGGCCTTCCATTCCTGGCCATTGGCTACTGGATCGCACCTTGCAGCAGGCTGGGGAAAATTCTGCGAAGCCCTTTTATGAAGTTTGTAGCACATGCAGCTTCTTTCATCATCTTCCTGGGTCTGCTTGTGTTCAATGCCTCAGACAGGTTCGAAGGCATCACCACGCTGCCCAATATCACAGTTACTGACTATCCCAAACAGATCTTCAGGGTGAAAACCACCCAGTTTACATGGACTGAAATGCTAATTATGGTCTGGGTTCTTGGAATGATGTGGTCTGAATGTAAAGAGCTCTGGCTGGAAGGACCTAGGGAATACATTTTGCAGTTGTGGAATGTGCTTGACTTTGGGATGCTGTCCATCTTCATTGCTGCTTTCACAGCCAGATTCCTAGCTTTCCTTCAGGCAACGAAGGCACAACAGTATGTGGACAGTTACGTCCAAGAGAGTGACCTCAGTGAAGTGACACTCCCACCAGAGATACAGTATTTCACTTATGCTAGAGATAAATGGCTCCCTTCTGACCCTCAGATTATATCTGAAGGCCTTTATGCCATAGCTGTTGTGCTCAGCTTCTCTCGGATTGCGTACATCCTCCCTGCAAATGAGAGCTTTGGCCCCCTGCAGATCTCTCTTGGAAGGACTGTAAAGGACATATTCAAGTTCATGGTCCTCTTTATTATGGTGTTTTTTGCCTTTATGATTGGCATGTTCATACTTTATTCTTACTACCTTGGGGCTAAAGTTAATGCTGCTTTTACCACTGTAGAAGAAAGTTTCAAGACTTTATTTTGGTCAATATTTGGGTTGTCTGAAGTGACTTCCGTTGTGCTCAAATATGATCACAAATTCATAGAAAATATTGGATACGTTCTTTATGGAATATACAATGTAACTATGGTGGTCGTTTTACTCAACATGCTAATTGCTATGATTAATAGCTCATATCAAGAAATTGAGGATGACAGTGATGTAGAATGGAAGTTTGCTCGTTCAAAACTTTGGTTATCCTATTTTGATGATGGAAAAACATTACCTCCACCTTTCAGTCTAGTTCCTAGTCCAAAATCATTTGTTTATTTCATCATGCGAATTGTTAACTTTCCCAAATGCAGAAGGAGAAGGCTTCAGAAGGATATAGAAATGGGAATGGGTAACTCAAAGTCCAGGTTAAACCTCTTCACTCAGTCTAACTCAAGAGTTTTTGAATCACACAGTTTTAACAGCATTCTCAATCAGCCAACACGTTATCAGCAGATAATGAAAAGACTTATAAAGCGGTATGTTTTGAAAGCACAAGTAGACAAAGAAAATGATGAAGTTAATGAAGGTGAATTAAAAGAAATCAAGCAAGATATCTCCAGCCTTCGTTATGAACTTTTGGAAGACAAGAGCCAAGCAACTGAGGAATTAGCCATTCTAATTCATAAACTTAGTGAGAAACTGAATCCCAGCATGCTGAGATGTGAAGGCGCGCCTATGGTAAGTAAGGGCGATGATCTGTTCACCGGAGTGGTGCCCATCCTGGTCGAGCTGGACGGCGACGTAAACGGCCACAAGTTCAGCGTGTCCGGCGAGGGCGAGGGCGATGCCACCTACGGCAAGCTGACCCTGAAGTTCATCTGCACCACCGGCAAGCTGCCCGTGCCCTGGCCCACCCTCGTGACCACCTTCGGCTACGGCCTGCAGTGCTTCGCCCGCTACCCCGACCACATGAAGCAGCACGACTTCTTCAAGTCCGCCATGCCCGAAGGCTACGTCCAGGAGCGCACCATCTTCTTCAAGGACGACGGCAACTACAAGACCCGCGCCGAGGTGAAGTTCGAGGGCGACACCCTGGTGAACCGCATCGAGCTGAAGGGCATCGACTTCAAGGAGGACGGCAACATCCTGGGGCACAAGCTGGAGTACAACTACAACAGCCACAACGTCTATATCATGGCCGACAAGCAGAAGAACGGCATCAAGGTGAACTTCAAGATCCGCCACAACATCGAGGACGGCAGCGTGCAGCTCGCCGACCACTACCAGCAGAACACCCCCATCGGCGACGGCCCCGTGCTGCTGCCCGACAACCACTACCTGAGCTACCAGTCCGCCCTGAGCAAAGACCCCAACGAGAAGCGCGATCACATGGTCCTGCTGGAGTTCGTGACCGCCGCCGGGATCACTCTCGGCATGGACGAGCTGTACAAGTAA

**Flag-AT1R-cherry**

ATGGACTACAAGGACGATGATGACAAAATGATTCTCAACTCTTCTACTGAAGATGGTATTAAAAGAATCCAAGATGATTGTCCCAAAGCTGGAAGGCATAATTACATATTTGTCATGATTCCTACTTTATACAGTATCATCTTTGTGGTGGGAATATTTGGAAACAGCTTGGTGGTGATAGTCATTTACTTTTATATGAAGCTGAAGACTGTGGCCAGTGTTTTTCTTTTGAATTTAGCACTGGCTGACTTATGCTTTTTACTGACTTTGCCACTATGGGCTGTCTACACAGCTATGGAATACCGCTGGCCCTTTGGCAATTACCTATGTAAGATTGCTTCAGCCAGCGTCAGTTTCAACCTGTACGCTAGTGTGTTTCTACTCACGTGTCTCAGCATTGATCGATACCTGGCTATTGTTCACCCAATGAAGTCCCGCCTTCGACGCACAATGCTTGTAGCCAAAGTCACCTGCATCATCATTTGGCTGCTGGCAGGCTTGGCCAGTTTGCCAGCTATAATCCATCGAAATGTATTTTTCATTGAGAACACCAATATTACAGTTTGTGCTTTCCATTATGAGTCCCAAAATTCAACCCTCCCGATAGGGCTGGGCCTGACCAAAAATATACTGGGTTTCCTGTTTCCTTTTCTGATCATTCTTACAAGTTATACTCTTATTTGGAAGGCCCTAAAGAAGGCTTATGAAATTCAGAAGAACAAACCAAGAAATGATGATATTTTTAAGATAATTATGGCAATTGTGCTTTTCTTTTTCTTTTCCTGGATTCCCCACCAAATATTCACTTTTCTGGATGTATTGATTCAACTAGGCATCATACGTGACTGTAGAATTGCAGATATTGTGGACACGGCCATGCCTATCACCATTTGTATAGCTTATTTTAACAATTGCCTGAATCCTCTTTTTTATGGCTTTCTGGGGAAAAAATTTAAAAGATATTTTCTCCAGCTTCTAAAATATATTCCCCCAAAAGCCAAATCCCACTCAAACCTTTCAACAAAAATGAGCACGCTTTCCTACCGCCCCTCAGATAATGTAAGCTCATCCACCAAGAAGCCTGCACCATGTTTTGAGGTTGAG ATGGTCTCTAAGGGCGAGGAAGACAACATGGCTATCATCAAAGAGTTCATGCGGTTCAAGGTCCACATGGAGGGCTCTGTCAACGGGCACGAGTTTGAAATAGAAGGGGAAGGGGAGGGGCGGCCCTACGAAGGAACCCAAACCGCCAAGCTGAAGGTTACCAAAGGCGGGCCCCTGCCCTTTGCCTGGGACATACTGTCCCCTCAGTTTATGTACGGCTCTAAAGCCTATGTTAAACATCCTGCCGATATACCCGATTATCTGAAGCTCTCTTTTCCAGAAGGATTTAAATGGGAGCGGGTCATGAATTTCGAGGATGGCGGCGTGGTGACCGTGACCCAGGATTCTAGCCTCCAGGATGGGGAGTTTATATATAAGGTGAAGCTGCGGGGAACCAATTTCCCAAGCGACGGGCCTGTGATGCAGAAGAAGACAATGGGTTGGGAGGCCAGCTCTGAACGGATGTATCCTGAGGATGGAGCCCTGAAGGGTGAGATTAAACAGAGGCTGAAGCTCAAGGACGGCGGCCATTATGATGCCGAAGTCAAAACAACTTACAAGGCTAAGAAGCCAGTCCAGCTCCCCGACGCATATAATGTCAATATCAAACTGGACATTACTTCTCATAATGAGGACTACACAATCGTGGAGCAATACGAGCGGGCCGAAGGCAGGCATTCTACCGGTGGCATGGATGAGCTGTACAAATAA

**Flag-AT1R**

ATGGACTACAAGGACGATGATGACAAAATGATTCTCAACTCTTCTACTGAAGATGGTATTAAAAGAATCCAAGATGATTGTCCCAAAGCTGGAAGGCATAATTACATATTTGTCATGATTCCTACTTTATACAGTATCATCTTTGTGGTGGGAATATTTGGAAACAGCTTGGTGGTGATAGTCATTTACTTTTATATGAAGCTGAAGACTGTGGCCAGTGTTTTTCTTTTGAATTTAGCACTGGCTGACTTATGCTTTTTACTGACTTTGCCACTATGGGCTGTCTACACAGCTATGGAATACCGCTGGCCCTTTGGCAATTACCTATGTAAGATTGCTTCAGCCAGCGTCAGTTTCAACCTGTACGCTAGTGTGTTTCTACTCACGTGTCTCAGCATTGATCGATACCTGGCTATTGTTCACCCAATGAAGTCCCGCCTTCGACGCACAATGCTTGTAGCCAAAGTCACCTGCATCATCATTTGGCTGCTGGCAGGCTTGGCCAGTTTGCCAGCTATAATCCATCGAAATGTATTTTTCATTGAGAACACCAATATTACAGTTTGTGCTTTCCATTATGAGTCCCAAAATTCAACCCTCCCGATAGGGCTGGGCCTGACCAAAAATATACTGGGTTTCCTGTTTCCTTTTCTGATCATTCTTACAAGTTATACTCTTATTTGGAAGGCCCTAAAGAAGGCTTATGAAATTCAGAAGAACAAACCAAGAAATGATGATATTTTTAAGATAATTATGGCAATTGTGCTTTTCTTTTTCTTTTCCTGGATTCCCCACCAAATATTCACTTTTCTGGATGTATTGATTCAACTAGGCATCATACGTGACTGTAGAATTGCAGATATTGTGGACACGGCCATGCCTATCACCATTTGTATAGCTTATTTTAACAATTGCCTGAATCCTCTTTTTTATGGCTTTCTGGGGAAAAAATTTAAAAGATATTTTCTCCAGCTTCTAAAATATATTCCCCCAAAAGCCAAATCCCACTCAAACCTTTCAACAAAAATGAGCACGCTTTCCTACCGCCCCTCAGATAATGTAAGCTCATCCACCAAGAAGCCTGCACCATGTTTTGAGGTTGAGTAA

**Lyn-YFP**

ATGGGATGTATAAAATCAAAAGGGAAAGACAGCGCACTGGTGAGCAAGGGCGAGGAGCTGTTCACCGGGGTGGTGCCCATCCTGGTCGAGCTGGACGGCGACGTAAACGGCCACAAGTTCAGCGTGTCCGGCGAGGGCGAGGGCGATGCCACCTACGGCAAGCTGACCCTGAAGTTCATCTGCACCACCGGCAAGCTGCCCGTGCCCTGGCCCACCCTCGTGACCACCTTCGGCTACGGCCTGAAGTGCTTCGCCCGCTACCCCGACCACATGAAGCAGCACGACTTCTTCAAGTCCGCCATGCCCGAAGGCTACGTCCAGGAGCGCACCATCTTCTTCAAGGACGACGGCAACTACAAGACCCGCGCCGAGGTGAAGTTCGAGGGCGACACCCTGGTGAACCGCATCGAGCTGAAGGGCATCGACTTCAAGGAGGACGGCAACATCCTGGGGCACAAGCTGGAGTACAACTACAACAGCCACAACGTCTATATCATGGCCGACAAGCAGAAGAACGGCATCAAGGTGAACTTCAAGATCCGCCACAACATCGAGGACGGCAGCGTGCAGCTCGCCGACCACTACCAGCAGAACACCCCCATCGGCGACGGCCCCGTGCTGCTGCCCGACAACCACTACCTGAGCTACCAGTCCAAGCTGAGCAAAGACCCCAACGAGAAGCGCGATCACATGGTCCTGCTGGAGTTCGTGACCGCCGCCGGGATCACTCACGGCATGGACGAGCTGTACAAGTAA
